# Supplementary material for: Plasmodium falciparum is evolving to escape malaria rapid diagnostic tests in Ethiopia
Source: Nat Microbiol. 2021 Sep 27;6(10):1289–99. doi: 10.1038/s41564-021-00962-4 (PMC8478644; doi:10.1038/s41564-021-00962-4)
Supplement: Supplementary file 1 — Supplementary Note, Tables 1–11 and PCR conditions. [file 41564_2021_962_MOESM1_ESM.pdf]

nature portfolio

https://doi.org/10.1038/s41564-021-00962-4

Supplementary information

Plasmodium falciparum is evolving to escape malaria rapid diagnostic tests in Ethiopia

In the format provided by the authors and unedited

SUPPLEMENTARY INFORMATION

Plasmodium falciparum is evolving to escape malaria rapid diagnostic tests in Ethiopia

Sindew M. Feleke[1a*], Emily N. Reichert[2a], Hussein Mohammed[1], Bokretsion G. Brhane[1], Kalkidan Mekete[1], Hassen Mamo[3], Beyene Petros[3], Hiwot Solomon[4], Ebba Abate[1], Chris Hennelly[2], Madeline Denton[2], Corinna Keeler[2], Nicholas J. Hathaway[5], Jonathan J. Juliano[2], Jeffrey A. Bailey[6], Eric Rogier[7], Jane Cunningham[8b*], Ozkan Aydemir[6b], Jonathan B. Parr[2b*]
([a] These authors contributed equally; [b] These authors jointly supervised the work; * Corresponding authors)

Affiliations

1. Ethiopian Public Health Institute (EPHI), Addis Ababa, Ethiopia
2. Institute for Global Health and Infectious Diseases and Department of Medicine, Division of Infectious Diseases, University of North Carolina at Chapel Hill (UNC), USA
3. Department of Microbial, Cellular and Molecular Biology, College of Natural and Computational Sciences, Addis Ababa University, Addis Ababa, Ethiopia
4. Ministry of Health (MoH), Addis Ababa, Ethiopia
5. Department of Medicine, University of Massachusetts Medical School, Worcester, Massachusetts, USA
6. Department of Pathology and Laboratory Medicine, Warren Alpert Medical School, Brown University, Providence, RI, USA
7. Division of Parasitic Diseases and Malaria, Center for Disease Control and Prevention (CDC), Atlanta, USA
8. Global Malaria Programme, World Health Organization (WHO), Geneva, Switzerland

* Corresponding authors

Sindew M. Feleke, MSc: sindewm@gmail.com; 251-978803070; Ethiopian Public Health Institute, PO Box 1242/5654, Addis Ababa, Ethiopia.

Jane Cunningham, MD: cunninghamj@who.int; 41-227912230; World Health Organization, WHO/UCN/GMP/DMR, 20 Avenue Appia, 1211 Geneva, Switzerland.

Jonathan B. Parr, MD, MPH: jonathan_parr@med.unc.edu; 1-9194451132; University of North Carolina at Chapel Hill, 111 Mason Farm Rd, Medical Biomolecular Research Building 2336, Chapel Hill, NC 27599, USA.

TABLE OF CONTENTS

Supplementary Note……………………………………………………………………………. 3

Supplementary Tables…………………………………………………………………….…. 10

We recommend viewing the Supplementary Tables in the companion Excel file rather than in pdf format for ease of viewing:

Supplementary Table 1.	PCR results by age, sex, and parasite density.
Supplementary Table 2.	RDT profile by district for individuals *P. falciparum*-positive by RDT.
Supplementary Table 3.	Prevalence estimate sensitivity analysis.
Supplementary Table 4.	MIP subtelomeric structural profiles by region.
Supplementary Table 5.	*Pfhrp2/3* MIP panel design overview, including genes targeted, MIPs designed, and genomic coordinates.
Supplementary Table 6.	*Pfhrp2/3* MIP panel probe sequences.
Supplementary Table 7.	Absolute (A) and normalized (B) unique molecular identifier (UMI) counts by sample and locus.
Supplementary Table 8.	MIP variant calls, which were converted to .map and .hap files.
Supplementary Table 9.	ENA accession numbers of previously published WGS data.
Supplementary Table 10.	Summary statistics of WGS coverage for samples sequenced in this study.
Supplementary Table 11.	RDT results from 12,572 enrolled subjects.

PCR Conditions……………………………………………………………………………….. 33

References…….………………………………………………………………………….….. 34

SUPPLEMENTARY NOTE

Samples subjected to molecular analysis

820 samples with complete demographic and clinical data underwent molecular analysis, including a randomly selected 248/361 (68.7%) of those with the discordant RDT profile of interest (HRP2-, Pf-LDH+), as well as 465/2115 (22.0%) randomly selected *P. falciparum* RDT HRP2 positives (HRP2+, Pf-LDH+) as controls. The remaining 107 samples included 90 with inconclusive HRP2 results (HRP2+ by only 1 RDT, of which 67 were Pf-LDH- and 23 were Pf-LDH+) and 17 negative controls (HRP2-, Pf-LDH-). Quantitative real-time PCR (qPCR) targeting the *P. falciparum* lactate dehydrogenase (*pfldh*) gene confirmed parasitemia in 731/820 (89%) samples, with a geometric mean (GM) of 1,390.7 parasites/µL (geometric standard deviation [geoSD]: 9.8).

Comparison of pfhrp2/3 PCR, RDT, and HRP2 bead-based assays

Concordance between *pfhrp2*-negative PCR results and the discordant RDT profile was good (Cohen's kappa 0.66). Overall, among samples with the discordant RDT profile, 64.8% (95% CI 57-72) were *pfhrp2-/3-* and 8.0% (95% CI 5-13) *pfhrp2-/3+*, with an additional 15.9% (95% CI 11-22) *pfhrp2+/3-* (**Extended Data Figure 2**). Interestingly, of samples HRP2+ by both RDTs, *pfhrp3* could not be amplified in 42.6% (95% CI 38-48).

We observed expected agreement between the results of *pfhrp2/3* PCR assays, RDTs, and a bead-based immunoassay applied to a subset of 456 samples. 93% (95% CI 86-96) of samples *pfhrp2+/3+* by PCR tested positive for HRP2 antigen (GM 40,284 pg/mL HRP2, geoSD 7.5). In comparison, 19% (12-29) of *pfhrp2-/3-* samples were HRP2+, with a GM of 2,089 pg/mL HRP2 (geoSD 5.5).

HRP2+ but *pfhrp2-* PCR results are expected in a subset of subjects because HRP2 antigenemia can persist for weeks after clearance of parasitemia[1]. 92% (95% CI 88-95) of samples HRP2+, Pf-LDH+ by RDT were HRP2+ by the antigen assay (GM 34,536 pg/mL, geoSD 6.5), compared to 24% (95% CI 18-31) of those with the discordant HRP2-, Pf-LDH+ RDT profile of interest (GM 2,455 pg/mL, geoSD: 7.2) (**Table 2**).

Pf-LDH RDT performance

Despite the prevalence of *pfhrp2*-deleted parasites in this study, use of the SD Bioline's Pf-LDH RDT band rather than its HRP2 band did not improve RDT performance. Among 820 samples with RDT results and *pfldh* real-time PCR results available, overall agreement with PCR was slight regardless of test band used (Cohen's kappa 0.04 for the Pf-LDH band and 0.15 for the HRP2 band). Using *pfldh* PCR as the gold standard, the sensitivity and specificity of the Pf-LDH RDT band were 69% and 38%, respectively. The sensitivity and specificity of the HRP2 RDT band were 62% and 72%, respectively. The poor performance of the Pf-LDH RDT band was unexpected, as the RDTs used in this study passed WHO lot testing prior to shipment. Comparison of RDT results within the larger cohort (**Supplementary Table 11**) suggests several explanations, including decreased performance due to inappropriate storage conditions, operational or administrative user error (Pf-LDH test bands are often fainter than HRP2, especially at lower parasite densities), cross-reactivity with other species (though only in a minority of cases as outlined below, co-infections, and/or immunological factors.

For overall detection of *P. falciparum* parasitemia using any falciparum-specific antigen, the SD and CareStart products had similar levels of agreement with

pfldh PCR. Compared to PCR, the sensitivity and specificity of the SD RDT (HRP2+ and/or Pf-LDH+) were 93% and 20% (Cohen's kappa 0.14), respectively; the sensitivity and specificity of the CareStart RDT (HRP2+) were 69% and 60% (Cohen's kappa 0.15), respectively. However, because this study purposefully oversampled subjects at high risk for infection by *pfhrp2/3*-deleted parasites for molecular testing, these estimates should not be generalized to routine care in these regions.

Plasmodium vivax co-infection and pfhrp2/3 deletion

Though analysis of *P. vivax* co-infection was not an objective of this study, analysis of available RDT data did not reveal evidence of selection for *pfhrp2* deletion in the presence of *P. vivax* co-infection. First, the frequency of *pfhrp2* deletion was similar among subjects with versus without *P. vivax* co-infection. Among subjects who underwent *pfhrp2/3* PCR genotyping, *P. vivax* co-infection was identified by the CareStart RDT (Pv-LDH+) in 56 subjects. Of these, 41 (73.2%) were *pfhrp2*+ and 15 (26.8%) were *pfhrp2-.* Among all 608 samples with CareStart RDT results who were subjected to *pfhrp2/3* PCR genotyping, 445 (73.2%) were *pfhrp2*+ and 163 (26.8%) were *pfhrp2-.* Second, we did not observe an association between *P. vivax* co-infection and subtelomeric deletion structural profile. While there was only a single *pfhrp2* deletion profile observed in this study, three distinct *pfhrp3* deletion profiles were observed. The frequency of *P. vivax* co-infection was similar in the two dominant *pfhrp3* deletion subtelomeric structural profiles. 14 of 134 (10.4%) subjects with the *P. falciparum* chr13-P4 (*pfhrp3* deletion) profile and 6 of 106 (5.7%) with the chr13-P3 (*pfhrp3* deletion) profile had co-infection with *P. vivax.*

Subtelomeric profiling and variant calling using MIPs

The MIP panel was designed using a tiled strategy that involved multiple, overlapping probes spanning each gene target. We detected 244 of 273 targets with sufficient mapping quality and depth across multiple segments of both *pfhrp2* and *pfhrp3* and their flanking regions, spanning positions 1,344,451 to 1,397,773 and 2,780,863 to 2,853,533 of chromosomes 8 and 13, respectively. Fourteen total probes were used to target different segments of *pfhrp2* (n = 8 probes) and *pfhrp3* (n = 6). 241 MIPs mapped to 273 targeted loci on the reference genome, including 32 extra loci accounting for the multicopy genes on chromosome 11.

In total, 43,541,045 reads were devoted to this sample set, or roughly half of a single NextSeq 550 mid-output flow cell. Probes failing to amplify in >90% of the samples were removed from the analysis, leaving 244 loci. 841 of 1014 samples and controls had sequence data after read mapping. 20 of 841 belonged to control strains (positive controls). None of the 20 negative controls had any sequence mapping to the reference genome. Deletion calls were only made in samples with sufficient depth of UMI coverage (see Methods), leaving 375 high-coverage samples from the study cohort and 6 controls in the final call set.

The median parasite density for samples successfully called using MIP sequencing was 5,077 p/µL (SD: 1.6×10^4), compared to 264 p/µL (SD: 5.9×10^3) for samples with failed MIP calls. Among 367 (97.9%) MIP-called samples with matching PCR data, 85 (23.2%) were *pfhrp2-/3-* by PCR. Receiver-operator curve analysis indicated the optimal parasite density threshold above which samples had sufficient coverage for MIP calling was approximately 925 p/µL, although this threshold is project-specific and expected to improve with additional sequencing effort (**Extended Data Figure 4**).

Comparison of pfhrp2/3 MIP sequencing to PCR, HRP2 immunoassay, and whole-genome sequencing results

Among 367 (97.9%) MIP-called samples with matching PCR data, 85 (23.2%) were *pfhrp2-/3-* by PCR. MIP sequencing results indicated that 126/367 (34.3%; 95% CI 30-39) were *pfhrp2-,* 264/367 (71.9%; 95% CI 67-76) *pfhrp3-,* and 116/367 (31.6%; 95% CI 27-37) *pfhrp2-/3-* by MIP sequencing. Of samples called *pfhrp2-* by MIP sequencing, 82.0% were *pfhrp2-* by PCR and 73.3% had the discordant RDT profile. Similarly, of samples called *pfhrp3-* by MIP sequencing, 76.7% were *pfhrp3-* by PCR. Differences between genotyping results were apparent and expected due to differences in targets and methodologies (PCR is better suited for samples with low parasite density than MIP sequencing). Comparison of results from MIP and PCR *pfhrp2/3* deletion genotyping revealed excellent agreement between the two approaches for *pfhrp2* (Cohen's kappa 0.82) and good agreement for *pfhrp3* (Cohen's kappa 0.63).

Comparison to the bead-based HRP2 immunoassay results provided additional confidence in the validity of our *pfhrp2/3* deletion calls using MIP sequencing. 175 MIP-called samples also had bead-based antigen detection results available. Despite fundamental differences in the targets of these two approaches (*pfhrp2* gene versus HRP2 antigen, which can linger after clearance of infection),[1] observed agreement between the two methods was consistent with expectation. Of those samples *pfhrp2*+ by MIP sequencing, 94.1% were HRP2+ by bead-based antigen immunoassay, whereas 79.5% of those *pfhrp2*- by MIPs were also HRP2- by the immunoassay.

We used whole-genome sequencing (WGS) to evaluate *pfhrp2/3* MIP sequencing results and breakpoint regions in a small validation subset. Among 14

samples subjected to WGS and with MIP calls, median WGS depth of coverage was 20 reads/locus (range 4-38). While the distribution of aligned reads was uneven in the regions flanking *pfhrp2* and *pfhrp3*, visual inspection of WGS coverage supported 13 (93%) *pfhrp2* and 14 (100%) *pfhrp3* deletion calls made using MIP sequencing data (**Figure 4**). For the single discordant *pfhrp2* deletion call (lab ID: 1314), *pfhrp2* PCR results were consistent with the MIP sequencing result. Precise mapping of breakpoint regions using WGS was not possible due to regions of very high coverage ("jackpotting") resulting from selective whole-genome amplification and low coverage due to ambiguous read mapping to repetitive and paralogous loci. However, breakpoint regions identified using MIPs were consistent with WGS coverage centromeric to *pfhrp2* and *pfhrp3* on chromosomes 8 and 13, respectively, with the exception of calls in chromosome 13's multi-copy 28S rRNA gene. Discordance in these calls was expected due to ambiguous mapping of short-read sequences to a multi-copy gene. MIP results from well-characterized lab strains 3D7 (*pfhrp2+/3+*), DD2 (*pfhrp2-/3+*), and HB3 (*pfhrp2+/3-*) were consistent with whole-genome alignments of published short-read data. Telomeric deletion breakpoint assessment was limited by a small number of successful MIP targets telomeric to both genes. However, the concordance in *pfhrp2/3* deletion calls and centromeric deletion breakpoint regions by MIP and WGS techniques confirmed the utility of MIPs for identifying *pfhrp2/3* deletions and determining their extent and breakpoint regions.

Association between malaria symptoms, geographical location, and subtelomeric structural variants

We did not observe an association between subtelomeric deletion profile and

the number of symptoms experienced by subjects (**Extended Data Figure 5**). Because the majority of subjects (96.5%, 2620/2714) who tested positive for *P. falciparum* by RDT were febrile, fever alone was not sufficient to evaluate disease severity. Therefore, as a crude metric of disease severity, we calculated the total number of symptoms (six total were assessed: fever, headache, joint pain, feeling cold, nausea, and lack of appetite). No significant differences in the total number of symptoms by deletion profile were revealed for chromosome 8 ($F(3, 334) = 0.29$, $p = 0.83$) or chromosome 13 ($F(2, 335) = 0.33$, $p = 0.72$) by one-way ANOVA. No obvious spatial patterns in subtelomeric deletion profiles were apparent at the regional level (**Supplementary Table 2**).

Extended haplotype homozygosity analysis

91 and 17 biallelic SNPs within the 28kb and 27kb regions centromeric to *pfhrp2* and *pfhrp3* deletions, respectively, were used to calculate EHH statistics. 327 samples with *pfhrp2* deletion calls using MIP sequencing and sufficient variant data were included in the EHH analysis, including 212 *pfhrp2*-intact and 115 *pfhrp2*-deleted haplotypes. 162 samples with *pfhrp3* deletion calls using MIP sequencing and sufficient variant data were included in the EHH analysis, including 37 *pfhrp3*-intact and 125 *pfhrp3*-deleted haplotypes with three distinct subtelomeric structural profiles. No variant calls were made in the 15.5 kb region immediately centromeric to *pfhrp3* to avoid ambiguity in read mapping to the duplicated DNA segment containing multicopy genes including 5.8S, 28S rRNA.

REFERENCES

1 Markwalter CF, Gibson LE, Mudenda L, Kimmel DW, Mbambara S, Thuma PE *et al.* Characterization of Plasmodium Lactate Dehydrogenase and Histidine-Rich Protein 2 Clearance Patterns via Rapid On-Bead Detection from a Single Dried Blood Spot. *Am J Trop Med Hyg* 2018; **98**: 1389–1396.

Supplementary Table 1. PCR results by age, sex, and parasite density. P values were calculated using chi-square (gender) and the non-parametric Kruskal-Wallis test (age, parasitemia).

	**PCR-based deletion calls**				**p value***	**test statistic**
	pfhrp2+/3+	***pfhrp2-/3-***	***pfhrp2-/3+***	***pfhrp2+/3-***		
N	253	136	27	192		
Sex = Female, N (%)	72 (29%)	48 (35%)	7 (26%)	59 (31%)	0.53	$X^2(3, N=610) = 2.24$
Age, Median (IQR)	19 (13)	18 (14)	16 (14)	19 (14)	0.76	$X^2(3, N=608) = 1.16$
Parasitemia (p/µL), Median (IQR)	2824 (7712)	3877 (8191)	2112 (6828)	2946 (8340)	0.14	$X^2(3, N=608) = 5.54$

Supplementary Table 2. RDT profile by district for individuals *P. falciparum*-positive by RDT (n=2,714). Abbreviations: Pf, *P. falciparum*; HRP2, histidine-rich protein 2; Pf-LDH, *P. falciparum*-specific lactate dehydrogenase; RDT, rapid diagnostic test.

		**RDT results**		
**Region** / District	**Total Pf+, n**	2 HRP2+, 1 PfLDH+	2 HRP2-, 1 PfLDH+	1 HRP2+, 1 HRP2-
**Tigray**	**689**	**460 (67%)**	**140 (20%)**	**85 (12%)**
Ahferom	117	83 (71%)	18 (15%)	16 (14%)
Atseged Tsimbila	160	86 (54%)	48 (30%)	26 (16%)
Gulomekeda	21	14 (67%)	5 (24%)	2 (10%)
K. Humera	176	115 (65%)	39 (22%)	21 (12%)
L. Adiabo	145	111 (77%)	22 (15%)	11 (8%)
T. Adiabo	69	51 (74%)	10 (14%)	6 (9%)
**Amhara**	**1,342**	**997 (74%)**	**211 (16%)**	**131 (10%)**
Metema	233	178 (76%)	30 (13%)	24 (10%)
Quara	516	435 (84%)	39 (8%)	42 (8%)
Tegede	296	197 (67%)	65 (22%)	33 (11%)
West Armachiho	295	187 (63%)	75 (25%)	32 (11%)
**Gambella**	**683**	**658 (96%)**	**10 (1%)**	**15 (2%)**
Itang	151	144 (95%)	2 (1%)	5 (3%)
Kule Refugee Camp	472	461 (98%)	4 (1%)	7 (1%)

Supplementary Table 3. Prevalence estimate sensitivity analysis. Abbreviations: CI, confidence interval; RDT, rapid diagnostic test; Pf-Pos, *P. falciparum*-positive by RDT.

Approach #1 (primary): Ci.impt function within asbio package, which calculates 95% CI of product of 2 proportions

	Pf-Pos, n	Discordant RDTs, n*	Discordant RDT prevalence, % (95% CI)	*pfhrp2*- PCR concordance (overall), %*	ci.impt prevalence estimate, % (95% CI)
Amhara	1342	211	15.8 (13.9-17.8)	72.7	11.5 (9.8-13.4)
Tigray	689	140	20.4 (17.5-23.7)	72.7	14.9 (12.5-17.7)
Gambella	683	10	1.5 (0.7-2.8)	72.7	1.1 (0.6-2.0)

Approach #2: Bootstrapping (1000 iterations)

	Pf-Pos, n	Discordant RDTs, n*	Discordant RDT prevalence, % (95% CI)	*pfhrp2*- PCR concordance (overall), %*	Estimated N with discordant RDT and pfhrp2- PCR result	Prevalence estimate, %	Bootstrap 95% CI
Amhara	1342	211	15.8 (13.9-17.8)	72.7	153.397	11.5	9.7-13.2
Tigray	689	140	20.4 (17.5-23.7)	72.7	101.78	14.9	12.2-17.4
Gambella	683	10	1.5 (0.7-2.8)	72.7	7.27	1.1	0.2-1.8

* Cases Pf-LDH-positive but HRP2-negative on both RDTs were considered potential candidates for pfhrp2/3 gene deletion and defined as ‘discordant.’
** Overall proportion of 'discordant RDT profile' infections with *pfhrp2* deletion calls by PCR.

Supplementary Table 4. MIP subtelomeric structural profiles by region for 367 samples with both MIP calls and PCR data.

	**Region**		
**MIP call**	Tigray (n=128)	Amhara (n=208)	Gambella (n=31)
pfhrp2+	**79**	**143**	**19**
chr8-P1	40 (51%)	84 (59%)	13 (68%)
chr8-P2	18 (23%)	27 (19%)	3 (16%)
chr8-P3	21 (27%)	32 (22%)	3 (16%)
pfhrp2-	**49**	**65**	**12**
chr8-P4	49 (100%)	65 (100%)	12 (100%)
pfhrp3+	**31**	**59**	**13**
chr13-P1	31 (100%)	59 (100%)	13 (100%)
pfhrp3-	**97**	**149**	**18**
chr13-P3	41 (42%)	70 (47%)	7 (39%)
chr13-P4	56 (58%)	79 (53%)	11 (61%)

Supplementary Table 5. Pfhrp2/3 MIP panel design overview, including genes targeted, MIPs designed, and genomic coordinates.
Key: Target Group: An identifier for the MIP target region. It is possible and preferred to design probes for multiple genes within a single target group if they are in close proximity; Gene ID: targeted gene's identifier; annotation: target gene annotation; MIP: MIP ID; Copy: Target copy ID for paralogous/multicopy genes; orientation: probe orientation relative to the reference genome; chrom: MIP target chromosome; capture_start: genomic coordinate of the start of captured sequence; capture_end: genomic coordinate of the end of captured sequence; gene_start: starting position of the target gene; gene_end: end position of the target gene; multicopy: whether the gene has a paralog on another location in the genome.
Genes for which MIP design failed are still retained in the table to provide an overview of the entire target region.
Alternating shading of rows are demarcating targeted genes.

Target Group	Gene ID	annotation	MIP	Copy	orientation	chrom	capture_start	capture_end	gene_start	gene_end	multicopy
PF3D7-0831400	PF3D7_0831400	Plasmodium exported protein, unknown function	PF3D7-0831400_S0_Sub0_	C0	forward	chr8	1344451	1344645	1344405	1345487	FALSE
PF3D7-0831400	PF3D7_0831400	Plasmodium exported protein, unknown function	PF3D7-0831400_S0_Sub0_	C0	reverse	chr8	1344498	1344701	1344405	1345487	FALSE
PF3D7-0831400	PF3D7_0831400	Plasmodium exported protein, unknown function	PF3D7-0831400_S0_Sub0_	C0	forward	chr8	1344701	1344906	1344405	1345487	FALSE
PF3D7-0831400	PF3D7_0831400	Plasmodium exported protein, unknown function	PF3D7-0831400_S0_Sub0_	C0	reverse	chr8	1344779	1344968	1344405	1345487	FALSE
PF3D7-0831400	PF3D7_0831400	Plasmodium exported protein, unknown function	PF3D7-0831400_S0_Sub0_	C0	forward	chr8	1344968	1345115	1344405	1345487	FALSE
PF3D7-0831400	PF3D7_0831400	Plasmodium exported protein, unknown function	PF3D7-0831400_S0_Sub0_	C0	reverse	chr8	1345114	1345327	1344405	1345487	FALSE
PF3D7-0831400	PF3D7_0831400	Plasmodium exported protein, unknown function	PF3D7-0831400_S0_Sub0_	C0	forward	chr8	1345238	1345442	1344405	1345487	FALSE
PF3D7-0831400	PF3D7_0831400	Plasmodium exported protein, unknown function	PF3D7-0831400_S0_Sub0_	C0	forward	chr8	1345510	1345689	1344405	1345487	FALSE
PF3D7-0831500	PF3D7_0831500	Plasmodium exported protein (PHIST), unknown functi	PF3D7-0831500_S0_Sub0_	C0	forward	chr8	1353428	1353627	1353456	1354669	FALSE
PF3D7-0831500	PF3D7_0831500	Plasmodium exported protein (PHIST), unknown functi	PF3D7-0831500_S0_Sub0_	C0	reverse	chr8	1353580	1353743	1353456	1354669	FALSE
PF3D7-0831500	PF3D7_0831500	Plasmodium exported protein (PHIST), unknown functi	PF3D7-0831500_S0_Sub0_	C0	forward	chr8	1353726	1353924	1353456	1354669	FALSE
PF3D7-0831500	PF3D7_0831500	Plasmodium exported protein (PHIST), unknown functi	PF3D7-0831500_S0_Sub0_	C0	reverse	chr8	1353887	1354058	1353456	1354669	FALSE
PF3D7-0831500	PF3D7_0831500	Plasmodium exported protein (PHIST), unknown functi	PF3D7-0831500_S0_Sub0_	C0	forward	chr8	1354006	1354194	1353456	1354669	FALSE
PF3D7-0831500	PF3D7_0831500	Plasmodium exported protein (PHIST), unknown functi	PF3D7-0831500_S0_Sub0_	C0	reverse	chr8	1354153	1354316	1353456	1354669	FALSE
PF3D7-0831500	PF3D7_0831500	Plasmodium exported protein (PHIST), unknown functi	PF3D7-0831500_S0_Sub0_	C0	forward	chr8	1354246	1354421	1353456	1354669	FALSE
PF3D7-0831500	PF3D7_0831500	Plasmodium exported protein (PHIST), unknown functi	PF3D7-0831500_S0_Sub0_	C0	reverse	chr8	1354391	1354562	1353456	1354669	FALSE
PF3D7-0831500	PF3D7_0831500	Plasmodium exported protein (PHIST), unknown functi	PF3D7-0831500_S0_Sub0_	C0	forward	chr8	1354535	1354733	1353456	1354669	FALSE
PF3D7-0831600	PF3D7_0831600	cytoadherence linked asexual protein 8	PF3D7-0831600_S0_Sub0_	C0	reverse	chr8	1358227	1358416	1358314	1363618	FALSE
PF3D7-0831600	PF3D7_0831600	cytoadherence linked asexual protein 8	PF3D7-0831600_S0_Sub0_	C0	reverse	chr8	1358417	1358620	1358314	1363618	FALSE
PF3D7-0831600	PF3D7_0831600	cytoadherence linked asexual protein 8	PF3D7-0831600_S0_Sub0_	C0	reverse	chr8	1358512	1358684	1358314	1363618	FALSE
PF3D7-0831600	PF3D7_0831600	cytoadherence linked asexual protein 8	PF3D7-0831600_S0_Sub0_	C0	forward	chr8	1358685	1358896	1358314	1363618	FALSE
PF3D7-0831600	PF3D7_0831600	cytoadherence linked asexual protein 8	PF3D7-0831600_S0_Sub0_	C0	forward	chr8	1358866	1359070	1358314	1363618	FALSE
PF3D7-0831600	PF3D7_0831600	cytoadherence linked asexual protein 8	PF3D7-0831600_S0_Sub0_	C0	reverse	chr8	1359316	1359495	1358314	1363618	FALSE
PF3D7-0831600	PF3D7_0831600	cytoadherence linked asexual protein 8	PF3D7-0831600_S0_Sub0_	C0	forward	chr8	1359452	1359625	1358314	1363618	FALSE
PF3D7-0831600	PF3D7_0831600	cytoadherence linked asexual protein 8	PF3D7-0831600_S0_Sub0_	C0	reverse	chr8	1359601	1359789	1358314	1363618	FALSE
PF3D7-0831600	PF3D7_0831600	cytoadherence linked asexual protein 8	PF3D7-0831600_S0_Sub0_	C0	reverse	chr8	1360022	1360201	1358314	1363618	FALSE
PF3D7-0831600	PF3D7_0831600	cytoadherence linked asexual protein 8	PF3D7-0831600_S0_Sub0_	C0	reverse	chr8	1360210	1360415	1358314	1363618	FALSE
PF3D7-0831600	PF3D7_0831600	cytoadherence linked asexual protein 8	PF3D7-0831600_S0_Sub0_	C0	reverse	chr8	1360426	1360635	1358314	1363618	FALSE
PF3D7-0831600	PF3D7_0831600	cytoadherence linked asexual protein 8	PF3D7-0831600_S0_Sub0_	C0	reverse	chr8	1360699	1360912	1358314	1363618	FALSE
PF3D7-0831600	PF3D7_0831600	cytoadherence linked asexual protein 8	PF3D7-0831600_S0_Sub0_	C0	reverse	chr8	1360950	1361117	1358314	1363618	FALSE
PF3D7-0831600	PF3D7_0831600	cytoadherence linked asexual protein 8	PF3D7-0831600_S0_Sub0_	C0	forward	chr8	1361101	1361299	1358314	1363618	FALSE
PF3D7-0831600	PF3D7_0831600	cytoadherence linked asexual protein 8	PF3D7-0831600_S0_Sub0_	C0	reverse	chr8	1361271	1361441	1358314	1363618	FALSE
PF3D7-0831600	PF3D7_0831600	cytoadherence linked asexual protein 8	PF3D7-0831600_S0_Sub0_	C0	reverse	chr8	1361516	1361685	1358314	1363618	FALSE
PF3D7-0831600	PF3D7_0831600	cytoadherence linked asexual protein 8	PF3D7-0831600_S0_Sub0_	C0	forward	chr8	1361679	1361888	1358314	1363618	FALSE
PF3D7-0831600	PF3D7_0831600	cytoadherence linked asexual protein 8	PF3D7-0831600_S0_Sub0_	C0	reverse	chr8	1361770	1361964	1358314	1363618	FALSE
PF3D7-0831600	PF3D7_0831600	cytoadherence linked asexual protein 8	PF3D7-0831600_S0_Sub0_	C0	reverse	chr8	1362282	1362464	1358314	1363618	FALSE
PF3D7-0831600	PF3D7_0831600	cytoadherence linked asexual protein 8	PF3D7-0831600_S0_Sub0_	C0	forward	chr8	1362452	1362651	1358314	1363618	FALSE
PF3D7-0831600	PF3D7_0831600	cytoadherence linked asexual protein 8	PF3D7-0831600_S0_Sub0_	C0	forward	chr8	1362686	1362852	1358314	1363618	FALSE
PF3D7-0831600	PF3D7_0831600	cytoadherence linked asexual protein 8	PF3D7-0831600_S0_Sub0_	C0	reverse	chr8	1362870	1363048	1358314	1363618	FALSE
PF3D7-0831600	PF3D7_0831600	cytoadherence linked asexual protein 8	PF3D7-0831600_S0_Sub0_	C0	forward	chr8	1362995	1363185	1358314	1363618	FALSE
PF3D7-0831600	PF3D7_0831600	cytoadherence linked asexual protein 8	PF3D7-0831600_S0_Sub0_	C0	forward	chr8	1363201	1363415	1358314	1363618	FALSE
PF3D7-0831600	PF3D7_0831600	cytoadherence linked asexual protein 8	PF3D7-0831600_S0_Sub0_	C0	forward	chr8	1363389	1363584	1358314	1363618	FALSE
PF3D7-0831700	PF3D7_0831700	heat shock protein 70	PF3D7-0831700_S0_Sub0_	C0	reverse	chr8	1365415	1365577	1365467	1367506	FALSE
PF3D7-0831700	PF3D7_0831700	heat shock protein 70	PF3D7-0831700_S0_Sub0_	C0	forward	chr8	1365517	1365690	1365467	1367506	FALSE
PF3D7-0831700	PF3D7_0831700	heat shock protein 70	PF3D7-0831700_S0_Sub0_	C0	reverse	chr8	1365668	1365855	1365467	1367506	FALSE
PF3D7-0831700	PF3D7_0831700	heat shock protein 70	PF3D7-0831700_S0_Sub0_	C0	forward	chr8	1365845	1366018	1365467	1367506	FALSE
PF3D7-0831700	PF3D7_0831700	heat shock protein 70	PF3D7-0831700_S0_Sub0_	C0	reverse	chr8	1365922	1366112	1365467	1367506	FALSE
PF3D7-0831700	PF3D7_0831700	heat shock protein 70	PF3D7-0831700_S0_Sub0_	C0	forward	chr8	1366111	1366306	1365467	1367506	FALSE
PF3D7-0831700	PF3D7_0831700	heat shock protein 70	PF3D7-0831700_S0_Sub0_	C0	reverse	chr8	1366184	1366356	1365467	1367506	FALSE
PF3D7-0831700	PF3D7_0831700	heat shock protein 70	PF3D7-0831700_S0_Sub0_	C0	forward	chr8	1366347	1366532	1365467	1367506	FALSE
PF3D7-0831700	PF3D7_0831700	heat shock protein 70	PF3D7-0831700_S0_Sub0_	C0	reverse	chr8	1366443	1366638	1365467	1367506	FALSE
PF3D7-0831700	PF3D7_0831700	heat shock protein 70	PF3D7-0831700_S0_Sub0_	C0	forward	chr8	1366597	1366801	1365467	1367506	FALSE
PF3D7-0831700	PF3D7_0831700	heat shock protein 70	PF3D7-0831700_S0_Sub0_	C0	reverse	chr8	1366771	1366959	1365467	1367506	FALSE
PF3D7-0831700	PF3D7_0831700	heat shock protein 70	PF3D7-0831700_S0_Sub0_	C0	forward	chr8	1366886	1367107	1365467	1367506	FALSE
PF3D7-0831700	PF3D7_0831700	heat shock protein 70	PF3D7-0831700_S0_Sub0_	C0	reverse	chr8	1367010	1367187	1365467	1367506	FALSE
PF3D7-0831700	PF3D7_0831700	heat shock protein 70	PF3D7-0831700_S0_Sub0_	C0	forward	chr8	1367148	1367355	1365467	1367506	FALSE
PF3D7-0831700	PF3D7_0831700	heat shock protein 70	PF3D7-0831700_S0_Sub0_	C0	reverse	chr8	1367243	1367422	1365467	1367506	FALSE
PF3D7-0831700	PF3D7_0831700	heat shock protein 70	PF3D7-0831700_S0_Sub0_	C0	forward	chr8	1367406	1367616	1365467	1367506	FALSE
PF3D7-0831750	PF3D7_0831750	Plasmodium exported protein (PHISTa), unknown func	PF3D7-0831750_S0_Sub0_	C0	forward	chr8	1371664	1371848	1371847	1372720	FALSE
PF3D7-0831750	PF3D7_0831750	Plasmodium exported protein (PHISTa), unknown func	PF3D7-0831750_S0_Sub0_	C0	reverse	chr8	1371800	1371998	1371847	1372720	FALSE
PF3D7-0831750	PF3D7_0831750	Plasmodium exported protein (PHISTa), unknown func	PF3D7-0831750_S0_Sub0_	C0	forward	chr8	1371969	1372175	1371847	1372720	FALSE
PF3D7-0831750	PF3D7_0831750	Plasmodium exported protein (PHISTa), unknown func	PF3D7-0831750_S0_Sub0_	C0	reverse	chr8	1372168	1372367	1371847	1372720	FALSE
PF3D7-0831750	PF3D7_0831750	Plasmodium exported protein (PHISTa), unknown func	PF3D7-0831750_S0_Sub0_	C0	forward	chr8	1372345	1372509	1371847	1372720	FALSE
PF3D7-0831750	PF3D7_0831750	Plasmodium exported protein (PHISTa), unknown func	PF3D7-0831750_S0_Sub0_	C0	reverse	chr8	1372430	1372615	1371847	1372720	FALSE
PF3D7-0831750	PF3D7_0831750	Plasmodium exported protein (PHISTa), unknown func	PF3D7-0831750_S0_Sub0_	C0	forward	chr8	1372614	1372807	1371847	1372720	FALSE
hrp2	PF3D7_0831800	histidine-rich protein II	hrp2_S0_Sub0_mip0	C0	reverse	chr8	1374269	1374450	1374236	1375299	FALSE
hrp2	PF3D7_0831800	histidine-rich protein II	hrp2_S0_Sub0_mip1	C0	forward	chr8	1374297	1374521	1374236	1375299	FALSE
hrp2	PF3D7_0831800	histidine-rich protein II	hrp2_S0_Sub0_mip2	C0	forward	chr8	1374297	1374498	1374236	1375299	FALSE
hrp2	PF3D7_0831800	histidine-rich protein II	hrp2_S0_Sub0_mip3	C0	reverse	chr8	1374430	1374625	1374236	1375299	FALSE
hrp2	PF3D7_0831800	histidine-rich protein II	hrp2_S0_Sub0_mip4	C0	reverse	chr8	1374468	1374661	1374236	1375299	FALSE
hrp2	PF3D7_0831800	histidine-rich protein II	hrp2_S0_Sub0_mip5	C0	reverse	chr8	1374619	1374805	1374236	1375299	FALSE
hrp2	PF3D7_0831800	histidine-rich protein II	hrp2_S0_Sub0_mip6	C0	forward	chr8	1374630	1374822	1374236	1375299	FALSE
hrp2	PF3D7_0831800	histidine-rich protein II	hrp2_S0_Sub0_mip7	C0	forward	chr8	1374763	1374985	1374236	1375299	FALSE
hrp2	PF3D7_0831800	histidine-rich protein II	hrp2_S0_Sub0_mip8	C0	reverse	chr8	1374871	1375047	1374236	1375299	FALSE
PF3D7-0831900	PF3D7_0831900	Plasmodium exported protein (PHIST), unknown functi	PF3D7-0831900_S0_Sub0_	C0	forward	chr8	1380756	1380927	1380834	1381835	FALSE
PF3D7-0831900	PF3D7_0831900	Plasmodium exported protein (PHIST), unknown functi	PF3D7-0831900_S0_Sub0_	C0	reverse	chr8	1380948	1381125	1380834	1381835	FALSE
PF3D7-0832000	PF3D7_0832000	stevor	PF3D7-0832000_S0_Sub0_	C0	forward	chr8	1383785	1383949	1383349	1384377	FALSE
PF3D7-0832000	PF3D7_0832000	stevor	PF3D7-0832000_S0_Sub0_	C0	reverse	chr8	1383870	1384053	1383349	1384377	FALSE
PF3D7-0832000	PF3D7_0832000	stevor	PF3D7-0832000_S0_Sub0_	C0	forward	chr8	1384048	1384225	1383349	1384377	FALSE
PF3D7-0832000	PF3D7_0832000	stevor	PF3D7-0832000_S0_Sub0_	C0	reverse	chr8	1384194	1384363	1383349	1384377	FALSE
PF3D7-0832000	PF3D7_0832000	stevor	PF3D7-0832000_S0_Sub0_	C0	forward	chr8	1384364	1384560	1383349	1384377	FALSE
PF3D7-0832000	PF3D7_0832000	stevor	PF3D7-0832000_S0_Sub0_	C0	reverse	chr8	1384390	1384585	1383349	1384377	FALSE
PF3D7-0832100	PF3D7_0832100	rifin, pseudogene	PF3D7-0832100_S0_Sub0_	C0	forward	chr8	1386570	1386754	1386625	1387882	FALSE
PF3D7-0832100	PF3D7_0832100	rifin, pseudogene	PF3D7-0832100_S0_Sub0_	C0	reverse	chr8	1386610	1386808	1386625	1387882	FALSE
PF3D7-0832100	PF3D7_0832100	rifin, pseudogene	PF3D7-0832100_S0_Sub0_	C0	forward	chr8	1386788	1386973	1386625	1387882	FALSE
PF3D7-0832100	PF3D7_0832100	rifin, pseudogene	PF3D7-0832100_S0_Sub0_	C0	reverse	chr8	1386959	1387131	1386625	1387882	FALSE
PF3D7-0832100	PF3D7_0832100	rifin, pseudogene	PF3D7-0832100_S0_Sub0_	C0	forward	chr8	1387025	1387224	1386625	1387882	FALSE
PF3D7-0832100	PF3D7_0832100	rifin, pseudogene	PF3D7-0832100_S0_Sub0_	C0	reverse	chr8	1387189	1387362	1386625	1387882	FALSE
PF3D7-0832100	PF3D7_0832100	rifin, pseudogene	PF3D7-0832100_S0_Sub0_	C0	forward	chr8	1387281	1387480	1386625	1387882	FALSE
PF3D7-0832100	PF3D7_0832100	rifin, pseudogene	PF3D7-0832100_S0_Sub0_	C0	reverse	chr8	1387448	1387625	1386625	1387882	FALSE
PF3D7-0832200-2	PF3D7_0832200	Plasmodium exported protein (PHISTa-like), unknown	PF3D7-0832200-2_S0_Sub0	C0	reverse	chr8	1392258	1392431	1392168	1393491	FALSE
PF3D7-0832200-2	PF3D7_0832200	Plasmodium exported protein (PHISTa-like), unknown	PF3D7-0832200-2_S0_Sub0	C0	forward	chr8	1392429	1392612	1392168	1393491	FALSE
PF3D7-0832200-2	PF3D7_0832200	Plasmodium exported protein (PHISTa-like), unknown	PF3D7-0832200-2_S0_Sub0	C0	forward	chr8	1392665	1392854	1392168	1393491	FALSE
PF3D7-0832200-2	PF3D7_0832200	Plasmodium exported protein (PHISTa-like), unknown	PF3D7-0832200-2_S0_Sub0	C0	forward	chr8	1392878	1393071	1392168	1393491	FALSE
PF3D7-0832200-2	PF3D7_0832200	Plasmodium exported protein (PHISTa-like), unknown	PF3D7-0832200-2_S0_Sub0	C0	reverse	chr8	1392909	1393108	1392168	1393491	FALSE
PF3D7-0832300	PF3D7_0832300	Plasmodium exported protein (PHISTa-like), unknown	PF3D7-0832300_S0_Sub0_	C0	forward	chr8	1397685	1397861	1397014	1397885	FALSE
PF3D7-0832500	PF3D7_0832500	rifin	PF3D7-0832500_S0_Sub0_	C0	forward	chr8	1402717	1402904	1402567	1403711	FALSE
PF3D7-0832400	PF3D7_0832400	stevor,PIR protein				chr8			1399559	1400583	FALSE
PF3D7-0832600	PF3D7_0832600	stevor,PIR protein				chr8			1405834	1406999	FALSE
PF3D7-0832700	PF3D7_0832700	Plasmodium exported protein (PHISTa-like), unknown				chr8			1408570	1409356	FALSE
PF3D7-0832800	PF3D7_0832800	rifin,PIR protein (RIF)				chr8			1411057	1412141	FALSE
PF3D7-0832900	PF3D7_0832900	stevor,PIR protein				chr8			1414282	1415293	FALSE
PF3D7-0833000	PF3D7_0833000	rifin,PIR protein (RIF)				chr8			1417437	1418625	FALSE
PF3D7-0833100	PF3D7_0833100	rifin,PIR protein (RIF)				chr8			1420817	1422077	FALSE
PF3D7-0833200	PF3D7_0833200	rifin,PIR protein (RIF)				chr8			1426883	1428135	FALSE
PF3D7-0833300	PF3D7_0833300	erythrocyte membrane protein 1 (PfEMP1), exon 2, ps				chr8			1429329	1430514	FALSE
PF3D7-0833400	PF3D7_0833400	rifin,PIR protein (RIF)				chr8			1432695	1433932	FALSE
PF3D7-0833500	PF3D7_0833500	erythrocyte membrane protein 1, PfEMP1 (VAR)				chr8			1435793	1443449	FALSE
PF3D7-1369900	PF3D7_1369900	tRNA Tryptophan	PF3D7-1369900_S0_Sub0_	C0	reverse	chr13	2780863	2781067	2781080	2781151	FALSE
PF3D7-1369900	PF3D7_1369900	tRNA Tryptophan	PF3D7-1369900_S0_Sub0_	C0	forward	chr13	2780963	2781160	2781080	2781151	FALSE
PF3D7-1369900	PF3D7_1369900	tRNA Tryptophan	PF3D7-1369900_S0_Sub0_	C0	reverse	chr13	2781106	2781280	2781080	2781151	FALSE
PF3D7-1369900	PF3D7_1370000	tRNA Arginine	PF3D7-1369900_S0_Sub0_	C0	reverse	chr13	2781638	2781839	2781622	2781695	FALSE
PF3D7-1370100	PF3D7_1370100	tRNA Cysteine	PF3D7-1370100_S0_Sub0_	C0	forward	chr13	2783006	2783199	2783150	2783221	FALSE
PF3D7-1370100	PF3D7_1370100	tRNA Cysteine	PF3D7-1370100_S0_Sub0_	C0	reverse	chr13	2783188	2783408	2783150	2783221	FALSE
PF3D7-1370200	PF3D7_1370200	tRNA Glycine	PF3D7-1370200_S0_Sub0_	C0	forward	chr13	2783580	2783800	2783766	2783836	FALSE
PF3D7-1370200	PF3D7_1370200	tRNA Glycine	PF3D7-1370200_S0_Sub0_	C0	reverse	chr13	2783801	2783989	2783766	2783836	FALSE
PF3D7-1370300	PF3D7_1370300	membrane associated histidine-rich protein 1	PF3D7-1370300_S0_Sub0_	C0	reverse	chr13	2787987	2788186	2787972	2788913	FALSE
PF3D7-1370300	PF3D7_1370300	membrane associated histidine-rich protein 1	PF3D7-1370300_S0_Sub0_	C0	forward	chr13	2788160	2788346	2787972	2788913	FALSE
PF3D7-1370300	PF3D7_1370300	membrane associated histidine-rich protein 1	PF3D7-1370300_S0_Sub0_	C0	reverse	chr13	2788281	2788487	2787972	2788913	FALSE
PF3D7-1370300	PF3D7_1370300	membrane associated histidine-rich protein 1	PF3D7-1370300_S0_Sub0_	C0	forward	chr13	2788486	2788674	2787972	2788913	FALSE
PF3D7-1370300	PF3D7_1370300	membrane associated histidine-rich protein 1	PF3D7-1370300_S0_Sub0_	C0	reverse	chr13	2788646	2788819	2787972	2788913	FALSE
PF3D7-1370300	PF3D7_1370300	membrane associated histidine-rich protein 1	PF3D7-1370300_S0_Sub0_	C0	forward	chr13	2788740	2788907	2787972	2788913	FALSE
PF3D7-1370500	PF3D7_1370500	unspecified product	PF3D7-1370500_S0_Sub0_	C0	reverse	chr13	2794273	2794464	2794237	2794851	TRUE
PF3D7-1370500	PF3D7_1370500	unspecified product	PF3D7-1370500_S0_Sub0_	C0	forward	chr13	2794388	2794592	2794237	2794851	TRUE
PF3D7-1370500	PF3D7_1370500	unspecified product	PF3D7-1370500_S0_Sub0_	C0	reverse	chr13	2794590	2794763	2794237	2794851	TRUE
PF3D7-1370500	PF3D7_1370500	unspecified product	PF3D7-1370500_S0_Sub0_	C0	forward	chr13	2794718	2794887	2794237	2794851	TRUE
PF3D7-1370500	PF3D7_1370500	unspecified product	PF3D7-1370500_S0_Sub0_	C0	reverse	chr13	2794812	2795027	2794237	2794851	TRUE
PF3D7-1370900	PF3D7_1370900	unspecified product	PF3D7-1370900_S0_Sub0_	C0	forward	chr13	2797288	2797472	2797507	2798103	TRUE
PF3D7-1370900	PF3D7_1370900	unspecified product	PF3D7-1370900_S0_Sub0_	C0	reverse	chr13	2797384	2797578	2797507	2798103	TRUE
PF3D7-1370900	PF3D7_1370900	unspecified product	PF3D7-1370900_S0_Sub0_	C0	forward	chr13	2797866	2798036	2797507	2798103	TRUE
PF3D7-1370900	PF3D7_1370900	unspecified product	PF3D7-1370900_S0_Sub0_	C0	reverse	chr13	2798027	2798194	2797507	2798103	TRUE
PF3D7-1371000	PF3D7_1371200	5.8S ribosomal RNA	PF3D7-1371000_S0_Sub0_	C0	reverse	chr13	2802545	2802759	2802527	2802686	TRUE
PF3D7-1371000	PF3D7_1371200	5.8S ribosomal RNA	PF3D7-1371000_S0_Sub0_	C0	reverse	chr13	2802681	2802868	2802527	2802686	TRUE
PF3D7-1371000	PF3D7_1371300	28S ribosomal RNA	PF3D7-1371000_S0_Sub0_	C0	reverse	chr13	2802887	2803071	2802945	2807159	TRUE
PF3D7-1371000	PF3D7_1371300	28S ribosomal RNA	PF3D7-1371000_S0_Sub0_	C0	reverse	chr13	2802890	2803101	2802945	2807159	TRUE
PF3D7-1371000	PF3D7_1371300	28S ribosomal RNA	PF3D7-1371000_S0_Sub0_	C0	forward	chr13	2803077	2803279	2802945	2807159	TRUE
PF3D7-1371000	PF3D7_1371300	28S ribosomal RNA	PF3D7-1371000_S0_Sub0_	C0	reverse	chr13	2803230	2803425	2802945	2807159	TRUE
PF3D7-1371000	PF3D7_1371300	28S ribosomal RNA	PF3D7-1371000_S0_Sub0_	C0	forward	chr13	2803404	2803620	2802945	2807159	TRUE
PF3D7-1371000	PF3D7_1371300	28S ribosomal RNA	PF3D7-1371000_S0_Sub0_	C0	reverse	chr13	2803604	2803824	2802945	2807159	TRUE
PF3D7-1371000	PF3D7_1371300	28S ribosomal RNA	PF3D7-1371000_S0_Sub0_	C0	forward	chr13	2803821	2804038	2802945	2807159	TRUE
PF3D7-1371000	PF3D7_1371300	28S ribosomal RNA	PF3D7-1371000_S0_Sub0_	C0	reverse	chr13	2803985	2804201	2802945	2807159	TRUE
PF3D7-1371000	PF3D7_1371300	28S ribosomal RNA	PF3D7-1371000_S0_Sub0_	C0	forward	chr13	2804128	2804329	2802945	2807159	TRUE
PF3D7-1371000	PF3D7_1371300	28S ribosomal RNA	PF3D7-1371000_S0_Sub0_	C0	forward	chr13	2804236	2804420	2802945	2807159	TRUE
PF3D7-1371000	PF3D7_1371300	28S ribosomal RNA	PF3D7-1371000_S0_Sub0_	C0	forward	chr13	2804453	2804665	2802945	2807159	TRUE
PF3D7-1371000	PF3D7_1371300	28S ribosomal RNA	PF3D7-1371000_S0_Sub0_	C0	forward	chr13	2804733	2804952	2802945	2807159	TRUE
PF3D7-1371000	PF3D7_1371300	28S ribosomal RNA	PF3D7-1371000_S0_Sub0_	C0	reverse	chr13	2804887	2805088	2802945	2807159	TRUE
PF3D7-1371000	PF3D7_1371300	28S ribosomal RNA	PF3D7-1371000_S0_Sub0_	C0	reverse	chr13	2805067	2805275	2802945	2807159	TRUE
PF3D7-1371000	PF3D7_1371300	28S ribosomal RNA	PF3D7-1371000_S0_Sub0_	C0	reverse	chr13	2805291	2805504	2802945	2807159	TRUE
PF3D7-1371000	PF3D7_1371300	28S ribosomal RNA	PF3D7-1371000_S0_Sub0_	C0	reverse	chr13	2805378	2805597	2802945	2807159	TRUE
PF3D7-1371000	PF3D7_1371300	28S ribosomal RNA	PF3D7-1371000_S0_Sub0_	C0	forward	chr13	2805781	2805997	2802945	2807159	TRUE
PF3D7-1371000	PF3D7_1371300	28S ribosomal RNA	PF3D7-1371000_S0_Sub0_	C0	forward	chr13	2806111	2806300	2802945	2807159	TRUE
PF3D7-1371000	PF3D7_1371300	28S ribosomal RNA	PF3D7-1371000_S0_Sub0_	C0	forward	chr13	2806117	2806319	2802945	2807159	TRUE
PF3D7-1371000	PF3D7_1371300	28S ribosomal RNA	PF3D7-1371000_S0_Sub0_	C0	forward	chr13	2806228	2806430	2802945	2807159	TRUE
PF3D7-1371000	PF3D7_1371300	28S ribosomal RNA	PF3D7-1371000_S0_Sub0_	C0	forward	chr13	2806917	2807127	2802945	2807159	TRUE
PF3D7-1371500	PF3D7_1371500	probable protein, unknown function	PF3D7-1371500_S0_Sub0_	C0	forward	chr13	2808585	2808762	2808563	2809222	FALSE
PF3D7-1371500	PF3D7_1371500	probable protein, unknown function	PF3D7-1371500_S0_Sub0_	C0	reverse	chr13	2808671	2808855	2808563	2809222	FALSE
PF3D7-1371500	PF3D7_1371500	probable protein, unknown function	PF3D7-1371500_S0_Sub0_	C0	forward	chr13	2808809	2808983	2808563	2809222	FALSE
PF3D7-1371500	PF3D7_1371500	probable protein, unknown function	PF3D7-1371500_S0_Sub0_	C0	reverse	chr13	2808964	2809134	2808563	2809222	FALSE
PF3D7-1371500	PF3D7_1371500	probable protein, unknown function	PF3D7-1371500_S0_Sub0_	C0	forward	chr13	2809086	2809265	2808563	2809222	FALSE
PF3D7-1371500	PF3D7_1371500	probable protein, unknown function	PF3D7-1371500_S0_Sub0_	C0	reverse	chr13	2809250	2809438	2808563	2809222	FALSE
PF3D7-1371600	PF3D7_1371600	erythrocyte binding like protein 1, pseudogene	PF3D7-1371600_S0_Sub0_	C0	forward	chr13	2811767	2811979	2811706	2820270	FALSE
PF3D7-1371600	PF3D7_1371600	erythrocyte binding like protein 1, pseudogene	PF3D7-1371600_S0_Sub0_	C0	forward	chr13	2811977	2812173	2811706	2820270	FALSE
PF3D7-1371600	PF3D7_1371600	erythrocyte binding like protein 1, pseudogene	PF3D7-1371600_S0_Sub0_	C0	forward	chr13	2812266	2812441	2811706	2820270	FALSE
PF3D7-1371600	PF3D7_1371600	erythrocyte binding like protein 1, pseudogene	PF3D7-1371600_S0_Sub0_	C0	reverse	chr13	2812420	2812618	2811706	2820270	FALSE
PF3D7-1371600	PF3D7_1371600	erythrocyte binding like protein 1, pseudogene	PF3D7-1371600_S0_Sub0_	C0	reverse	chr13	2812590	2812799	2811706	2820270	FALSE
PF3D7-1371600	PF3D7_1371600	erythrocyte binding like protein 1, pseudogene	PF3D7-1371600_S0_Sub0_	C0	reverse	chr13	2812837	2813043	2811706	2820270	FALSE
PF3D7-1371600	PF3D7_1371600	erythrocyte binding like protein 1, pseudogene	PF3D7-1371600_S0_Sub0_	C0	forward	chr13	2813009	2813224	2811706	2820270	FALSE
PF3D7-1371600	PF3D7_1371600	erythrocyte binding like protein 1, pseudogene	PF3D7-1371600_S0_Sub0_	C0	forward	chr13	2813244	2813443	2811706	2820270	FALSE
PF3D7-1371600	PF3D7_1371600	erythrocyte binding like protein 1, pseudogene	PF3D7-1371600_S0_Sub0_	C0	reverse	chr13	2813421	2813614	2811706	2820270	FALSE
PF3D7-1371600	PF3D7_1371600	erythrocyte binding like protein 1, pseudogene	PF3D7-1371600_S0_Sub0_	C0	reverse	chr13	2814557	2814756	2811706	2820270	FALSE
PF3D7-1371600	PF3D7_1371600	erythrocyte binding like protein 1, pseudogene	PF3D7-1371600_S0_Sub0_	C0	reverse	chr13	2814849	2815025	2811706	2820270	FALSE
PF3D7-1371600	PF3D7_1371600	erythrocyte binding like protein 1, pseudogene	PF3D7-1371600_S0_Sub0_	C0	reverse	chr13	2815114	2815330	2811706	2820270	FALSE
PF3D7-1371600	PF3D7_1371600	erythrocyte binding like protein 1, pseudogene	PF3D7-1371600_S0_Sub0_	C0	reverse	chr13	2815719	2815912	2811706	2820270	FALSE
PF3D7-1371600	PF3D7_1371600	erythrocyte binding like protein 1, pseudogene	PF3D7-1371600_S0_Sub0_	C0	reverse	chr13	2816090	2816295	2811706	2820270	FALSE
PF3D7-1371600	PF3D7_1371600	erythrocyte binding like protein 1, pseudogene	PF3D7-1371600_S0_Sub0_	C0	reverse	chr13	2816383	2816585	2811706	2820270	FALSE
PF3D7-1371600	PF3D7_1371600	erythrocyte binding like protein 1, pseudogene	PF3D7-1371600_S0_Sub0_	C0	forward	chr13	2817675	2817894	2811706	2820270	FALSE
PF3D7-1371600	PF3D7_1371600	erythrocyte binding like protein 1, pseudogene	PF3D7-1371600_S0_Sub0_	C0	reverse	chr13	2817881	2818091	2811706	2820270	FALSE
PF3D7-1371600	PF3D7_1371600	erythrocyte binding like protein 1, pseudogene	PF3D7-1371600_S0_Sub0_	C0	forward	chr13	2818001	2818208	2811706	2820270	FALSE
PF3D7-1371600	PF3D7_1371600	erythrocyte binding like protein 1, pseudogene	PF3D7-1371600_S0_Sub0_	C0	reverse	chr13	2818179	2818356	2811706	2820270	FALSE
PF3D7-1371600	PF3D7_1371600	erythrocyte binding like protein 1, pseudogene	PF3D7-1371600_S0_Sub0_	C0	forward	chr13	2818278	2818498	2811706	2820270	FALSE
PF3D7-1371600	PF3D7_1371600	erythrocyte binding like protein 1, pseudogene	PF3D7-1371600_S0_Sub0_	C0	forward	chr13	2818563	2818774	2811706	2820270	FALSE
PF3D7-1371600	PF3D7_1371600	erythrocyte binding like protein 1, pseudogene	PF3D7-1371600_S0_Sub0_	C0	forward	chr13	2818861	2819039	2811706	2820270	FALSE
PF3D7-1371700	PF3D7_1371700	serine/threonine protein kinase, FIKK family	PF3D7-1371700_S0_Sub0_	C0	forward	chr13	2820938	2821144	2821078	2823292	FALSE
PF3D7-1371700	PF3D7_1371700	serine/threonine protein kinase, FIKK family	PF3D7-1371700_S0_Sub0_	C0	reverse	chr13	2821099	2821304	2821078	2823292	FALSE
PF3D7-1371700	PF3D7_1371700	serine/threonine protein kinase, FIKK family	PF3D7-1371700_S0_Sub0_	C0	forward	chr13	2821300	2821493	2821078	2823292	FALSE
PF3D7-1371700	PF3D7_1371700	serine/threonine protein kinase, FIKK family	PF3D7-1371700_S0_Sub0_	C0	reverse	chr13	2821460	2821668	2821078	2823292	FALSE
PF3D7-1371700	PF3D7_1371700	serine/threonine protein kinase, FIKK family	PF3D7-1371700_S0_Sub0_	C0	forward	chr13	2821662	2821870	2821078	2823292	FALSE
PF3D7-1371700	PF3D7_1371700	serine/threonine protein kinase, FIKK family	PF3D7-1371700_S0_Sub0_	C0	reverse	chr13	2821820	2822011	2821078	2823292	FALSE
PF3D7-1371700	PF3D7_1371700	serine/threonine protein kinase, FIKK family	PF3D7-1371700_S0_Sub0_	C0	forward	chr13	2822011	2822216	2821078	2823292	FALSE
PF3D7-1371700	PF3D7_1371700	serine/threonine protein kinase, FIKK family	PF3D7-1371700_S0_Sub0_	C0	reverse	chr13	2822205	2822373	2821078	2823292	FALSE
PF3D7-1371700	PF3D7_1371700	serine/threonine protein kinase, FIKK family	PF3D7-1371700_S0_Sub0_	C0	forward	chr13	2822308	2822491	2821078	2823292	FALSE
PF3D7-1371700	PF3D7_1371700	serine/threonine protein kinase, FIKK family	PF3D7-1371700_S0_Sub0_	C0	reverse	chr13	2822469	2822659	2821078	2823292	FALSE
PF3D7-1371700	PF3D7_1371700	serine/threonine protein kinase, FIKK family	PF3D7-1371700_S0_Sub0_	C0	forward	chr13	2822584	2822761	2821078	2823292	FALSE
PF3D7-1371700	PF3D7_1371700	serine/threonine protein kinase, FIKK family	PF3D7-1371700_S0_Sub0_	C0	forward	chr13	2823252	2823451	2821078	2823292	FALSE
PF3D7-1371800	PF3D7_1371800	Plasmodium exported protein, unknown function	PF3D7-1371800_S0_Sub0_	C0	forward	chr13	2824238	2824413	2824302	2825852	FALSE
PF3D7-1371800	PF3D7_1371800	Plasmodium exported protein, unknown function	PF3D7-1371800_S0_Sub0_	C0	reverse	chr13	2824387	2824571	2824302	2825852	FALSE
PF3D7-1371800	PF3D7_1371800	Plasmodium exported protein, unknown function	PF3D7-1371800_S0_Sub0_	C0	forward	chr13	2824557	2824756	2824302	2825852	FALSE
PF3D7-1371800	PF3D7_1371800	Plasmodium exported protein, unknown function	PF3D7-1371800_S0_Sub0_	C0	reverse	chr13	2824720	2824924	2824302	2825852	FALSE
PF3D7-1371800	PF3D7_1371800	Plasmodium exported protein, unknown function	PF3D7-1371800_S0_Sub0_	C0	forward	chr13	2824862	2825065	2824302	2825852	FALSE
PF3D7-1371800	PF3D7_1371800	Plasmodium exported protein, unknown function	PF3D7-1371800_S0_Sub0_	C0	reverse	chr13	2825044	2825213	2824302	2825852	FALSE
PF3D7-1371800	PF3D7_1371800	Plasmodium exported protein, unknown function	PF3D7-1371800_S0_Sub0_	C0	forward	chr13	2825212	2825411	2824302	2825852	FALSE
PF3D7-1371800	PF3D7_1371800	Plasmodium exported protein, unknown function	PF3D7-1371800_S0_Sub0_	C0	reverse	chr13	2825286	2825480	2824302	2825852	FALSE
PF3D7-1371800	PF3D7_1371800	Plasmodium exported protein, unknown function	PF3D7-1371800_S0_Sub0_	C0	reverse	chr13	2825484	2825680	2824302	2825852	FALSE
PF3D7-1371800	PF3D7_1371800	Plasmodium exported protein, unknown function	PF3D7-1371800_S0_Sub0_	C0	forward	chr13	2825578	2825774	2824302	2825852	FALSE
PF3D7-1371800	PF3D7_1371800	Plasmodium exported protein, unknown function	PF3D7-1371800_S0_Sub0_	C0	reverse	chr13	2825791	2825996	2824302	2825852	FALSE
PF3D7-1371900	PF3D7_1371900	Plasmodium exported protein, unknown function	PF3D7-1371900_S0_Sub0_	C0	forward	chr13	2829900	2830069	2829856	2830669	FALSE
PF3D7-1371900	PF3D7_1371900	Plasmodium exported protein, unknown function	PF3D7-1371900_S0_Sub0_	C0	reverse	chr13	2829964	2830171	2829856	2830669	FALSE
PF3D7-1371900	PF3D7_1371900	Plasmodium exported protein, unknown function	PF3D7-1371900_S0_Sub0_	C0	forward	chr13	2830165	2830368	2829856	2830669	FALSE
PF3D7-1371900	PF3D7_1371900	Plasmodium exported protein, unknown function	PF3D7-1371900_S0_Sub0_	C0	reverse	chr13	2830343	2830520	2829856	2830669	FALSE
PF3D7-1371900	PF3D7_1371900	Plasmodium exported protein, unknown function	PF3D7-1371900_S0_Sub0_	C0	forward	chr13	2830434	2830643	2829856	2830669	FALSE
PF3D7-1371900	PF3D7_1371900	Plasmodium exported protein, unknown function	PF3D7-1371900_S0_Sub0_	C0	reverse	chr13	2830641	2830843	2829856	2830669	FALSE
PF3D7-1372000	PF3D7_1372000	Plasmodium exported protein (PHISTa), unknown func	PF3D7-1372000_S0_Sub0_	C0	forward	chr13	2832906	2833066	2832952	2834322	FALSE
PF3D7-1372000	PF3D7_1372000	Plasmodium exported protein (PHISTa), unknown func	PF3D7-1372000_S0_Sub0_	C0	reverse	chr13	2833052	2833251	2832952	2834322	FALSE
PF3D7-1372000	PF3D7_1372000	Plasmodium exported protein (PHISTa), unknown func	PF3D7-1372000_S0_Sub0_	C0	forward	chr13	2833241	2833442	2832952	2834322	FALSE
PF3D7-1372000	PF3D7_1372000	Plasmodium exported protein (PHISTa), unknown func	PF3D7-1372000_S0_Sub0_	C0	reverse	chr13	2833330	2833507	2832952	2834322	FALSE
PF3D7-1372000	PF3D7_1372000	Plasmodium exported protein (PHISTa), unknown func	PF3D7-1372000_S0_Sub0_	C0	forward	chr13	2833491	2833682	2832952	2834322	FALSE
PF3D7-1372000	PF3D7_1372000	Plasmodium exported protein (PHISTa), unknown func	PF3D7-1372000_S0_Sub0_	C0	reverse	chr13	2833575	2833782	2832952	2834322	FALSE
PF3D7-1372000	PF3D7_1372000	Plasmodium exported protein (PHISTa), unknown func	PF3D7-1372000_S0_Sub0_	C0	forward	chr13	2833764	2833939	2832952	2834322	FALSE
PF3D7-1372000	PF3D7_1372000	Plasmodium exported protein (PHISTa), unknown func	PF3D7-1372000_S0_Sub0_	C0	reverse	chr13	2833883	2834055	2832952	2834322	FALSE
PF3D7-1372000	PF3D7_1372000	Plasmodium exported protein (PHISTa), unknown func	PF3D7-1372000_S0_Sub0_	C0	reverse	chr13	2834183	2834346	2832952	2834322	FALSE
PF3D7-1372000	PF3D7_1372000	Plasmodium exported protein (PHISTa), unknown func	PF3D7-1372000_S0_Sub0_	C0	forward	chr13	2834232	2834431	2832952	2834322	FALSE
PF3D7-1372100	PF3D7_1372100	Plasmodium exported protein (PHISTb), unknown func	PF3D7-1372100_S0_Sub0_	C0	forward	chr13	2836945	2837114	2837053	2839058	FALSE
PF3D7-1372100	PF3D7_1372100	Plasmodium exported protein (PHISTb), unknown func	PF3D7-1372100_S0_Sub0_	C0	reverse	chr13	2837011	2837176	2837053	2839058	FALSE
PF3D7-1372100	PF3D7_1372100	Plasmodium exported protein (PHISTb), unknown func	PF3D7-1372100_S0_Sub0_	C0	forward	chr13	2837149	2837347	2837053	2839058	FALSE
PF3D7-1372100	PF3D7_1372100	Plasmodium exported protein (PHISTb), unknown func	PF3D7-1372100_S0_Sub0_	C0	reverse	chr13	2837337	2837545	2837053	2839058	FALSE
PF3D7-1372100	PF3D7_1372100	Plasmodium exported protein (PHISTb), unknown func	PF3D7-1372100_S0_Sub0_	C0	forward	chr13	2837460	2837631	2837053	2839058	FALSE
PF3D7-1372100	PF3D7_1372100	Plasmodium exported protein (PHISTb), unknown func	PF3D7-1372100_S0_Sub0_	C0	reverse	chr13	2837604	2837816	2837053	2839058	FALSE
PF3D7-1372100	PF3D7_1372100	Plasmodium exported protein (PHISTb), unknown func	PF3D7-1372100_S0_Sub0_	C0	forward	chr13	2837734	2837933	2837053	2839058	FALSE
PF3D7-1372100	PF3D7_1372100	Plasmodium exported protein (PHISTb), unknown func	PF3D7-1372100_S0_Sub0_	C0	reverse	chr13	2837874	2838075	2837053	2839058	FALSE
PF3D7-1372100	PF3D7_1372100	Plasmodium exported protein (PHISTb), unknown func	PF3D7-1372100_S0_Sub0_	C0	forward	chr13	2838030	2838197	2837053	2839058	FALSE
PF3D7-1372100	PF3D7_1372100	Plasmodium exported protein (PHISTb), unknown func	PF3D7-1372100_S0_Sub0_	C0	reverse	chr13	2838146	2838330	2837053	2839058	FALSE
PF3D7-1372100	PF3D7_1372100	Plasmodium exported protein (PHISTb), unknown func	PF3D7-1372100_S0_Sub0_	C0	forward	chr13	2838293	2838492	2837053	2839058	FALSE
PF3D7-1372100	PF3D7_1372100	Plasmodium exported protein (PHISTb), unknown func	PF3D7-1372100_S0_Sub0_	C0	reverse	chr13	2838437	2838650	2837053	2839058	FALSE
PF3D7-1372100	PF3D7_1372100	Plasmodium exported protein (PHISTb), unknown func	PF3D7-1372100_S0_Sub0_	C0	forward	chr13	2838545	2838757	2837053	2839058	FALSE
PF3D7-1372100	PF3D7_1372100	Plasmodium exported protein (PHISTb), unknown func	PF3D7-1372100_S0_Sub0_	C0	reverse	chr13	2838704	2838871	2837053	2839058	FALSE
PF3D7-1372100	PF3D7_1372100	Plasmodium exported protein (PHISTb), unknown func	PF3D7-1372100_S0_Sub0_	C0	forward	chr13	2838819	2839006	2837053	2839058	FALSE
PF3D7-1372100	PF3D7_1372100	Plasmodium exported protein (PHISTb), unknown func	PF3D7-1372100_S0_Sub0_	C0	reverse	chr13	2838998	2839199	2837053	2839058	FALSE
hrp3	PF3D7_1372200	histidine-rich protein III	hrp3_S0_Sub0_mip0	C0	forward	chr13	2840643	2840859	2840727	2841703	FALSE
hrp3	PF3D7_1372200	histidine-rich protein III	hrp3_S0_Sub0_mip2	C0	forward	chr13	2840751	2840954	2840727	2841703	FALSE
hrp3	PF3D7_1372200	histidine-rich protein III	hrp3_S0_Sub0_mip3	C0	reverse	chr13	2840794	2841015	2840727	2841703	FALSE
hrp3	PF3D7_1372200	histidine-rich protein III	hrp3_S0_Sub0_mip4	C0	forward	chr13	2841009	2841205	2840727	2841703	FALSE
hrp3	PF3D7_1372200	histidine-rich protein III	hrp3_S0_Sub0_mip5	C0	reverse	chr13	2841140	2841341	2840727	2841703	FALSE
hrp3	PF3D7_1372200	histidine-rich protein III	hrp3_S0_Sub0_mip6	C0	forward	chr13	2841246	2841436	2840727	2841703	FALSE
hrp3	PF3D7_1372200	histidine-rich protein III	hrp3_S0_Sub0_mip7	C0	reverse	chr13	2841420	2841635	2840727	2841703	FALSE
hrp3	PF3D7_1372200	histidine-rich protein III	hrp3_S0_Sub0_mip9	C0	forward	chr13	2841464	2841653	2840727	2841703	FALSE
hrp3	PF3D7_1372200	histidine-rich protein III	hrp3_S0_Sub0_mip10	C0	reverse	chr13	2841663	2841862	2840727	2841703	FALSE
PF3D7-1372400	PF3D7_1372400	acyl-CoA synthetase	PF3D7-1372400_S0_Sub0_	C0	forward	chr13	2852540	2852728	2850891	2853482	FALSE
PF3D7-1372400	PF3D7_1372400	acyl-CoA synthetase	PF3D7-1372400_S0_Sub0_	C0	reverse	chr13	2852715	2852915	2850891	2853482	FALSE
PF3D7-1372400	PF3D7_1372400	acyl-CoA synthetase	PF3D7-1372400_S0_Sub0_	C0	forward	chr13	2852916	2853119	2850891	2853482	FALSE
PF3D7-1372400	PF3D7_1372400	acyl-CoA synthetase	PF3D7-1372400_S0_Sub0_	C0	reverse	chr13	2853114	2853312	2850891	2853482	FALSE
PF3D7-1372400	PF3D7_1372400	acyl-CoA synthetase	PF3D7-1372400_S0_Sub0_	C0	forward	chr13	2853213	2853379	2850891	2853482	FALSE
PF3D7-1372400	PF3D7_1372400	acyl-CoA synthetase	PF3D7-1372400_S0_Sub0_	C0	reverse	chr13	2853355	2853533	2850891	2853482	FALSE
PF3D7-1372300	PF3D7_1372300	Plasmodium exported protein (PHIST), unknown functi				chr13			2845766	2846538	FALSE
PF3D7-1372500	PF3D7_1372500	stevor, pseudogene,PIR protein, pseudogene				chr13			2854543	2855568	FALSE
PF3D7-1372600	PF3D7_1372600	rifin,PIR protein (RIF)				chr13			2856585	2858026	FALSE
PF3D7-1372700	PF3D7_1372700	rifin,PIR protein (RIF)				chr13			2860162	2861441	FALSE
PF3D7-1372800	PF3D7_1372800	stevor,PIR protein				chr13			2863865	2864869	FALSE
PF3D7-1372900	PF3D7_1372900	erythrocyte membrane protein 1 (PfEMP1), exon 2, ps				chr13			2865892	2867226	FALSE
PF3D7-1373000	PF3D7_1373000	rifin,PIR protein (RIF)				chr13			2868922	2870165	FALSE
PF3D7-1373100	PF3D7_1373100	rifin,PIR protein (RIF)				chr13			2871859	2873088	FALSE
PF3D7-1373200	PF3D7_1373200	Plasmodium exported protein, unknown function, fragr				chr13			2875990	2876203	FALSE
PF3D7-1373300	PF3D7_1373300	rifin,PIR protein (RIF)				chr13			2877907	2879141	FALSE
PF3D7-1373400	PF3D7_1373400	rifin,PIR protein (RIF)				chr13			2881590	2882956	FALSE
PF3D7-1373500	PF3D7_1373500	erythrocyte membrane protein 1, PfEMP1 (VAR)				chr13			2884785	2892340	FALSE
PF3D7-1370500	PF3D7_1148200	unspecified product	PF3D7-1370500_S0_Sub0_	C1	reverse	chr11	1920280	1920462	1920268	1920811	TRUE
PF3D7-1370500	PF3D7_1148200	unspecified product	PF3D7-1370500_S0_Sub0_	C1	forward	chr11	1920386	1920590	1920268	1920811	TRUE
PF3D7-1370500	PF3D7_1148200	unspecified product	PF3D7-1370500_S0_Sub0_	C1	reverse	chr11	1920588	1920760	1920268	1920811	TRUE
PF3D7-1370500	PF3D7_1148200	unspecified product	PF3D7-1370500_S0_Sub0_	C1	forward	chr11	1920716	1920884	1920268	1920811	TRUE
PF3D7-1370500	PF3D7_1148200	unspecified product	PF3D7-1370500_S0_Sub0_	C1	reverse	chr11	1920809	1921024	1920268	1920811	TRUE
PF3D7-1370900			PF3D7-1370900_S0_Sub0_	C1	forward	chr11	1923308	1923492			TRUE
PF3D7-1370900			PF3D7-1370900_S0_Sub0_	C1	reverse	chr11	1923404	1923648			TRUE
PF3D7-1370900			PF3D7-1370900_S0_Sub0_	C1	forward	chr11	1923884	1924054			TRUE
PF3D7-1370900			PF3D7-1370900_S0_Sub0_	C1	reverse	chr11	1924045	1924210			TRUE
PF3D7-1371000	PF3D7_1148620	5.8S ribosomal RNA	PF3D7-1371000_S0_Sub0_	C1	reverse	chr11	1928534	1928747	1928510	1928677	TRUE
PF3D7-1371000	PF3D7_1148620	5.8S ribosomal RNA	PF3D7-1371000_S0_Sub0_	C1	reverse	chr11	1928670	1928856	1928510	1928677	TRUE
PF3D7-1371000	PF3D7_1148640	28S ribosomal RNA	PF3D7-1371000_S0_Sub0_	C1	reverse	chr11	1928875	1929058	1928934	1933138	TRUE
PF3D7-1371000	PF3D7_1148640	28S ribosomal RNA	PF3D7-1371000_S0_Sub0_	C1	reverse	chr11	1928878	1929088	1928934	1933138	TRUE
PF3D7-1371000	PF3D7_1148640	28S ribosomal RNA	PF3D7-1371000_S0_Sub0_	C1	forward	chr11	1929064	1929266	1928934	1933138	TRUE
PF3D7-1371000	PF3D7_1148640	28S ribosomal RNA	PF3D7-1371000_S0_Sub0_	C1	reverse	chr11	1929217	1929412	1928934	1933138	TRUE
PF3D7-1371000	PF3D7_1148640	28S ribosomal RNA	PF3D7-1371000_S0_Sub0_	C1	forward	chr11	1929391	1929607	1928934	1933138	TRUE
PF3D7-1371000	PF3D7_1148640	28S ribosomal RNA	PF3D7-1371000_S0_Sub0_	C1	reverse	chr11	1929591	1929811	1928934	1933138	TRUE
PF3D7-1371000	PF3D7_1148640	28S ribosomal RNA	PF3D7-1371000_S0_Sub0_	C1	forward	chr11	1929808	1930025	1928934	1933138	TRUE
PF3D7-1371000	PF3D7_1148640	28S ribosomal RNA	PF3D7-1371000_S0_Sub0_	C1	reverse	chr11	1929972	1930188	1928934	1933138	TRUE
PF3D7-1371000	PF3D7_1148640	28S ribosomal RNA	PF3D7-1371000_S0_Sub0_	C1	forward	chr11	1930115	1930316	1928934	1933138	TRUE
PF3D7-1371000	PF3D7_1148640	28S ribosomal RNA	PF3D7-1371000_S0_Sub0_	C1	forward	chr11	1930223	1930407	1928934	1933138	TRUE
PF3D7-1371000	PF3D7_1148640	28S ribosomal RNA	PF3D7-1371000_S0_Sub0_	C1	forward	chr11	1930440	1930652	1928934	1933138	TRUE
PF3D7-1371000	PF3D7_1148640	28S ribosomal RNA	PF3D7-1371000_S0_Sub0_	C1	forward	chr11	1930720	1930939	1928934	1933138	TRUE
PF3D7-1371000	PF3D7_1148640	28S ribosomal RNA	PF3D7-1371000_S0_Sub0_	C1	reverse	chr11	1930874	1931075	1928934	1933138	TRUE
PF3D7-1371000	PF3D7_1148640	28S ribosomal RNA	PF3D7-1371000_S0_Sub0_	C1	reverse	chr11	1931054	1931262	1928934	1933138	TRUE
PF3D7-1371000	PF3D7_1148640	28S ribosomal RNA	PF3D7-1371000_S0_Sub0_	C1	reverse	chr11	1931278	1931491	1928934	1933138	TRUE
PF3D7-1371000	PF3D7_1148640	28S ribosomal RNA	PF3D7-1371000_S0_Sub0_	C1	reverse	chr11	1931365	1931584	1928934	1933138	TRUE
PF3D7-1371000	PF3D7_1148640	28S ribosomal RNA	PF3D7-1371000_S0_Sub0_	C1	forward	chr11	1931768	1931984	1928934	1933138	TRUE
PF3D7-1371000	PF3D7_1148640	28S ribosomal RNA	PF3D7-1371000_S0_Sub0_	C1	forward	chr11	1932098	1932287	1928934	1933138	TRUE
PF3D7-1371000	PF3D7_1148640	28S ribosomal RNA	PF3D7-1371000_S0_Sub0_	C1	forward	chr11	1932104	1932306	1928934	1933138	TRUE
PF3D7-1371000	PF3D7_1148640	28S ribosomal RNA	PF3D7-1371000_S0_Sub0_	C1	forward	chr11	1932215	1932417	1928934	1933138	TRUE
PF3D7-1371000	PF3D7_1148640	28S ribosomal RNA	PF3D7-1371000_S0_Sub0_	C1	forward	chr11	1932904	1933114	1928934	1933138	TRUE

Supplementary Table 6. *Pfhrp2/3* MIP panel probe sequences.

MIP	probe_id	Probe Sequence
PF3D7-0831400_	ref	CTTGGGTATCTTTTCCATATATAATAACGCNNNNAGATCGGAAGAGCACACGTGACTCGCCAAGCTGAAGNNNNNNNNNNNTTATCTTTCCTTGCTTTTTCTCTC
PF3D7-0831400_	ref	GATACAGCAGTGAATACTAAAAGAAGAAANNNNAGATCGGAAGAGCACACGTGACTCGCCAAGCTGAAGNNNNNNNNNNNATAGATTCCATGAAACTTACAGATAAA
PF3D7-0831400_	ref	TAAGGTATTTGTATAGGCATCGATAGATAANNNNAGATCGGAAGAGCACACGTGACTCGCCAAGCTGAAGNNNNNNNNNNNACGCATGAATTCTTTAAATATATCTTTTA
PF3D7-0831400_	ref	AAGCAGGTAAATATATAAACGAGGCANNNNAGATCGGAAGAGCACACGTGACTCGCCAAGCTGAAGNNNNNNNNNNNGAACAAAATGATGAATCTAAATGTGAA
PF3D7-0831400_	ref	TTTGTCATTTAATCTGAATCCATTTAAAGAACNNNNAGATCGGAAGAGCACACGTGACTCGCCAAGCTGAAGNNNNNNNNNNNCAGCTTGATCAGTATTATTTGACT
PF3D7-0831400_	ref	TGGAGAACATGATGAAGAAGAAGAACNNNNAGATCGGAAGAGCACACGTGACTCGCCAAGCTGAAGNNNNNNNNNNNGGTCATTCAGATATAACTTTATGAATTT
PF3D7-0831400_	ref	TACAGCAATAAATAGAAATACGTTGGTATATNNNNAGATCGGAAGAGCACACGTGACTCGCCAAGCTGAAGNNNNNNNNNNNTCTTTTATTTCAACATATTTATGGAGTG
PF3D7-0831400_	ref	ACAAAAATTCGTTATCCTGTGGTTTTNNNNAGATCGGAAGAGCACACGTGACTCGCCAAGCTGAAGNNNNNNNNNNNAAATTCTCATCATTATAATAAAATGAATATATATA
PF3D7-0831500_	ref	CAGCATTATATATACAATTCAAAGTAAGTTCANNNNAGATCGGAAGAGCACACGTGACTCGCCAAGCTGAAGNNNNNNNNNNNGCACCGTTTATAGGAAATATTATAAAA
PF3D7-0831500_	ref	TCCATGATGTCTTTTTCTTATATCCATTATTTNNNNAGATCGGAAGAGCACACGTGACTCGCCAAGCTGAAGNNNNNNNNNNNGGATATATACAGTTCAACCATATAATTAATATATA
PF3D7-0831500_	ref	TGTACAAGTAGAAAATTTTCCTTCTTTAAATCNNNNAGATCGGAAGAGCACACGTGACTCGCCAAGCTGAAGNNNNNNNNNNNGTAGTATTATTTTTATTTACATATACCCCAATAT
PF3D7-0831500_	ref	AACACATGAACTATTCTCCCCATATTTATTNNNNAGATCGGAAGAGCACACGTGACTCGCCAAGCTGAAGNNNNNNNNNNNATTCTTACTATTATTATTACTATTATTATTGTTTT
PF3D7-0831500_	ref	TAATGATATGACAAAACAATTAACAAAGGAAGNNNNAGATCGGAAGAGCACACGTGACTCGCCAAGCTGAAGNNNNNNNNNNNTGATGAAAAAGACGAATCGAATAA
PF3D7-0831500_	ref	TTTCCTGAATCATTACTAACATTACTTATATTATTNNNNAGATCGGAAGAGCACACGTGACTCGCCAAGCTGAAGNNNNNNNNNNNCATTTAGATCAATTAATTTTTCATCTAATATAT
PF3D7-0831500_	ref	GTTTAATGGAAAAATATTTAGATGAATTTGATGAANNNNAGATCGGAAGAGCACACGTGACTCGCCAAGCTGAAGNNNNNNNNNNNCAAAGGAAGAATTATTTTATGTTCTTGA
PF3D7-0831500_	ref	ACGTTTACTTTCATATTTCTTTTTATATTTCTGTANNNNAGATCGGAAGAGCACACGTGACTCGCCAAGCTGAAGNNNNNNNNNNNACCATACATATAATTTATTAACTTTTCTAAAT
PF3D7-0831500_	ref	TAATATGTGTTTAAAGTGTAACATAAAGTAATAGANNNNAGATCGGAAGAGCACACGTGACTCGCCAAGCTGAAGNNNNNNNNNNNACATAAAAGTCCTTCATTAGATGATATA
PF3D7-0831600_	ref	AAAATGTACAGGAAATATGACAACCATAAAANNNNAGATCGGAAGAGCACACGTGACTCGCCAAGCTGAAGNNNNNNNNNNNCATTTGGATTTGAATCTCCATTTAAAT
PF3D7-0831600_	ref	TTTCATTCACATTTTTACTCTCATTAATGGAANNNNAGATCGGAAGAGCACACGTGACTCGCCAAGCTGAAGNNNNNNNNNNNGGTACAATATATATATCATCATCTGCAT
PF3D7-0831600_	ref	ATGTAAGTCGAAATCCGTATCTTCCNNNNAGATCGGAAGAGCACACGTGACTCGCCAAGCTGAAGNNNNNNNNNNNCATTCCGTAAGAACTATAACGTTT
PF3D7-0831600_	ref	ATAAGCCCTAGTATCGTAATCTATGAAAGNNNNAGATCGGAAGAGCACACGTGACTCGCCAAGCTGAAGNNNNNNNNNNNAAAAATTAAGTCTTACATTCCAAAGAAT
PF3D7-0831600_	ref	CAAAGAATGATCAAATAATGTAGGAAGTTCTNNNNAGATCGGAAGAGCACACGTGACTCGCCAAGCTGAAGNNNNNNNNNNNTCATTCATAAAATTACTATCTAAGGGAATAT
PF3D7-0831600_	ref	AAGGGAATATGTTTCATGAATGAGGTNNNNAGATCGGAAGAGCACACGTGACTCGCCAAGCTGAAGNNNNNNNNNNNTTGTAACATGGAAGGAACATTATC
PF3D7-0831600_	ref	TTGGAGCTTAAGACCTTGAGTTACANNNNAGATCGGAAGAGCACACGTGACTCGCCAAGCTGAAGNNNNNNNNNNNACGTAAAAAGTGAAAAATAGACAAATA
PF3D7-0831600_	ref	TTCTAGTATCGTTAAATTCTTATGTAGTTCATCANNNNAGATCGGAAGAGCACACGTGACTCGCCAAGCTGAAGNNNNNNNNNNNTCTATTGATTGTTCCTTTAATAAATTTTCA
PF3D7-0831600_	ref	TGCAATTTCTACGTAAAAAGTGAAAAATAGACANNNNAGATCGGAAGAGCACACGTGACTCGCCAAGCTGAAGNNNNNNNNNNNAAAATAATAACATGTCTTGAATACATTTAGT
PF3D7-0831600_	ref	TCACGTTTTTAGTACATGTTAATTCATTTTTACANNNNAGATCGGAAGAGCACACGTGACTCGCCAAGCTGAAGNNNNNNNNNNNAACATCTCCCAAATAATTTATTGGATG
PF3D7-0831600_	ref	TTACGCATTTGTCTCAATAATTCTAAAATAACCANNNNAGATCGGAAGAGCACACGTGACTCGCCAAGCTGAAGNNNNNNNNNNNGATAATTTGAGAAATAAAAACCTGCA
PF3D7-0831600_	ref	TCAGATCTAATACTCGATTATGTAATTCTCTTCTTNNNNAGATCGGAAGAGCACACGTGACTCGCCAAGCTGAAGNNNNNNNNNNNAAAATAAAATATCGTCAACATTCTTTCT
PF3D7-0831600_	ref	TGAGCTCTTTTCAATTTATAATTTCAATACAGACNNNNAGATCGGAAGAGCACACGTGACTCGCCAAGCTGAAGNNNNNNNNNNNATGCTAATGAGAGATGCATATGA
PF3D7-0831600_	ref	CTTTTTAACTAAAGGTGTTGCTGTTAAATATTTTCNNNNAGATCGGAAGAGCACACGTGACTCGCCAAGCTGAAGNNNNNNNNNNNCTTGTAAGTGTCTTCTATTTTTCTAAA
PF3D7-0831600_	ref	AATTGTGTATTTCTAATTCCCTTTGTTCCNNNNAGATCGGAAGAGCACACGTGACTCGCCAAGCTGAAGNNNNNNNNNNNGGTGGTAATAAACTATTAGTTATGCTAT
PF3D7-0831600_	ref	GTTTATTGAATAGCCATAGCGAAACATTNNNNAGATCGGAAGAGCACACGTGACTCGCCAAGCTGAAGNNNNNNNNNNNCAATCAAGTTTTTATGATATTGCAAAATA
PF3D7-0831600_	ref	TAAAAAGGTAGATCAATTCCTTGGTAGAANNNNAGATCGGAAGAGCACACGTGACTCGCCAAGCTGAAGNNNNNNNNNNNGCATATCTTACTTTTGCTTATGTATATAAT
PF3D7-0831600_	ref	TGGAGGTTTTTCGACTTCTTTAAGAGNNNNAGATCGGAAGAGCACACGTGACTCGCCAAGCTGAAGNNNNNNNNNNNAGATCAATTCCTTGGTAGAAAATTT
PF3D7-0831600_	ref	AAGTCGAAAAACCTCCAGGAAAATANNNNAGATCGGAAGAGCACACGTGACTCGCCAAGCTGAAGNNNNNNNNNNNAAATATCTCTCAAGGAAACGAAAATT
PF3D7-0831600_	ref	TGGTTATCTTGATGAGGCAATGGANNNNAGATCGGAAGAGCACACGTGACTCGCCAAGCTGAAGNNNNNNNNNNNTCATGTATGCCCTTTGTTTTATG
PF3D7-0831600_	ref	AATCCGATGAAGATGATCAAAAGGCNNNNAGATCGGAAGAGCACACGTGACTCGCCAAGCTGAAGNNNNNNNNNNNAGAAAACGTGGTTATCTTGATGA
PF3D7-0831600_	ref	ACTGATGATTACAAAAATACCTGAAGATGNNNNAGATCGGAAGAGCACACGTGACTCGCCAAGCTGAAGNNNNNNNNNNNGGTAAAAATGAGGAATTTCTAAATGATA
PF3D7-0831600_	ref	TGGAAGAATTAGATAACATATCAAATCAATATGGANNNNAGATCGGAAGAGCACACGTGACTCGCCAAGCTGAAGNNNNNNNNNNNATTGCACTAGAAGAATTAAATAAAGTATTT
PF3D7-0831600_	ref	TAATTTTAATATCATAAAATGTCCCAGAGCTGNNNNAGATCGGAAGAGCACACGTGACTCGCCAAGCTGAAGNNNNNNNNNNNCCAGGATATTACATTCCATATTTGTATT
PF3D7-0831600_	ref	AAGATGGATTCTTTTCATATATATGTAATGATCCTNNNNAGATCGGAAGAGCACACGTGACTCGCCAAGCTGAAGNNNNNNNNNNNGTGTTTTCAGAATGCTGTATATTATT
PF3D7-0831700_	ref	TGCAATTTATTGTCTATCAAAAAGGAATAAAGNNNNAGATCGGAAGAGCACACGTGACTCGCCAAGCTGAAGNNNNNNNNNNNCAGTATATAATCCTATAATGACTAAAATATATCAA
PF3D7-0831700_	ref	AAATGTTCCTCCTCCTAAATCGAAAANNNNAGATCGGAAGAGCACACGTGACTCGCCAAGCTGAAGNNNNNNNNNNNGCTTGTGCGGATGATGATAATA
PF3D7-0831700_	ref	TTTTTGAAGTTAAAGCTACCTCAGGANNNNAGATCGGAAGAGCACACGTGACTCGCCAAGCTGAAGNNNNNNNNNNNCTGTTCCAGCTTATTTCAATGATT
PF3D7-0831700_	ref	AATCATTGGTTTTCCATCAGAACCANNNNAGATCGGAAGAGCACACGTGACTCGCCAAGCTGAAGNNNNNNNNNNNACCATATGCTATAGCTGCTGC
PF3D7-0831700_	ref	AAAACCAGTTAAAAATGCTGTTATTACTGTNNNNAGATCGGAAGAGCACACGTGACTCGCCAAGCTGAAGNNNNNNNNNNNGATGCAAAAAGATTAATAGGAAGAAAAT
PF3D7-0831700_	ref	TACATATACCAACACATGAATAGGTGGTNNNNAGATCGGAAGAGCACACGTGACTCGCCAAGCTGAAGNNNNNNNNNNNTCAGAACCACCTTTTACAGTAAAT
PF3D7-0831700_	ref	TCCAGGAATCCAGAAAATACAGTTTTNNNNAGATCGGAAGAGCACACGTGACTCGCCAAGCTGAAGNNNNNNNNNNNACAGTACATACAGCAAGTAACAAT
PF3D7-0831700_	ref	AGGCTTGATTTTGACAATAACTGACTNNNNAGATCGGAAGAGCACACGTGACTCGCCAAGCTGAAGNNNNNNNNNNNGTACCTAAATCAATACCAATTGCA
PF3D7-0831700_	ref	TAACTGTTTTCATACATTTTTCTGAATCATCTNNNNAGATCGGAAGAGCACACGTGACTCGCCAAGCTGAAGNNNNNNNNNNNGTCCATTATTTTGTGCTTCTTTATTTT
PF3D7-0831700_	ref	AAAAATCAAACAGCAGAAACTGAAGANNNNAGATCGGAAGAGCACACGTGACTCGCCAAGCTGAAGNNNNNNNNNNNAAGATGATATCGATCGTATGGTAA
PF3D7-0831700_	ref	TGGGATACCTTCTAATTGAAATTTTCCTNNNNAGATCGGAAGAGCACACGTGACTCGCCAAGCTGAAGNNNNNNNNNNNTATTTTGTTCATCCTCTTCTTTATATTT
PF3D7-0831700_	ref	AAAACCAAATTACTATTACAAATGATAAAGGAAGANNNNAGATCGGAAGAGCACACGTGACTCGCCAAGCTGAAGNNNNNNNNNNNCAAATCTTTACAACATATGCTGATAA
PF3D7-0831700_	ref	AACAGCTTCATCTGGATTAATGGCNNNNAGATCGGAAGAGCACACGTGACTCGCCAAGCTGAAGNNNNNNNNNNNAAACTTGAATTAAAACACCTGGTT
PF3D7-0831700_	ref	ACCAAATTAATTGAAAGAAATACAACTATACCAACNNNNAGATCGGAAGAGCACACGTGACTCGCCAAGCTGAAGNNNNNNNNNNNCCAAAAATACAACAACTTATCAAAGATT
PF3D7-0831700_	ref	TTATAATCGATTCCATCAAATAAAGAATCAACTTCNNNNAGATCGGAAGAGCACACGTGACTCGCCAAGCTGAAGNNNNNNNNNNNTTAATGGCTTTACATGGTTCTTTT
PF3D7-0831700_	ref	AAAAATGGATAAAAGCCAAGTACATGAAANNNNAGATCGGAAGAGCACACGTGACTCGCCAAGCTGAAGNNNNNNNNNNNGGTAAAGATGTTTCCAAAAATTCAAAAT
PF3D7-0831750_	ref	TGTACATATGAATGATGAAAATATTATCTTAGATTNNNNAGATCGGAAGAGCACACGTGACTCGCCAAGCTGAAGNNNNNNNNNNNGTATCGTATGTGTAAAACTTTTGATTT
PF3D7-0831750_	ref	TTTGTATTCAAAAACATTTTAAAGACCTCANNNNAGATCGGAAGAGCACACGTGACTCGCCAAGCTGAAGNNNNNNNNNNNTCATAGCATATGTCTTTCTTAAGTTTT
PF3D7-0831750_	ref	AATACTGATGTAAATAAACATAAACTTAATGATTCNNNNAGATCGGAAGAGCACACGTGACTCGCCAAGCTGAAGNNNNNNNNNNNCACTCAGAAATTATTATCGTCAACA
PF3D7-0831750_	ref	TTTTTATAATACTATCAACTTTTCCATACCTTAAANNNNAGATCGGAAGAGCACACGTGACTCGCCAAGCTGAAGNNNNNNNNNNNTACATATTATATCATCCCCTCCTAATTT
PF3D7-0831750_	ref	AAAATTTATATGTTATTGGAGTGAAATTATTGCNNNNAGATCGGAAGAGCACACGTGACTCGCCAAGCTGAAGNNNNNNNNNNNAAATATAGCACGTAAAGTTTTAATTAATATAT
PF3D7-0831750_	ref	TTAGACAAACACACTTCTTCACAAATTNNNNAGATCGGAAGAGCACACGTGACTCGCCAAGCTGAAGNNNNNNNNNNNAAATTTACATAAACATGAAAATAAGTATATTT
PF3D7-0831750_	ref	ATGGGACAGGTGTAAATATAGTTTATAATTTTNNNNAGATCGGAAGAGCACACGTGACTCGCCAAGCTGAAGNNNNNNNNNNNTACTTAATAAAAGAGTTAAACTTGTTAAAAT
PF3D7-0831900_	ref	GCTTATGTTTGTATATGATTGGATTTTTATATATTNNNNAGATCGGAAGAGCACACGTGACTCGCCAAGCTGAAGNNNNNNNNNNNTAGCATATTTATATTTCTACATATTTTTAATTTT
PF3D7-0831900_	ref	AATCATATACAAACATAAGCAAAGAAACTTAANNNNAGATCGGAAGAGCACACGTGACTCGCCAAGCTGAAGNNNNNNNNNNNGGTAGTTTTCAACTTTTTCTAAGCT
PF3D7-0832000_	ref	ACTTGTTCACTATTTCTACTTTTAGTTTTTGTTTCNNNNAGATCGGAAGAGCACACGTGACTCGCCAAGCTGAAGNNNNNNNNNNNTAATTATCATTTACCTTATTTGATAAAGATAAT
PF3D7-0832000_	ref	ATATATACACCAATGAATATTACAAATCAAATGANNNNAGATCGGAAGAGCACACGTGACTCGCCAAGCTGAAGNNNNNNNNNNNAAAAATTATTGACCAATTGAACCAG
PF3D7-0832000_	ref	AAGGAATGAATATATTATTGAGTAAATATTGATGANNNNAGATCGGAAGAGCACACGTGACTCGCCAAGCTGAAGNNNNNNNNNNNATCATTAGATTGTTGGTATGTGTTTAA
PF3D7-0832000_	ref	TTCCTAATAAAACCCTCATAAACAATTACANNNNAGATCGGAAGAGCACACGTGACTCGCCAAGCTGAAGNNNNNNNNNNNATACCACTCTAAAATGTTATTATTTGC
PF3D7-0832000_	ref	AATTAAGTATTCATACAAAATGTGCAATAAGTTTNNNNAGATCGGAAGAGCACACGTGACTCGCCAAGCTGAAGNNNNNNNNNNNTTTGTGATAGTAAAAATTTATTAAATAGAAAG
PF3D7-0832000_	ref	TACCACTCTAAAATGTTATTATTTGCCTTNNNNAGATCGGAAGAGCACACGTGACTCGCCAAGCTGAAGNNNNNNNNNNNTTATATTTTCTATCATATTTCTTATAAAACTTATT
PF3D7-0832100_	ref	AATTAGTAGTTATACCTGCTATTTTATTAGTTTTTNNNNAGATCGGAAGAGCACACGTGACTCGCCAAGCTGAAGNNNNNNNNNNNCATGTAATATAGGCGAAATAATGAATT
PF3D7-0832100_	ref	CTTATGTTTTGTAATGGTGAGGTTCATANNNNAGATCGGAAGAGCACACGTGACTCGCCAAGCTGAAGNNNNNNNNNNNGACAAAGGAGACAACTGAAACTA
PF3D7-0832100_	ref	TGAACAAGAATATTGGGATTATTGAAATTGNNNNAGATCGGAAGAGCACACGTGACTCGCCAAGCTGAAGNNNNNNNNNNNGTAGTTATACCTGCTATTTTATTAGTTT
PF3D7-0832100_	ref	ATATATTAGAATGTCGCATGCTAATATCATTGANNNNAGATCGGAAGAGCACACGTGACTCGCCAAGCTGAAGNNNNNNNNNNNTACAGCAATTTTTGTTACTATTCAATT
PF3D7-0832100_	ref	ACTAATATCCCTCCACATTTCAAACANNNNAGATCGGAAGAGCACACGTGACTCGCCAAGCTGAAGNNNNNNNNNNNGAAATTGTCTGCATTAATAATTGATTTC
PF3D7-0832100_	ref	TACTTTATATGCTATAAGTGTTTAGAAAGATACAGNNNNAGATCGGAAGAGCACACGTGACTCGCCAAGCTGAAGNNNNNNNNNNNTTCAACAAATTATTTTTAAAATGCAAAAT
PF3D7-0832100_	ref	AAATTTTGCATTACTTCTTTCATTTCTGAGNNNNAGATCGGAAGAGCACACGTGACTCGCCAAGCTGAAGNNNNNNNNNNNTTTCAACTTTAACTGCTACTGATTT
PF3D7-0832100_	ref	TGACAAACATATGATCAAAAACAGACAANNNNAGATCGGAAGAGCACACGTGACTCGCCAAGCTGAAGNNNNNNNNNNNATATATAATACTATATATATCCATATTTTTACTGG
PF3D7-0832200-2	ref	ATTTAAAAATTTTCTCCTAATTAAACGAACATTCNNNNAGATCGGAAGAGCACACGTGACTCGCCAAGCTGAAGNNNNNNNNNNNTAAATACTCCTGCATCATTTGTTT
PF3D7-0832200-2	ref	AAAATATAATGATGTTACAACTAAAAGAAAATGTGNNNNAGATCGGAAGAGCACACGTGACTCGCCAAGCTGAAGNNNNNNNNNNNTGTTCAGAATATAATAATATGTGACAAAA
PF3D7-0832200-2	ref	GTTATCATTATTTTGGAGGTAGGAAACCNNNNAGATCGGAAGAGCACACGTGACTCGCCAAGCTGAAGNNNNNNNNNNNCAACTAAAAGAAAATGTGATAATATAAATTATAAT
PF3D7-0832200-2	ref	ATTTAATACACATAAGAAAATATTTACAGAATGGANNNNAGATCGGAAGAGCACACGTGACTCGCCAAGCTGAAGNNNNNNNNNNNCGTTATCATTATTTTGGAGGTAGG
PF3D7-0832200-2	ref	TTTCACGTACATTTTTCATAGATACAGGTNNNNAGATCGGAAGAGCACACGTGACTCGCCAAGCTGAAGNNNNNNNNNNNTTTTCTAATCCTTGGAGCTCTTT
PF3D7-0832300_	ref	ATTCTCCTTAAGAAAATGAATTAACATTCTGATGNNNNAGATCGGAAGAGCACACGTGACTCGCCAAGCTGAAGNNNNNNNNNNNAAAGAACTTGTATATAGGAGTATATGG
PF3D7-0832500_	ref	AACTTGACGGATTAACAATTGATGTCNNNNAGATCGGAAGAGCACACGTGACTCGCCAAGCTGAAGNNNNNNNNNNNATCATGGCAATATTGAAACTAGTAAT
PF3D7-1369900_	ref	CAGAGTGCAATAAACAGTATATATTTTACATTNNNNAGATCGGAAGAGCACACGTGACTCGCCAAGCTGAAGNNNNNNNNNNNCCCTCTGGGCTCAATAAATTTAATA
PF3D7-1369900_	ref	TTTTCCTTGTATTTCTTTCTTGTTCAATTTNNNNAGATCGGAAGAGCACACGTGACTCGCCAAGCTGAAGNNNNNNNNNNNTATTGTTTTTCTTATTTTGCTTAAATATTAAAAA
PF3D7-1369900_	ref	TGGGGGTTCGAATCCCTCTGGNNNNAGATCGGAAGAGCACACGTGACTCGCCAAGCTGAAGNNNNNNNNNNNTTATTCTTTTATTAATTTTAGAATAAGGAAAAA
PF3D7-1369900_	ref	ATTACGCCACTGGTGCTAATTTGNNNNAGATCGGAAGAGCACACGTGACTCGCCAAGCTGAAGNNNNNNNNNNNCTATATTTGTAATATTCAAAAATTTTTACTTTTT
PF3D7-1370100_	ref	TCTACCGCTGAGCTACACGCNNNNAGATCGGAAGAGCACACGTGACTCGCCAAGCTGAAGNNNNNNNNNNNAAATATTTATTTATAATCCAGGCCACA
PF3D7-1370100_	ref	AGATCAGCGGGTCCATGGTTNNNNAGATCGGAAGAGCACACGTGACTCGCCAAGCTGAAGNNNNNNNNNNNAGAATATCGTTTTAAAGAAGAAATAAACA
PF3D7-1370200_	ref	ATCGAAGTGACCCGGGTTCGANNNNAGATCGGAAGAGCACACGTGACTCGCCAAGCTGAAGNNNNNNNNNNNAAGAAATAAAAAGAAATGCATGTAATTT
PF3D7-1370200_	ref	GGCAACGAAGTATTCTACCACTAGACNNNNAGATCGGAAGAGCACACGTGACTCGCCAAGCTGAAGNNNNNNNNNNNTTTATTTTATTTATTCTTTATTATTTATATGGAAC
PF3D7-1370300_	ref	TGCTTGCTCTGCCATTTTTAGATATTNNNNAGATCGGAAGAGCACACGTGACTCGCCAAGCTGAAGNNNNNNNNNNNCATGTTTTCCTTCACCATCAGTT
PF3D7-1370300_	ref	TTCTTATACCACTCAGTAAGTTCATTATAAAANNNNAGATCGGAAGAGCACACGTGACTCGCCAAGCTGAAGNNNNNNNNNNNTGCTAGGAATTGGTTTACTAATTTTAT
PF3D7-1370300_	ref	TCCATCTAAAGCTTCAGAAAGAGAAGNNNNAGATCGGAAGAGCACACGTGACTCGCCAAGCTGAAGNNNNNNNNNNNTTGGGTTTATATATAATAATAAAAAGGTATTTA
PF3D7-1370300_	ref	ATCACGACCATGATCACGAACANNNNAGATCGGAAGAGCACACGTGACTCGCCAAGCTGAAGNNNNNNNNNNNAAATATAGTAATATTATTATTTTAAGTGATACATT
PF3D7-1370300_	ref	TAGAAATGAGGTGCATGGTGATAATGNNNNAGATCGGAAGAGCACACGTGACTCGCCAAGCTGAAGNNNNNNNNNNNTGCTTTATTTTTGCATGTACATACA
PF3D7-1370300_	ref	AATTAAATGCTTCATAAGATTCTTATATGGTGNNNNAGATCGGAAGAGCACACGTGACTCGCCAAGCTGAAGNNNNNNNNNNNCACGCTCATGAACTTGATCAT
PF3D7-1370500_	ref	ATACGTTAAACCTTAAAATGAATTTACTTATTGANNNNAGATCGGAAGAGCACACGTGACTCGCCAAGCTGAAGNNNNNNNNNNNTTTCTTTACTACTACTAATATATATTATATGTTAC
PF3D7-1370500_	ref	ATGAGAACATATCATATAATATATAGTCCACTATTNNNNAGATCGGAAGAGCACACGTGACTCGCCAAGCTGAAGNNNNNNNNNNNTATTGATAGCATGTTATTGTTTGTTAT
PF3D7-1370500_	ref	AACACAAAAACTATCATAAATCTTTTTACTCTTCNNNNAGATCGGAAGAGCACACGTGACTCGCCAAGCTGAAGNNNNNNNNNNNACTACATGACGTATATACCATAACAAA
PF3D7-1370500_	ref	ATTGGTATCAAATAAAAATGAAAAAGACTACGNNNNAGATCGGAAGAGCACACGTGACTCGCCAAGCTGAAGNNNNNNNNNNNATTATAAAATTGAACATTGCATTAGAAAA
PF3D7-1370500_	ref	TTAACCCTCCCCGTAGTTAAAACTNNNNAGATCGGAAGAGCACACGTGACTCGCCAAGCTGAAGNNNNNNNNNNNACACCTACCCATATACCACTTAC
PF3D7-1370900_	ref	TTTTTGTACGTCTTAAATTCATAATTAAATGTGTNNNNAGATCGGAAGAGCACACGTGACTCGCCAAGCTGAAGNNNNNNNNNNNAATAATTAAAATATATGGGAAGTGTGTG
PF3D7-1370900_	ref	AAACATATATATATTATTTCTTCTGATTTTCAACGNNNNAGATCGGAAGAGCACACGTGACTCGCCAAGCTGAAGNNNNNNNNNNNTAACACATTTAATTATGAATTTAAGACTAATT
PF3D7-1370900_	ref	GGATTTATTGAAGAAATGAAAATCTATGAAGANNNNAGATCGGAAGAGCACACGTGACTCGCCAAGCTGAAGNNNNNNNNNNNTTAATAAGACTTAAATATATATAATTAATTGTTACA
PF3D7-1370900_	ref	AATATTTCGTTATCCATCTTCACACATATATNNNNAGATCGGAAGAGCACACGTGACTCGCCAAGCTGAAGNNNNNNNNNNNAAATCTTATTAATAGTTATAATAAAATTATTAGCA
PF3D7-1371000_	ref	CATCCATCGTTAAGATTGTGGCTATNNNNAGATCGGAAGAGCACACGTGACTCGCCAAGCTGAAGNNNNNNNNNNNAATCCAAATTCTCTTTTTCTTCTATGA
PF3D7-1371000_	ref	TAGGAATACCACGTTAATTTGCCCANNNNAGATCGGAAGAGCACACGTGACTCGCCAAGCTGAAGNNNNNNNNNNNTTACATATAAACTTTCTCTCCTCTAGAA
PF3D7-1371000_	ref	ACAATGATTAGAGGGTTTAGGGGATNNNNAGATCGGAAGAGCACACGTGACTCGCCAAGCTGAAGNNNNNNNNNNNTTGAGTATGAGTACATTTGTTAGGAC
PF3D7-1371000_	ref	TCACTTTGGCGTAAATAAGATAACTCCAANNNNAGATCGGAAGAGCACACGTGACTCGCCAAGCTGAAGNNNNNNNNNNNTTCATTACAATCACGTTTAGGGG
PF3D7-1371000_	ref	GATACCGGGCCATAAGAAGGTAGNNNNAGATCGGAAGAGCACACGTGACTCGCCAAGCTGAAGNNNNNNNNNNNGCGTAAATAAGATAACTCCAAGTG
PF3D7-1371000_	ref	ATCTTCGTCTCAAAAGGGAAACAGGNNNNAGATCGGAAGAGCACACGTGACTCGCCAAGCTGAAGNNNNNNNNNNNAGCTCAGATCTTTTTATGAGTAGAA
PF3D7-1371000_	ref	GCTAACTCTTGTACAAACATAGTTGACAAGNNNNAGATCGGAAGAGCACACGTGACTCGCCAAGCTGAAGNNNNNNNNNNNTTCCAAGGTTTGCTATAAAATTAAAGT
PF3D7-1371000_	ref	GTAAAACCTATGTCAAGGAGTTCATTTGTNNNNAGATCGGAAGAGCACACGTGACTCGCCAAGCTGAAGNNNNNNNNNNNATAATTAAATTCCCTTCTGTATATGCTC
PF3D7-1371000_	ref	CTTCTGTATATGCTCCGTTCCCTCCNNNNAGATCGGAAGAGCACACGTGACTCGCCAAGCTGAAGNNNNNNNNNNNTCTGTAATTTTATTTTCAACCCCC
PF3D7-1371000_	ref	TGCGGTTATTGGTACACTCATTATTGNNNNAGATCGGAAGAGCACACGTGACTCGCCAAGCTGAAGNNNNNNNNNNNATAAAATGAAAGCGAAAACAGGAA
PF3D7-1371000_	ref	TTTGTGAAACGACTTAGAAGGTGTAGTNNNNAGATCGGAAGAGCACACGTGACTCGCCAAGCTGAAGNNNNNNNNNNNCCCAGTGCTTTGAATGTTAAGT
PF3D7-1371000_	ref	GCTCAATGAGAACAGAAATCTCGTGNNNNAGATCGGAAGAGCACACGTGACTCGCCAAGCTGAAGNNNNNNNNNNNAAATGAAATACCACTACTTTTACAGTT
PF3D7-1371000_	ref	TAAACTTTCTCTCCTCTAGAACCAATCAAANNNNAGATCGGAAGAGCACACGTGACTCGCCAAGCTGAAGNNNNNNNNNNNCTTCCTTATAACAGAACAATTTCTA
PF3D7-1371000_	ref	CTCGTGTAGACTAAAAGGGGAAAAGNNNNAGATCGGAAGAGCACACGTGACTCGCCAAGCTGAAGNNNNNNNNNNNAAATACCACTACTTTTACAGTTGTTTTA
PF3D7-1371000_	ref	ATTCTGACTGTGCATATAAAGGTGTCTNNNNAGATCGGAAGAGCACACGTGACTCGCCAAGCTGAAGNNNNNNNNNNNTGTTTCTTATATTTCTCCCATTTCTATG
PF3D7-1371000_	ref	TGTTGAGGCTTATCTCTTGTGACAATTANNNNAGATCGGAAGAGCACACGTGACTCGCCAAGCTGAAGNNNNNNNNNNNACTGCTAATGTAAATGAGAAATTATACA
PF3D7-1371000_	ref	ACTTTCTCTCCTCTAGAACCAATCAANNNNAGATCGGAAGAGCACACGTGACTCGCCAAGCTGAAGNNNNNNNNNNNCACATAGTCTTTTTACAACTACGATATA
PF3D7-1371000_	ref	AGATTGGAGTACAAAGATGTGTGATACANNNNAGATCGGAAGAGCACACGTGACTCGCCAAGCTGAAGNNNNNNNNNNNGAAAAAGCTCAAGGAGATAATTCTATAT
PF3D7-1371000_	ref	ATATATTTATTAACTACGGGCCTTTCACCCNNNNAGATCGGAAGAGCACACGTGACTCGCCAAGCTGAAGNNNNNNNNNNNATCTTCTTACATTTTCCTTTCTTAATCC
PF3D7-1371000_	ref	TGCTTGTATGTTTGAGTATTCACTGCNNNNAGATCGGAAGAGCACACGTGACTCGCCAAGCTGAAGNNNNNNNNNNNTCAGGAATGAGCAGTTAAATAGTA
PF3D7-1371000_	ref	ACCCTTTTTCTTACTACTCCTCCCNNNNAGATCGGAAGAGCACACGTGACTCGCCAAGCTGAAGNNNNNNNNNNNCACTATTGTTTACCTCTTTCCTCA
PF3D7-1371000_	ref	AATAGTGCATTTGGTTTCTGGCTTGNNNNAGATCGGAAGAGCACACGTGACTCGCCAAGCTGAAGNNNNNNNNNNNCCGAAATGCGTATAATTATTCTGT
PF3D7-1371000_	ref	TATGTTTTTATTCATATCACATACGCTCGCNNNNAGATCGGAAGAGCACACGTGACTCGCCAAGCTGAAGNNNNNNNNNNNGATTGTAATGAACGATTTGCACG
PF3D7-1371500_	ref	CATTGTTTGAATAATTTGAACACTTTTTATAGTTANNNNAGATCGGAAGAGCACACGTGACTCGCCAAGCTGAAGNNNNNNNNNNNCATAATAATTGCATTATATATAATAATAATAGTAAGA
PF3D7-1371500_	ref	CATTCTTCAAAAGATAATAATACAAGTGATTTATTNNNNAGATCGGAAGAGCACACGTGACTCGCCAAGCTGAAGNNNNNNNNNNNAACGAAAGAATTTAACAAATACCAGT
PF3D7-1371500_	ref	TTATTATAATCATATGTCTGAGTAGTGAAAAAGGCNNNNAGATCGGAAGAGCACACGTGACTCGCCAAGCTGAAGNNNNNNNNNNNTTTGAACACTTTTTATAGTTATTCTTTATATT
PF3D7-1371500_	ref	TAAACCTAATTCCACTACAAACATTAACAANNNNAGATCGGAAGAGCACACGTGACTCGCCAAGCTGAAGNNNNNNNNNNNCCCTAAAAGTAAGCATCATATTAAAAT
PF3D7-1371500_	ref	TATGTGTATATAGGTTGAATGGTTTAGACTNNNNAGATCGGAAGAGCACACGTGACTCGCCAAGCTGAAGNNNNNNNNNNNTGTAATAAATGATAAACTAAAATATATAAGTACA
PF3D7-1371500_	ref	AAATTACTTGAAATAATAAAACTAAAAATGTTCCTNNNNAGATCGGAAGAGCACACGTGACTCGCCAAGCTGAAGNNNNNNNNNNNTCAAAAAGTTTTTCATATAAAATTAATAATTCTT
PF3D7-1371600_	ref	TGGATCAAAATAAGGATATGCTAAAAAGTGGNNNNAGATCGGAAGAGCACACGTGACTCGCCAAGCTGAAGNNNNNNNNNNNTTTGTTCTGTTTACATTGTGTATCAT
PF3D7-1371600_	ref	TGGTGGTCCACATACACCTTCANNNNAGATCGGAAGAGCACACGTGACTCGCCAAGCTGAAGNNNNNNNNNNNTTTATATCCCTCCAAACATCCAAA
PF3D7-1371600_	ref	AAAACAAGAAATTCAGACAGTGATGAAAGANNNNAGATCGGAAGAGCACACGTGACTCGCCAAGCTGAAGNNNNNNNNNNNTAAAGAAAGAAGAAGATGAAAAGGAAA
PF3D7-1371600_	ref	ATTTTAGGTAAATTTTCAGACACCACCCNNNNAGATCGGAAGAGCACACGTGACTCGCCAAGCTGAAGNNNNNNNNNNNTTTGGTCAGATGGTATTTTAGAAATTT
PF3D7-1371600_	ref	TATTGTTTCCTTATTCCCTTGATTAGAGTCCNNNNAGATCGGAAGAGCACACGTGACTCGCCAAGCTGAAGNNNNNNNNNNNAGATTGAAAAATATCAGCGCTTTT
PF3D7-1371600_	ref	CTGAAGAATCCTGAACTACACTTTCANNNNAGATCGGAAGAGCACACGTGACTCGCCAAGCTGAAGNNNNNNNNNNNTCCTCAGTCAAACTTTCATAATCA
PF3D7-1371600_	ref	TTCTTCAAGTCCTCCTTCTGAAATGANNNNAGATCGGAAGAGCACACGTGACTCGCCAAGCTGAAGNNNNNNNNNNNTCTTCAGAATTGTTTATTCCTTCTGTA
PF3D7-1371600_	ref	TGCCCCAATAAGACGAATACAATTATGNNNNAGATCGGAAGAGCACACGTGACTCGCCAAGCTGAAGNNNNNNNNNNNTCTTCGAGATAATAAAGAACAAGATGA
PF3D7-1371600_	ref	TCAGATTCATGAGAACTATCAGCATTTTCTNNNNAGATCGGAAGAGCACACGTGACTCGCCAAGCTGAAGNNNNNNNNNNNCCTCTCCACCTGTATTATCTTTAT
PF3D7-1371600_	ref	TAGTTATTACTTCCACACTTTCACTAGACCNNNNAGATCGGAAGAGCACACGTGACTCGCCAAGCTGAAGNNNNNNNNNNNCTTTTTCTTCAATTATTTGTTCATTCCT
PF3D7-1371600_	ref	GGTTATCTGTACAAATCCATTTCATTTCCTNNNNAGATCGGAAGAGCACACGTGACTCGCCAAGCTGAAGNNNNNNNNNNNACTCCATCTTATATCATCACATAATTTC
PF3D7-1371600_	ref	ATGATTTAGAAGGTTCAATGGACACCNNNNAGATCGGAAGAGCACACGTGACTCGCCAAGCTGAAGNNNNNNNNNNNAAAGATACAACCACATCTCATTCT
PF3D7-1371600_	ref	GTTCGTTCTTTATGACTTAATTGAGATGGANNNNAGATCGGAAGAGCACACGTGACTCGCCAAGCTGAAGNNNNNNNNNNNATCACTTTCTGCGCTTTCTTTATT
PF3D7-1371600_	ref	AAAGTTAATTTTGTGGGATTGCCTGGNNNNAGATCGGAAGAGCACACGTGACTCGCCAAGCTGAAGNNNNNNNNNNNTCCTGAAGCTTCAAATACTGATAATAA
PF3D7-1371600_	ref	ATATTGATCTATAACTCCTGCGTTCTCANNNNAGATCGGAAGAGCACACGTGACTCGCCAAGCTGAAGNNNNNNNNNNNCCAACAGGATATTCTATCTCTTTAGATA
PF3D7-1371600_	ref	ATTTGAAAACGAGAAAACAGTTGGCNNNNAGATCGGAAGAGCACACGTGACTCGCCAAGCTGAAGNNNNNNNNNNNTGATGTTACAAGGGGAAGTGTT
PF3D7-1371600_	ref	TAAAGAGAATTGTGGGCGCAATGNNNNAGATCGGAAGAGCACACGTGACTCGCCAAGCTGAAGNNNNNNNNNNNAAAGGAAACAGAGAAAAATATTGCA
PF3D7-1371600_	ref	TAACAAGTTTGATTCCAGTTTTACAGCTNNNNAGATCGGAAGAGCACACGTGACTCGCCAAGCTGAAGNNNNNNNNNNNCCACCATTCTTTTCGTAACTTTATATT
PF3D7-1371600_	ref	AAAGATGTGAAACATGATGATTCCGANNNNAGATCGGAAGAGCACACGTGACTCGCCAAGCTGAAGNNNNNNNNNNNGAGATGTTAAACAAAATAAGGAGCATA
PF3D7-1371600_	ref	TGTATTTTTCCCACCATTCTTTTCGTNNNNAGATCGGAAGAGCACACGTGACTCGCCAAGCTGAAGNNNNNNNNNNNTTTTCCTATTTTGCACCCATTTATTAT
PF3D7-1371600_	ref	AAAACCTGAATTAGTATCTGCTGCCNNNNAGATCGGAAGAGCACACGTGACTCGCCAAGCTGAAGNNNNNNNNNNNTGTTTACTTTGAGAAACACATATAATGA
PF3D7-1371600_	ref	ATATATATTTTTGATTCGCGACGGTAACGNNNNAGATCGGAAGAGCACACGTGACTCGCCAAGCTGAAGNNNNNNNNNNNCTGTAAAGAAAAACCTGAATTAGTATCT
PF3D7-1371700_	ref	TTGTTGGGTTGTTATATATATTTTTATTAGTAAGTNNNNAGATCGGAAGAGCACACGTGACTCGCCAAGCTGAAGNNNNNNNNNNNTCATTCATTAAGAAGGATAAGGGA
PF3D7-1371700_	ref	TGTATTTTCTTTCTTTTTCATATTTATCAAATGTNNNNAGATCGGAAGAGCACACGTGACTCGCCAAGCTGAAGNNNNNNNNNNNATATTAGTAAAACGCACATCATTTTT
PF3D7-1371700_	ref	TCATGAACTGAACAATTTGATAAACGTNNNNAGATCGGAAGAGCACACGTGACTCGCCAAGCTGAAGNNNNNNNNNNNAAATATTTCAAACCAAGAAGAAAGAA
PF3D7-1371700_	ref	TATTTTCATTTAAATTTGTTATCATATTGTGTGTTNNNNAGATCGGAAGAGCACACGTGACTCGCCAAGCTGAAGNNNNNNNNNNNGAGCATAGGAAGACTGTAAAATTAA
PF3D7-1371700_	ref	GGAGAAGATATAATAATTTTAAAAGAAGGGAAGTANNNNAGATCGGAAGAGCACACGTGACTCGCCAAGCTGAAGNNNNNNNNNNNTATATTTTTATAGAATATATTAATTAATCATGGA
PF3D7-1371700_	ref	TCATTAATGTTATCTAATGAATCCCTACATATNNNNAGATCGGAAGAGCACACGTGACTCGCCAAGCTGAAGNNNNNNNNNNNCATCTTTATTAATGATTTCTGACCTAATT
PF3D7-1371700_	ref	AAAAATACCTATTGATATATGGGTAGAACAANNNNAGATCGGAAGAGCACACGTGACTCGCCAAGCTGAAGNNNNNNNNNNNGAAAATGTGAATATGAAATCTTATGTATAT
PF3D7-1371700_	ref	ATACTTTTATATGTTTTATGATCCTTCAAAACANNNNAGATCGGAAGAGCACACGTGACTCGCCAAGCTGAAGNNNNNNNNNNNTTTATCTGACTCATATAATATTTTATAAAATTTA
PF3D7-1371700_	ref	TTTGTTGTGTCAGATATAAGTAATGAAGATNNNNAGATCGGAAGAGCACACGTGACTCGCCAAGCTGAAGNNNNNNNNNNNTGAATGAATATTATCCAGGAATAACTC
PF3D7-1371700_	ref	TTTCCTTTTTATATAATCAAATACATTTTCACCAANNNNAGATCGGAAGAGCACACGTGACTCGCCAAGCTGAAGNNNNNNNNNNNTATAAATAGGTGTAGATTGAGACAAAT
PF3D7-1371700_	ref	GGAATGTTGGAAAATTGTGTGGAAANNNNAGATCGGAAGAGCACACGTGACTCGCCAAGCTGAAGNNNNNNNNNNNAATGAGATTACATAATGCTGGATTAA
PF3D7-1371700_	ref	TATACAAGGTTCAAATGATTCAAAAGGTTTNNNNAGATCGGAAGAGCACACGTGACTCGCCAAGCTGAAGNNNNNNNNNNNTGGATCTGAACATTTCCATATAAAA
PF3D7-1371800_	ref	AAATATATAGAAAATACGGTTCCTAAAACAAATCCNNNNAGATCGGAAGAGCACACGTGACTCGCCAAGCTGAAGNNNNNNNNNNNGTGTAATAGAAAAATGAAAAACAATGAA
PF3D7-1371800_	ref	AAACCATTACCAGAATATTATACCTTTGAANNNNAGATCGGAAGAGCACACGTGACTCGCCAAGCTGAAGNNNNNNNNNNNGTTTTTAAAAATCATATATGAAAACACATTT
PF3D7-1371800_	ref	GCTTACGTGATGAGAGGATGATAAANNNNAGATCGGAAGAGCACACGTGACTCGCCAAGCTGAAGNNNNNNNNNNNGGGGATCTATTATAAAATAATAAAAAGAAAA
PF3D7-1371800_	ref	CTCTCATCACGTAAGCACACATTAANNNNAGATCGGAAGAGCACACGTGACTCGCCAAGCTGAAGNNNNNNNNNNNGGAACTACCTATTATGGAAAATAAAAAT
PF3D7-1371800_	ref	TGAAAAGAAAAGATCTAAAAGAACTGCTTTNNNNAGATCGGAAGAGCACACGTGACTCGCCAAGCTGAAGNNNNNNNNNNNGAAGACTCAGTAATGGAAAATGAA
PF3D7-1371800_	ref	TTTTTATAGACAATTTAATCCTGTTATACTTTTCTNNNNAGATCGGAAGAGCACACGTGACTCGCCAAGCTGAAGNNNNNNNNNNNGTTCCTATCAAACATGCAAATAAAAA
PF3D7-1371800_	ref	TATATACAAAATGTTGGAAAAGATATTTGATCGTGNNNNAGATCGGAAGAGCACACGTGACTCGCCAAGCTGAAGNNNNNNNNNNNGATAAAATTGAAAAACGTCGATTAAAAA
PF3D7-1371800_	ref	TGTTCATTTCTCTAATATCTTTGTATCTTCTNNNNAGATCGGAAGAGCACACGTGACTCGCCAAGCTGAAGNNNNNNNNNNNGCTGTTCTTTATATTCATCTGATAATT
PF3D7-1371800_	ref	ATTAAATCAATTAAATAAAAAGGATTCCTATAGGANNNNAGATCGGAAGAGCACACGTGACTCGCCAAGCTGAAGNNNNNNNNNNNAAAAATTTATTACTATTGGAAAATGATGAA
PF3D7-1371800_	ref	ATCTTTTATTTCTTTTATATGTTTTAATTGATCCCNNNNAGATCGGAAGAGCACACGTGACTCGCCAAGCTGAAGNNNNNNNNNNNTTCTACTTCATCGAAATATTCTGTATTT
PF3D7-1371800_	ref	TACAAATTATTACTTGATAAAGATGAAGAAGAAAANNNNAGATCGGAAGAGCACACGTGACTCGCCAAGCTGAAGNNNNNNNNNNNTCCCAATAAGTTCAACACCTATTAAA
PF3D7-1371900_	ref	TATATATATTTTGTAAACCTTTCCTCTTCTTTTACNNNNAGATCGGAAGAGCACACGTGACTCGCCAAGCTGAAGNNNNNNNNNNNAAAAATCTTCGTTTGGCTTTTTATT
PF3D7-1371900_	ref	AAATACTTCAATTGTGTATGTGTTTTATTTTCCTNNNNAGATCGGAAGAGCACACGTGACTCGCCAAGCTGAAGNNNNNNNNNNNTGTAAAACTTTCTGCCATACATCT
PF3D7-1371900_	ref	AATTGGTGGAGAAGTAAGTGTTAGTGNNNNAGATCGGAAGAGCACACGTGACTCGCCAAGCTGAAGNNNNNNNNNNNATCAGAAATAATAAAAATAACGGATTAATATT
PF3D7-1371900_	ref	TAAATAAGAAAGTCCTGATACATACCATTTAANNNNAGATCGGAAGAGCACACGTGACTCGCCAAGCTGAAGNNNNNNNNNNNGGATCTTAACTTATCAATTATACCATTAATTA
PF3D7-1371900_	ref	TAGAATTAAATGAAACCTCAGAATAGTAACANNNNAGATCGGAAGAGCACACGTGACTCGCCAAGCTGAAGNNNNNNNNNNNAAATTCCCAATGATAATCATAAATACG
PF3D7-1371900_	ref	CAAGTGATGGTAACAATGAAAATAATACACNNNNAGATCGGAAGAGCACACGTGACTCGCCAAGCTGAAGNNNNNNNNNNNCATAGTTAAACAAGAAAATTATAAAGCATAA
PF3D7-1372000_	ref	TGATGTACGTATCATTTATGGTAATTTATAATTAANNNNAGATCGGAAGAGCACACGTGACTCGCCAAGCTGAAGNNNNNNNNNNNGTAAAAATTATTATAATGAAATGTTATAATGAATT
PF3D7-1372000_	ref	TTAAATTGCTAAATTCATCTAAACATGAAAATAAGNNNNAGATCGGAAGAGCACACGTGACTCGCCAAGCTGAAGNNNNNNNNNNNTTTACTTTTCGTAAATTAATTTTATGTTATTC
PF3D7-1372000_	ref	TCATCTATATTAATCATTGTGATGTTTGATTCNNNNAGATCGGAAGAGCACACGTGACTCGCCAAGCTGAAGNNNNNNNNNNNAAAGAGTATAAAAATGAATTATATGATAATATTAT
PF3D7-1372000_	ref	TCAACATATATATATTACGGAATGAAAAGGAANNNNAGATCGGAAGAGCACACGTGACTCGCCAAGCTGAAGNNNNNNNNNNNATTCAGATAAATTTCTTTCAACCAATGA
PF3D7-1372000_	ref	ATCGGAAGTTGAATCAGAAAATGAATCAGNNNNAGATCGGAAGAGCACACGTGACTCGCCAAGCTGAAGNNNNNNNNNNNATGAATGATGAAAATAATACCTCAGATT
PF3D7-1372000_	ref	AATGATTGGACCATAATAATTTCTGAGTGCNNNNAGATCGGAAGAGCACACGTGACTCGCCAAGCTGAAGNNNNNNNNNNNGTTCAGTTTCATTTTCTGTATCATTTT
PF3D7-1372000_	ref	AAAGGGTGACATTATTAGAAAAACATTAAATANNNNAGATCGGAAGAGCACACGTGACTCGCCAAGCTGAAGNNNNNNNNNNNAATCAGAAAATGAATCAGAAGATCA
PF3D7-1372000_	ref	TCGCCATCAGATTCATTTTGTGATTNNNNAGATCGGAAGAGCACACGTGACTCGCCAAGCTGAAGNNNNNNNNNNNACTATTTAATTGTCTTGTAATATCATTATAATT
PF3D7-1372000_	ref	AAAATTATGAAAAATCGTGTTTGTCTGAATACNNNNAGATCGGAAGAGCACACGTGACTCGCCAAGCTGAAGNNNNNNNNNNNAGATAAGTTTAATGATTCAAATAATAAGTTTAA
PF3D7-1372000_	ref	ATACATTAACTAAATCTTCATTTGGTATATTTTCTNNNNAGATCGGAAGAGCACACGTGACTCGCCAAGCTGAAGNNNNNNNNNNNCATATGATGCATTTAATAATTTCACTTC
PF3D7-1372100_	ref	ATTATTTTATATTGTCCTTTTGGTAGGTTGTANNNNAGATCGGAAGAGCACACGTGACTCGCCAAGCTGAAGNNNNNNNNNNNAAAGGAGTATTATTTTATTTTTCATTATTCTTA
PF3D7-1372100_	ref	TGTTATTTTTATCCTTCCTATATTTCAAAGGATNNNNAGATCGGAAGAGCACACGTGACTCGCCAAGCTGAAGNNNNNNNNNNNTATATTGATACTTATAATAAGAAGAAAAACAA
PF3D7-1372100_	ref	ATGAAGGAATAAAGAAAGAATATGAATTAGATTTCNNNNAGATCGGAAGAGCACACGTGACTCGCCAAGCTGAAGNNNNNNNNNNNGATGATATATTGAGTTATAAAAATATAAAAGAT
PF3D7-1372100_	ref	TGCATCAATTCTTCAATTAAAACATCAAGNNNNAGATCGGAAGAGCACACGTGACTCGCCAAGCTGAAGNNNNNNNNNNNTCCTCTTTCATTTTTCATTACATTCAA
PF3D7-1372100_	ref	TTTTAATGAATGGACTGAGGTCAATTCGNNNNAGATCGGAAGAGCACACGTGACTCGCCAAGCTGAAGNNNNNNNNNNNATTAGATTTCAATATTGAGAAGAACGA
PF3D7-1372100_	ref	AAATCAGTTATTATATGATTAAATTTTCCTCTTTCNNNNAGATCGGAAGAGCACACGTGACTCGCCAAGCTGAAGNNNNNNNNNNNCTATTAATCATAACAAGCATTTTAAATTCT
PF3D7-1372100_	ref	AGTTACAGATAAATACAAGAAGATATATCAAGAGNNNNAGATCGGAAGAGCACACGTGACTCGCCAAGCTGAAGNNNNNNNNNNNCGATTATACATGAAAAATGCAGAAATA
PF3D7-1372100_	ref	TTCGTATTCTCTTCATTTATTTTATCTTGAAATGGNNNNAGATCGGAAGAGCACACGTGACTCGCCAAGCTGAAGNNNNNNNNNNNGTAAAGAATAATGAAATAAACATTTTTATCTAT
PF3D7-1372100_	ref	TAAGAGCTTCATGAGTAGATGTCCTANNNNAGATCGGAAGAGCACACGTGACTCGCCAAGCTGAAGNNNNNNNNNNNATTGTCCTTTTGGTAGGTTGTAAT
PF3D7-1372100_	ref	TTTCCCATCATATTTACATATGTTCTGTAANNNNAGATCGGAAGAGCACACGTGACTCGCCAAGCTGAAGNNNNNNNNNNNTCGTATGACATTTCGTTTAATTCA
PF3D7-1372100_	ref	ATAATTTGAATGAAGAGAATAATATGAAATCAAAGNNNNAGATCGGAAGAGCACACGTGACTCGCCAAGCTGAAGNNNNNNNNNNNGACATGGTTTATGAATCTAGTGTATATTTA
PF3D7-1372100_	ref	ATCATACTCACCTTTGGATGTTTCGANNNNAGATCGGAAGAGCACACGTGACTCGCCAAGCTGAAGNNNNNNNNNNNCTTCCTCAATTAATAATTTGTCAACATT
PF3D7-1372100_	ref	TTATTGAGAGGTATATCAAATAATGAAGTACANNNNAGATCGGAAGAGCACACGTGACTCGCCAAGCTGAAGNNNNNNNNNNNAAAGAAACATTCATTGATTATTATAATGATA
PF3D7-1372100_	ref	TATCTTTGTGGATATATTATCATTTGATTCTTCCNNNNAGATCGGAAGAGCACACGTGACTCGCCAAGCTGAAGNNNNNNNNNNNTCCAATTAGAATCATTTTCTTCATCTA
PF3D7-1372100_	ref	ATTAATATTTCATACGTAAAAGAATACAAACCTAANNNNAGATCGGAAGAGCACACGTGACTCGCCAAGCTGAAGNNNNNNNNNNNAAGAATCAGAAGATATTGAATTATTTAGA
PF3D7-1372100_	ref	TCAATTTGTACAGTATCATTCATATAATCTTTTANNNNAGATCGGAAGAGCACACGTGACTCGCCAAGCTGAAGNNNNNNNNNNNACACATTCTACAATTAGTTTATTAGAAA
PF3D7-1372400_	ref	AACGTTTGTAAATTTTTGTGTTGTATATGGNNNNAGATCGGAAGAGCACACGTGACTCGCCAAGCTGAAGNNNNNNNNNNNTATTAATTAAAAGTGATTCCATGTTTAG
PF3D7-1372400_	ref	ATTATTTAGCATATCCGTTTCTATATATTCACCTTNNNNAGATCGGAAGAGCACACGTGACTCGCCAAGCTGAAGNNNNNNNNNNNATCATTTTCCCCTTTACATAATCAAT
PF3D7-1372400_	ref	TTAAGTGGAAGTAGCACGGGTAGNNNNAGATCGGAAGAGCACACGTGACTCGCCAAGCTGAAGNNNNNNNNNNNTGATGAAACATTAAATGAGACTATTTAT
PF3D7-1372400_	ref	TTCATATTTCTTTTTAACTGCATCTATGTAAAAGGNNNNAGATCGGAAGAGCACACGTGACTCGCCAAGCTGAAGNNNNNNNNNNNACTCCCTGAGTTATGTTCTTCT
PF3D7-1372400_	ref	AAGAGAAGGATCATTATTAAATAGTAATGAAAGAGNNNNAGATCGGAAGAGCACACGTGACTCGCCAAGCTGAAGNNNNNNNNNNNAAATGGAAGTGATAATGAAAATAAAAGA
PF3D7-1372400_	ref	TTCTTCTTAATTGTTTTTGTACTCCCTGNNNNAGATCGGAAGAGCACACGTGACTCGCCAAGCTGAAGNNNNNNNNNNNTTTTCATGATTCATCCTTTATTCCA
hrp2_S0_Sub0_m	alt0	CACGAAGCCGCCACACATTNNNNAGATCGGAAGAGCACACGTGACTCGCCAAGCTGAAGNNNNNNNNNNNCAACCGATGCTCATCATGCAG
hrp2_S0_Sub0_m	alt1	CACGAAGCCGCCACACATTNNNNAGATCGGAAGAGCACACGTGACTCGCCAAGCTGAAGNNNNNNNNNNNCATCCGATGCTCATCATGCAG
hrp2_S0_Sub0_m	alt2	CACCATGCAGCCGCACACNNNNAGATCGGAAGAGCACACGTGACTCGCCAAGCTGAAGNNNNNNNNNNNCAGCTGATGCTCATCATGCAG
hrp2_S0_Sub0_m	alt3	CACGAAGCCGCCACACATTNNNNAGATCGGAAGAGCACACGTGACTCGCCAAGCTGAAGNNNNNNNNNNNGCTGATGCTCACCATGCAG
hrp2_S0_Sub0_m	ref	cacgaAGcCGcCACACATTGNNNNAGATCGGAAGAGCACACGTGACTCGCCAAGCTGAAGNNNNNNNNNNNaaccgatgctcatcatgcag
hrp2_S0_Sub0_m	alt0	GATGGGCATCGGCTGCANNNNAGATCGGAAGAGCACACGTGACTCGCCAAGCTGAAGNNNNNNNNNNNGCTGCATGGTGAGCATCG
hrp2_S0_Sub0_m	alt1	GATGGGCATCGGCTGCANNNNAGATCGGAAGAGCACACGTGACTCGCCAAGCTGAAGNNNNNNNNNNNTGCATGATGAGCATCGGCT
hrp2_S0_Sub0_m	alt2	GCTGCATGATGAGCATCAGCNNNNAGATCGGAAGAGCACACGTGACTCGCCAAGCTGAAGNNNNNNNNNNNTGCATGGTGAGCATCAGTT
hrp2_S0_Sub0_m	alt3	GCTGCATGATGAGCATCGGANNNNAGATCGGAAGAGCACACGTGACTCGCCAAGCTGAAGNNNNNNNNNNNTGCATGGTGAGCATCAGTT
hrp2_S0_Sub0_m	ref	gctgcatgatgagcatcggatNNNNAGATCGGAAGAGCACACGTGACTCGCCAAGCTGAAGNNNNNNNNNNNctgcatggtgagcatcagtt
hrp2_S0_Sub0_m	ref	catcggctgcatggtgagcatNNNNAGATCGGAAGAGCACACGTGACTCGCCAAGCTGAAGNNNNNNNNNNNctgcatggtgagcatcagtt
hrp2_S0_Sub0_m	alt0	ATGCAGCTGATGCTCATCATGCNNNNAGATCGGAAGAGCACACGTGACTCGCCAAGCTGAAGNNNNNNNNNNNGCTCATCATGCAGCCGAT
hrp2_S0_Sub0_m	alt1	ATGCTCATCACGCTCACCATNNNNAGATCGGAAGAGCACACGTGACTCGCCAAGCTGAAGNNNNNNNNNNNGCTCACCATGCAGCTGAT
hrp2_S0_Sub0_m	alt2	ATGCTCATCATGCAGCCGATNNNNAGATCGGAAGAGCACACGTGACTCGCCAAGCTGAAGNNNNNNNNNNNGCTCATCATGCAGCCGAT
hrp2_S0_Sub0_m	alt3	ATGCTCATCACGCTCACCATNNNNAGATCGGAAGAGCACACGTGACTCGCCAAGCTGAAGNNNNNNNNNNNGCTCATCATGCAGCCGAT
hrp2_S0_Sub0_m	alt4	ATGCTCATCACGCTCACCATNNNNAGATCGGAAGAGCACACGTGACTCGCCAAGCTGAAGNNNNNNNNNNNTGCTCATCATGCATCCGAT
hrp2_S0_Sub0_m	alt5	ATGCTCATCACGCTCACCATNNNNAGATCGGAAGAGCACACGTGACTCGCCAAGCTGAAGNNNNNNNNNNNTGCTCATCATGCAGCCTAT
hrp2_S0_Sub0_m	ref	atgctcatcacgctcaccatgNNNNAGATCGGAAGAGCACACGTGACTCGCCAAGCTGAAGNNNNNNNNNNNatgctcatcatgcagcctat
hrp2_S0_Sub0_m	alt0	CGATGCTCACCATGCAACCGATNNNNAGATCGGAAGAGCACACGTGACTCGCCAAGCTGAAGNNNNNNNNNNNTGCCCATCATGCTCATCAT
hrp2_S0_Sub0_m	alt1	CGATGCTCACCATGCAACCGATNNNNAGATCGGAAGAGCACACGTGACTCGCCAAGCTGAAGNNNNNNNNNNNGCCCATCATGCTCACCAT
hrp2_S0_Sub0_m	alt10	CGATGGCTCATCATGCAGCCGNNNNAGATCGGAAGAGCACACGTGACTCGCCAAGCTGAAGNNNNNNNNNNNTGCTCATCACGCTCATCAT
hrp2_S0_Sub0_m	alt11	CGATGCTCATCATGCAGCCGATNNNNAGATCGGAAGAGCACACGTGACTCGCCAAGCTGAAGNNNNNNNNNNNTGCTCATCACGCTCATCAT
hrp2_S0_Sub0_m	alt12	TGATGCTCATCATGCAGCTGATGCNNNNAGATCGGAAGAGCACACGTGACTCGCCAAGCTGAAGNNNNNNNNNNNTGCTCATCACGCTCATCAT
hrp2_S0_Sub0_m	alt2	CGATGCTCACCATGCAACCGATNNNNAGATCGGAAGAGCACACGTGACTCGCCAAGCTGAAGNNNNNNNNNNNTGCTCATCACGCTCATCAT
hrp2_S0_Sub0_m	alt3	GATGCTCATCATGCAGCCGCNNNNAGATCGGAAGAGCACACGTGACTCGCCAAGCTGAAGNNNNNNNNNNNGCTCATCATGCATCCGATG
hrp2_S0_Sub0_m	alt4	CGATGCTCACCATGCAGCCGNNNNAGATCGGAAGAGCACACGTGACTCGCCAAGCTGAAGNNNNNNNNNNNTGCTCATCACGCTCATCAT
hrp2_S0_Sub0_m	alt5	CGATGCTCATCATGCAGCCGATNNNNAGATCGGAAGAGCACACGTGACTCGCCAAGCTGAAGNNNNNNNNNNNTGCCCATCATGCTCATCAT
hrp2_S0_Sub0_m	alt6	CGATGCTCACCATGCAACCGNNNNAGATCGGAAGAGCACACGTGACTCGCCAAGCTGAAGNNNNNNNNNNNTGCTCATCACGCTCATCAT
hrp2_S0_Sub0_m	alt7	TTATGCCCATCACGCTCATCATGCNNNNAGATCGGAAGAGCACACGTGACTCGCCAAGCTGAAGNNNNNNNNNNNTGCTCATCACGCTCATCAT
hrp2_S0_Sub0_m	alt8	TTATGCCCATCACGCTCATCATGCNNNNAGATCGGAAGAGCACACGTGACTCGCCAAGCTGAAGNNNNNNNNNNNGCTCATCACGCTCACCAT
hrp2_S0_Sub0_m	alt9	CTCACCATGCAGCCGATGCNNNNAGATCGGAAGAGCACACGTGACTCGCCAAGCTGAAGNNNNNNNNNNNTGCCCATCATGCTCATCAT
hrp2_S0_Sub0_m	ref	cgatgctcaccatgcaaccgaNNNNAGATCGGAAGAGCACACGTGACTCGCCAAGCTGAAGNNNNNNNNNNNatgctcatcacgctcatcat
hrp2_S0_Sub0_m	alt0	ATGCTCATCATGCAGCCGATNNNNAGATCGGAAGAGCACACGTGACTCGCCAAGCTGAAGNNNNNNNNNNNTGCCCATCATGCTCATCAT
hrp2_S0_Sub0_m	alt1	ACGCTCATCATGCAGCCGATNNNNAGATCGGAAGAGCACACGTGACTCGCCAAGCTGAAGNNNNNNNNNNNTGCCCATCATGCTCATCAT
hrp2_S0_Sub0_m	alt2	ATGCTCACCATGCAGCTGATNNNNAGATCGGAAGAGCACACGTGACTCGCCAAGCTGAAGNNNNNNNNNNNTGCCCATCATGCTCATCAT
hrp2_S0_Sub0_m	alt3	TGCATCCGATGCTCATCATGCANNNNAGATCGGAAGAGCACACGTGACTCGCCAAGCTGAAGNNNNNNNNNNNCTCATCATGCAGCCGATG
hrp2_S0_Sub0_m	alt4	ACGCTCATCATGCAGCCGATNNNNAGATCGGAAGAGCACACGTGACTCGCCAAGCTGAAGNNNNNNNNNNNTGCTCATCATGCTCACCAT
hrp2_S0_Sub0_m	alt5	ACGCTCACCATGCAGCCGNNNNAGATCGGAAGAGCACACGTGACTCGCCAAGCTGAAGNNNNNNNNNNNTGCCCATCATGCTCATCAT
hrp2_S0_Sub0_m	alt6	ATGCTCATCATGCAGCCTATGCNNNNAGATCGGAAGAGCACACGTGACTCGCCAAGCTGAAGNNNNNNNNNNNTGCCCATCATGCTCATCAT
hrp2_S0_Sub0_m	alt7	ATGCTCATCATGCATCCGATGCNNNNAGATCGGAAGAGCACACGTGACTCGCCAAGCTGAAGNNNNNNNNNNNGCCCATCACGCTCATCAT
hrp2_S0_Sub0_m	ref	acgctcatcatgcatccgatgNNNNAGATCGGAAGAGCACACGTGACTCGCCAAGCTGAAGNNNNNNNNNNNatgcccatcatgctcatcat
hrp2_S0_Sub0_m	alt0	CATCGGCTGCATGATGAGCGTGNNNNAGATCGGAAGAGCACACGTGACTCGCCAAGCTGAAGNNNNNNNNNNNNATGATGAGCATGATGGGCATAG
hrp2_S0_Sub0_m	alt1	CATCGGCTGCATGATGAGCATGNNNNAGATCGGAAGAGCACACGTGACTCGCCAAGCTGAAGNNNNNNNNNNNNATGAGCATGATGGGCATCG
hrp2_S0_Sub0_m	alt10	CATCAGCTGCATGGTGAGCATGATNNNNAGATCGGAAGAGCACACGTGACTCGCCAAGCTGAAGNNNNNNNNNNNTGATGAGCGTGATGGGCATAA
hrp2_S0_Sub0_m	alt11	CATCGGTTGCATGGTGAGCATCNNNNAGATCGGAAGAGCACACGTGACTCGCCAAGCTGAAGNNNNNNNNNNNNATGATGAGCATGATGGGCATAG
hrp2_S0_Sub0_m	alt12	CATCGGCTGCATGATGAGCGTGNNNNAGATCGGAAGAGCACACGTGACTCGCCAAGCTGAAGNNNNNNNNNNNNGTGAGCATGATGGGCATCG
hrp2_S0_Sub0_m	alt13	CATCGGATGCATGATGAGCATGATNNNNAGATCGGAAGAGCACACGTGACTCGCCAAGCTGAAGNNNNNNNNNNNNGTGAGCGTGATGAGCATCG
hrp2_S0_Sub0_m	alt2	CATCGGCTGCATGGTGAGCGNNNNAGATCGGAAGAGCACACGTGACTCGCCAAGCTGAAGNNNNNNNNNNNNATGAGCATGATGGGCATCG
hrp2_S0_Sub0_m	alt3	CATCGGCTGCATGGTGAGCGNNNNAGATCGGAAGAGCACACGTGACTCGCCAAGCTGAAGNNNNNNNNNNNNATGATGAGCATGATGGGCATAG
hrp2_S0_Sub0_m	alt4	CATCGGCTGCATGATGAGCGTGNNNNAGATCGGAAGAGCACACGTGACTCGCCAAGCTGAAGNNNNNNNNNNNTGATGAGCGTGATGGGCATAA
hrp2_S0_Sub0_m	alt5	CATCGGCTGCATGATGAGCATCNNNNAGATCGGAAGAGCACACGTGACTCGCCAAGCTGAAGNNNNNNNNNNNNATGATGAGCATGATGGGCATAG
hrp2_S0_Sub0_m	alt6	CATAGGCTGCATGATGAGCATGATNNNNAGATCGGAAGAGCACACGTGACTCGCCAAGCTGAAGNNNNNNNNNNNNATGATGAGCATGATGGGCATAG
hrp2_S0_Sub0_m	alt7	CATCGGCTGCATGATGAGCATCNNNNAGATCGGAAGAGCACACGTGACTCGCCAAGCTGAAGNNNNNNNNNNNTGATGAGCGTGATGAGCATCA
hrp2_S0_Sub0_m	alt8	CATCGGCTGCATGATGAGCATGNNNNAGATCGGAAGAGCACACGTGACTCGCCAAGCTGAAGNNNNNNNNNNNTGATGAGCGTGATGAGCATCA
hrp2_S0_Sub0_m	alt9	CATCGGCTGCATGATGAGCATGNNNNAGATCGGAAGAGCACACGTGACTCGCCAAGCTGAAGNNNNNNNNNNNTGATGAGCGTGATGGGCATAA
hrp2_S0_Sub0_m	ref	CATcgGctgcatgatgagcgtNNNNAGATCGGAAGAGCACACGTGACTCGCCAAGCTGAAGNNNNNNNNNNNgatgagcgtgatgggcataa
hrp2_S0_Sub0_m	alt0	ATCATCTACATGTGCTTGAGTTTCGTNNNNAGATCGGAAGAGCACACGTGACTCGCCAAGCTGAAGNNNNNNNNNNNTGGTGAGCATGATGAGCAT
hrp2_S0_Sub0_m	alt1	ATCATCTACATGTGCTTGAGTTTCGTNNNNAGATCGGAAGAGCACACGTGACTCGCCAAGCTGAAGNNNNNNNNNNNTGCATGGTGAGCATGATGG
hrp2_S0_Sub0_m	ref	ATCATCTACATGTGCTTGAGTTTCGNNNNAGATCGGAAGAGCACACGTGACTCGCCAAGCTGAAGNNNNNNNNNNNatggtgagcatgatgagcat
hrp2_S0_Sub0_m	alt0	ATGCTCATCATGCAGCCGATNNNNAGATCGGAAGAGCACACGTGACTCGCCAAGCTGAAGNNNNNNNNNNNCGCATTTAATAATAACTTGTGTAGCAAAA
hrp2_S0_Sub0_m	alt1	ATGCTCCATCATGCAGCCGATNNNNAGATCGGAAGAGCACACGTGACTCGCCAAGCTGAAGNNNNNNNNNNNCGCATTTAATAATAACTTGTGTAGCAAAA
hrp2_S0_Sub0_m	alt2	ATGCTCATCATGTAGCCGATGCNNNNAGATCGGAAGAGCACACGTGACTCGCCAAGCTGAAGNNNNNNNNNNNCGCATTTAATAATAACTTGTGTAGCAAAA
hrp2_S0_Sub0_m	alt3	ATGCTCATCATGCAGCCTATGCNNNNAGATCGGAAGAGCACACGTGACTCGCCAAGCTGAAGNNNNNNNNNNNCGCATTTAATAATAACTTGTGTAGCAAAA
hrp2_S0_Sub0_m	alt4	ACGCTCATCATGCAGCCGATNNNNAGATCGGAAGAGCACACGTGACTCGCCAAGCTGAAGNNNNNNNNNNNCGCATTTAATAATAACTTGTGTAGCAAAA
hrp2_S0_Sub0_m	alt5	TGCAGCCGATGCCCATCATNNNNAGATCGGAAGAGCACACGTGACTCGCCAAGCTGAAGNNNNNNNNNNNACTTGTGTAGCAAAAATGCAAAAGG
hrp2_S0_Sub0_m	alt6	ACGCTCATCATGCCAGCCGNNNNAGATCGGAAGAGCACACGTGACTCGCCAAGCTGAAGNNNNNNNNNNNCGCATTTAATAATAACTTGTGTAGCAAAA
hrp2_S0_Sub0_m	alt7	ATGCTCACCATGCAGCCGATNNNNAGATCGGAAGAGCACACGTGACTCGCCAAGCTGAAGNNNNNNNNNNNCGCATTTAATAATAACTTGTGTAGCAAAA
hrp2_S0_Sub0_m	ref	atgctcatcatgcagccgatgNNNNAGATCGGAAGAGCACACGTGACTCGCCAAGCTGAAGNNNNNNNNNNNGcATTTAATAATAACTTGTGTAGCAAAA
hrp3_S0_Sub0_m	ref	tcgtggtgtgctccatcatggNNNNAGATCGGAAGAGCACACGTGACTCGCCAAGCTGAAGNNNNNNNNNNNGATTCATCATTCTATATTTACATGGaAGA
hrp3_S0_Sub0_m	ref	TTTTGCTTCCGTACTTTTGTTAGATAACGNNNNAGATCGGAAGAGCACACGTGACTCGCCAAGCTGAAGNNNNNNNNNNNAATGCTTATTTTTcACATTTTCATGAA
hrp3_S0_Sub0_m	alt0	GTGAGAATCATCAAAACAAGCATTAGCTNNNNAGATCGGAAGAGCACACGTGACTCGCCAAGCTGAAGNNNNNNNNNNNTAATGGTGTAAGTGATGCGTAGT
hrp3_S0_Sub0_m	ref	GTGAGAATCATCAAAACAaGCATTAGCNNNNAGATCGGAAGAGCACACGTGACTCGCCAAGCTGAAGNNNNNNNNNNNAATGGTGTAaGTGATGcGTAGT
hrp3_S0_Sub0_m	alt0	ACACCATGATGGAGCACACCATNNNNAGATCGGAAGAGCACACGTGACTCGCCAAGCTGAAGNNNNNNNNNNNATCACGGATTTCATTTTAACCTTCA
hrp3_S0_Sub0_m	ref	acaccatGATGGAGCACACCANNNNAGATCGGAAGAGCACACGTGACTCGCCAAGCTGAAGNNNNNNNNNNNTCACGGATTTCATTTTAACCTTCA
hrp3_S0_Sub0_m	alt0	CATTAGCTGCATGGTGAGCATTANNNNAGATCGGAAGAGCACACGTGACTCGCCAAGCTGAAGNNNNNNNNNNNTTGCATGATGTAAAGTATGGGAATT
hrp3_S0_Sub0_m	alt1	CATTAGCTGCATGATGAGCATTAGCNNNNAGATCGGAAGAGCACACGTGACTCGCCAAGCTGAAGNNNNNNNNNNNTTGCATGATGTAAAGTATGGGAATT
hrp3_S0_Sub0_m	alt2	CATCAGCTACATGATGAGCATCANNNNAGATCGGAAGAGCACACGTGACTCGCCAAGCTGAAGNNNNNNNNNNNTTGCATGATGTAAAGTATGGGAATT
hrp3_S0_Sub0_m	alt3	GATGTGCATGATGGGCATCAGNNNNAGATCGGAAGAGCACACGTGACTCGCCAAGCTGAAGNNNNNNNNNNNATGGTGAGCATTAGCTGCATG
hrp3_S0_Sub0_m	alt4	CATCAGCTACATGATGTGCATGATGNNNNAGATCGGAAGAGCACACGTGACTCGCCAAGCTGAAGNNNNNNNNNNNTTGCATGATGTAAAGTATGGGAATT
hrp3_S0_Sub0_m	alt5	CATTAGCTGCATGGTGAGCATNNNNAGATCGGAAGAGCACACGTGACTCGCCAAGCTGAAGNNNNNNNNNNNTTGCATGATGTAAAGTATGGGAATT
hrp3_S0_Sub0_m	ref	cattagctgcatggtgagcattagNNNNAGATCGGAAGAGCACACGTGACTCGCCAAGCTGAAGNNNNNNNNNNNTGCATGATGTAAAGTATGGGAATT
hrp3_S0_Sub0_m	alt0	CTAATGCTCACCATGCAGCTAATNNNNAGATCGGAAGAGCACACGTGACTCGCCAAGCTGAAGNNNNNNNNNNNGCCCATCATGCACATCAT
hrp3_S0_Sub0_m	alt1	CTCACCATGCAGCTAATGCTCANNNNAGATCGGAAGAGCACACGTGACTCGCCAAGCTGAAGNNNNNNNNNNNCATGCAGGTGATGCCCAT
hrp3_S0_Sub0_m	alt2	CTAATGCTCACCATGCAGCTAATNNNNAGATCGGAAGAGCACACGTGACTCGCCAAGCTGAAGNNNNNNNNNNNAAAGTCAAGCACATGCAGG
hrp3_S0_Sub0_m	ref	ctaatgctCAcCATGCAGCTAATGNNNNAGATCGGAAGAGCACACGTGACTCGCCAAGCTGAAGNNNNNNNNNNNatgcccatcatgcacatcat
hrp3_S0_Sub0_m	alt0	TTTTGCTAAACAAGTTATTGTTAAATTCGGANNNNAGATCGGAAGAGCACACGTGACTCGCCAAGCTGAAGNNNNNNNNNNNATTAGCTGCATGGTGAGCATT
hrp3_S0_Sub0_m	alt1	TTTTGCTAAACAAGTTATTGTTAAATTCGGANNNNAGATCGGAAGAGCACACGTGACTCGCCAAGCTGAAGNNNNNNNNNNNCATTAGCTGCATGATGAGCATT
hrp3_S0_Sub0_m	alt2	GTCCTTTTGCATTTTTGCTAAACAAGTTANNNNAGATCGGAAGAGCACACGTGACTCGCCAAGCTGAAGNNNNNNNNNNNCATTAGCTGCATGATGAGCATT
hrp3_S0_Sub0_m	alt3	GTCCTTTTGCATTTTTGCTAAACAAGTTANNNNAGATCGGAAGAGCACACGTGACTCGCCAAGCTGAAGNNNNNNNNNNNCTCCATCATGGTGGGCATC
hrp3_S0_Sub0_m	alt4	GTCCTTTTGCATTTTTGCTAAACAAGTTANNNNAGATCGGAAGAGCACACGTGACTCGCCAAGCTGAAGNNNNNNNNNNNATTAGCTGCATGGTGAGCATT
hrp3_S0_Sub0_m	ref	GTCCTTTTGCATTTTTGCtAAAcAAGTTNNNNAGATCGGAAGAGCACACGTGACTCGCCAAGCTGAAGNNNNNNNNNNNttagctgcatggtgagcatt
hrp3_S0_Sub0_m	alt0	TCAAATAAGAGATTATTACACGAAAGTCANNNNAGATCGGAAGAGCACACGTGACTCGCCAAGCTGAAGNNNNNNNNNNNTTGCTTCCGTACTTTTGTTAGATAA
hrp3_S0_Sub0_m	alt1	TTATTACACGAAAGTCAAGCACATGCANNNNAGATCGGAAGAGCACACGTGACTCGCCAAGCTGAAGNNNNNNNNNNNTTGCTTCCGTACTTTTGTTAGATAA
hrp3_S0_Sub0_m	ref	TTATTACACGAAAGTCAAGCACATgcNNNNAGATCGGAAGAGCACACGTGACTCGCCAAGCTGAAGNNNNNNNNNNNTGCTTCCGTACTTTTGTTAGATAA
hrp3_S0_Sub0_m	alt0	GAAGCAAAAACGGCAGCGGATANNNNAGATCGGAAGAGCACACGTGACTCGCCAAGCTGAAGNNNNNNNNNNNTTTGCTAAACAAGTTATTGTTAAATT
hrp3_S0_Sub0_m	alt1	GAAGCAAAAACGGCAGCGGATANNNNAGATCGGAAGAGCACACGTGACTCGCCAAGCTGAAGNNNNNNNNNNNTTTTGCATTTTTGCTAAACAAGT
hrp3_S0_Sub0_m	alt2	GAAGCAAAAACGGCAGCGGATANNNNAGATCGGAAGAGCACACGTGACTCGCCAAGCTGAAGNNNNNNNNNNNAAGTTATTGTTAAATTCGGAGTTATT
hrp3_S0_Sub0_m	ref	GAAGCAAAAACGGCAGCGGATAANNNNAGATCGGAAGAGCACACGTGACTCGCCAAGCTGAAGNNNNNNNNNNNCTTTTGCATTTTTGCtAAAcAAGT

Supplementary Table 7A. Unique molecular identifier (UMI) counts by sample and locus. **Absolute coverage.**

MIP	PF3D7-1370500_S0_Sub0_mip2	PF3D7-1370500_S0_Sub0_mip3	PF3D7-1370500_S0_Sub0_mip4	PF3D7-1370500_S0_Sub0_mip5	PF3D7-1370500_S0_Sub0_mip6	PF3D7-1370900_S0_Sub0_mip0	PF3D7-1370900_S0_Sub0_mip1	PF3D7-1371000_S0_Sub0_mip0
Chrom	chr11	chr11	chr11	chr11	chr11	chr11	chr11	chr11
begin	1920280	1920386	1920588	1920716	1920809	1923308	1923404	1928534
end	1920462	1920590	1920760	1920884	1921024	1923492	1923648	1928747
Gene	PF3D7-1370500	PF3D7-1370500	PF3D7-1370500	PF3D7-1370500	PF3D7-1370500	PF3D7-1370900	PF3D7-1370900	PF3D7-1371000
Sample ID								
6	0	3	0	0	0	8	0	2
52	0	8	0	0	0	17	2	15
141	0	3	14	19	17	28	2	26
185	1	0	0	0	0	15	1	14
228	3	0	0	0	0	44	4	34
311	0	1	0	0	0	4	3	21
342	0	2	0	0	0	10	5	25
386	0	0	0	0	0	0	0	1
419	30	0	0	0	0	191	19	182
424	2	692	0	0	0	2325	285	2069
447	0	334	0	0	0	590	60	412
451	1	7	0	0	0	25	3	10
476	0	21	0	0	0	65	3	1
485	1	0	6	4	9	9	0	12
488	13	40	0	0	0	153	9	118
500	56	109	0	0	0	363	41	313
505	168	0	0	0	0	1166	135	623
517	34	0	50	51	0	141	13	128
522	2	1	0	0	0	13	0	12
530	0	9	23	16	0	54	4	55
535	1	0	81	70	147	247	31	150
558	0	0	5	8	15	18	0	0
560	2	0	26	17	56	47	7	24
584	0	0	0	0	0	13	1	4
598	0	9	0	0	0	14	0	18
628	0	0	8	11	15	21	4	0
718	1	184	0	0	0	333	25	231

PF3D7-1370500_S0_Sub0_mip4	PF3D7-1370500_S0_Sub0_mip5	PF3D7-1370500_S0_Sub0_mip6	PF3D7-1370900_S0_Sub0_mip0	PF3D7-1370900_S0_Sub0_mip1	PF3D7-1370900_S0_Sub0_mip3	PF3D7-1370900_S0_Sub0_mip5	PF3D7-1371000_S0_Sub0_mip0	PF3D7-1371000_S0_Sub0_mip1	PF3D7-1371000_S0_Sub0_mip3	PF3D7-1371000_S0_Sub0_mip2	PF3D7-1371000_S0_Sub0_mip4	PF3D7-1371000_S0_Sub0_mip7	PF3D7-1371000_S0_Sub0_mip9
chr13	chr13	chr13	chr13	chr13	chr13	chr13	chr13	chr13	chr13	chr13	chr13	chr13	chr13
2794590	2794718	2794812	2797288	2797384	2797866	2798027	2802545	2802681	2802887	2802890	2803077	2803604	2803985
2794763	2794887	2795027	2797472	2797578	2798036	2798194	2802759	2802868	2803071	2803101	2803279	2803824	2804201
PF3D7-1370500	PF3D7-1370500	PF3D7-1370500	PF3D7-1370900	PF3D7-1370900	PF3D7-1370900	PF3D7-1370900	PF3D7-1371000	PF3D7-1371000	PF3D7-1371000	PF3D7-1371000	PF3D7-1371000	PF3D7-1371000	PF3D7-1371000
3	4	8	0	4	10	2	0	0	7	5	0	2	10
19	19	27	0	5	19	4	0	0	23	7	0	12	22
23	24	43	0	7	29	1	1	0	19	11	0	61	29
7	12	24	0	4	5	0	0	0	6	7	0	8	8
43	49	71	0	6	43	0	19	0	28	16	0	56	76
18	35	5	1	1	45	0	0	0	2	0	1	4	74
27	35	42	0	2	46	0	0	0	9	1	0	7	54
5	6	9	0	0	15	0	0	0	0	0	0	0	19
126	152	273	1	28	106	14	1	0	183	89	1	6	206
1785	1847	3973	9	531	1478	60	0	0	2060	1048	0	1510	2270
319	262	720	0	152	298	110	0	0	392	250	0	0	383
10	17	39	0	7	18	0	0	0	23	14	0	18	16
41	34	118	0	33	40	36	54	23	70	41	0	128	45
1	1	8	7	4	9	1	0	0	9	0	0	20	5
75	94	201	0	23	91	2	1	0	106	65	1	74	115
289	240	610	0	74	196	3	0	0	276	149	0	213	417
883	916	1989	0	209	768	2	437	2	334	204	0	689	1365
49	31	208	1	43	81	6	0	0	133	68	1	92	111
10	13	30	0	2	20	0	0	0	9	5	0	8	16
17	16	67	0	11	41	1	0	0	24	16	0	19	67
61	90	172	1	67	155	6	1	0	165	81	0	104	201

PF3D7-1371000_S0_Sub0_mip10	PF3D7-1371000_S0_Sub0_mip13	PF3D7-1371000_S0_Sub0_mip16	PF3D7-1371000_S0_Sub0_mip17	PF3D7-1371000_S0_Sub0_mip18	PF3D7-1371000_S0_Sub0_mip19	PF3D7-1371000_S0_Sub0_mip20	PF3D7-1371000_S0_Sub0_mip22	PF3D7-1371500_S0_Sub0_mip0	PF3D7-1371500_S0_Sub0_mip1	PF3D7-1371500_S0_Sub0_mip2	PF3D7-1371500_S0_Sub0_mip3	PF3D7-1371500_S0_Sub0_mip4	PF3D7-1371600_S0_Sub0_mip0
chr13	chr13	chr13	chr13	chr13	chr13	chr13	chr13	chr13	chr13	chr13	chr13	chr13	chr13
2804128	2804733	2805291	2805378	2805781	2806111	2806117	2806917	2808585	2808671	2808809	2808964	2809086	2811767
2804329	2804952	2805504	2805597	2805997	2806300	2806319	2807127	2808762	2808855	2808983	2809134	2809265	2811979
PF3D7-1371000	PF3D7-1371000	PF3D7-1371000	PF3D7-1371000	PF3D7-1371000	PF3D7-1371000	PF3D7-1371000	PF3D7-1371000	PF3D7-1371500	PF3D7-1371500	PF3D7-1371500	PF3D7-1371500	PF3D7-1371500	PF3D7-1371600
11	10	0	0	3	6	3	0	0	0	0	0	0	0
33	8	0	1	11	12	9	0	0	0	0	0	0	0
110	23	0	0	74	53	26	0	1	6	9	5	4	21
25	6	0	0	14	15	5	0	0	0	0	0	0	0
199	50	113	0	178	70	33	0	0	0	0	0	0	0
253	30	3	0	167	55	13	0	0	0	0	0	0	0
206	34	0	0	114	74	35	0	0	0	0	0	0	0
74	15	0	0	64	9	5	0	0	0	1	0	0	1
373	143	389	1	253	239	110	1	0	0	0	0	0	0
4140	2264	0	0	2694	2425	1260	0	0	0	0	0	0	0
709	540	0	0	409	450	232	0	0	0	0	0	0	0
35	16	1	0	16	20	8	0	0	0	0	0	0	0
110	57	129	0	69	59	28	1	0	0	0	0	0	0
14	12	0	0	14	11	6	0	0	0	0	0	0	0
232	86	0	0	188	161	68	1	26	31	33	63	27	62
657	449	354	3	419	415	224	0	0	0	0	0	0	0
2147	1193	0	0	1515	1359	696	0	135	197	421	444	102	663
223	94	265	0	157	156	61	0	0	0	0	0	0	0
24	11	32	0	25	28	17	0	0	0	0	0	0	0
124	40	112	0	66	74	32	0	0	0	0	0	0	0
368	150	4	0	258	229	102	2	0	0	0	0	0	0

PF3D7-1371600_S0_Sub0_mip2	PF3D7-1371600_S0_Sub0_mip3	PF3D7-1371600_S0_Sub0_mip4	PF3D7-1371600_S0_Sub0_mip5	PF3D7-1371600_S0_Sub0_mip6	PF3D7-1371600_S0_Sub0_mip7	PF3D7-1371600_S0_Sub0_mip9	PF3D7-1371600_S0_Sub0_mip10	PF3D7-1371600_S0_Sub0_mip20	PF3D7-1371600_S0_Sub0_mip22	PF3D7-1371600_S0_Sub0_mip24	PF3D7-1371600_S0_Sub0_mip28	PF3D7-1371600_S0_Sub0_mip30
chr13	chr13	chr13	chr13	chr13	chr13	chr13	chr13	chr13	chr13	chr13	chr13	chr13
2811977	2812266	2812420	2812590	2812837	2813009	2813244	2813421	2814557	2814849	2815114	2815719	2816090
2812173	2812441	2812618	2812799	2813043	2813224	2813443	2813614	2814756	2815025	2815330	2815912	2816295
PF3D7-1371600	PF3D7-1371600	PF3D7-1371600	PF3D7-1371600	PF3D7-1371600	PF3D7-1371600	PF3D7-1371600	PF3D7-1371600	PF3D7-1371600	PF3D7-1371600	PF3D7-1371600	PF3D7-1371600	PF3D7-1371600
0	0	0	0	0	0	0	0	0	0	0	0	0
0	0	0	0	0	0	0	0	0	0	0	0	0
12	24	25	20	21	12	26	41	25	28	10	30	27
0	0	0	0	0	0	0	0	0	0	0	0	0
0	0	0	0	0	0	0	0	0	0	0	0	0
0	0	0	0	0	0	0	0	0	0	0	0	0
0	0	0	0	0	0	0	0	0	0	0	0	0
0	9	6	0	5	0	9	18	10	4	6	9	14
0	0	0	0	0	0	0	0	0	0	0	0	0
0	0	0	0	0	0	0	0	0	0	0	0	0
0	0	0	0	0	0	0	0	0	0	0	0	0
0	0	0	0	0	0	0	0	0	0	0	0	0
0	0	0	0	0	0	0	0	0	0	0	0	0
0	0	0	0	0	0	0	0	0	0	0	0	0
45	142	74	72	98	74	81	85	70	76	33	47	57
0	0	0	0	0	0	0	0	0	0	0	0	0
363	1010	694	618	909	546	719	976	775	762	290	615	747
0	0	0	0	0	0	0	0	0	0	0	0	0
0	0	0	0	0	0	0	0	0	0	0	0	0
0	0	0	0	0	0	0	0	0	0	0	0	0
0	0	0	0	0	0	0	0	0	0	0	0	0

PF3D7-1371600_S0_Sub0_mip33	PF3D7-1371600_S0_Sub0_mip42	PF3D7-1371600_S0_Sub0_mip43	PF3D7-1371600_S0_Sub0_mip44	PF3D7-1371600_S0_Sub0_mip45	PF3D7-1371600_S0_Sub0_mip46	PF3D7-1371600_S0_Sub0_mip50	PF3D7-1371700_S0_Sub0_mip0	PF3D7-1371700_S0_Sub0_mip2	PF3D7-1371700_S0_Sub0_mip3	PF3D7-1371700_S0_Sub0_mip4	PF3D7-1371700_S0_Sub0_mip5	PF3D7-1371700_S0_Sub0_mip6	PF3D7-1371700_S0_Sub0_mip7	PF3D7-1371700_S0_Sub0_mip8	PF3D7-1371700_S0_Sub0_mip9	PF3D7-1371700_S0_Sub0_mip10	PF3D7-1371700_S0_Sub0_mip11	PF3D7-1371800_S0_Sub0_mip1	PF3D7-1371800_S0_Sub0_mip2	PF3D7-1371800_S0_Sub0_mip3	PF3D7-1371800_S0_Sub0_mip4	PF3D7-1371800_S0_Sub0_mip5	PF3D7-1371800_S0_Sub0_mip6	PF3D7-1371800_S0_Sub0_mip7	PF3D7-1371800_S0_Sub0_mip8	PF3D7-1371900_S0_Sub0_mip0	PF3D7-1371900_S0_Sub0_mip1	PF3D7-1371900_S0_Sub0_mip2	PF3D7-1371900_S0_Sub0_mip3	PF3D7-1371900_S0_Sub0_mip4	PF3D7-1371900_S0_Sub0_mip5	PF3D7-1372000_S0_Sub0_mip1	PF3D7-1372000_S0_Sub0_mip3	PF3D7-1372000_S0_Sub0_mip4	PF3D7-1372000_S0_Sub0_mip5	PF3D7-1372000_S0_Sub0_mip6	PF3D7-1372000_S0_Sub0_mip7	PF3D7-1372000_S0_Sub0_mip8	PF3D7-1372000_S0_Sub0_mip10	PF3D7-1372100_S0_Sub0_mip0
chr13	chr13	chr13	chr13	chr13	chr13	chr13	chr13	chr13	chr13	chr13	chr13	chr13	chr13	chr13	chr13	chr13	chr13	chr13	chr13	chr13	chr13	chr13	chr13	chr13	chr13	chr13	chr13	chr13	chr13	chr13	chr13	chr13	chr13	chr13	chr13	chr13	chr13	chr13	chr13	chr13
2816383	2817675	2817881	2818001	2818179	2818278	2818861	2820938	2821300	2821460	2821662	2821820	2822011	2822205	2822308	2822469	2822584	2823252	2824238	2824387	2824557	2824720	2824862	2825044	2825212	2825286	2829900	2829964	2830165	2830343	2830434	2830641	2832906	2833241	2833330	2833491	2833575	2833764	2833883	2834183	2836945
2816585	2817894	2818091	2818208	2818356	2818498	2819039	2821144	2821493	2821668	2821870	2822011	2822216	2822373	2822491	2822659	2822761	2823451	2824413	2824571	2824756	2824924	2825065	2825213	2825411	2825480	2830069	2830171	2830368	2830520	2830643	2830843	2833066	2833442	2833507	2833682	2833782	2833939	2834055	2834346	2837114
PF3D7-1371600	PF3D7-1371600	PF3D7-1371600	PF3D7-1371600	PF3D7-1371600	PF3D7-1371600	PF3D7-1371600	PF3D7-1371700	PF3D7-1371700	PF3D7-1371700	PF3D7-1371700	PF3D7-1371700	PF3D7-1371700	PF3D7-1371700	PF3D7-1371700	PF3D7-1371700	PF3D7-1371700	PF3D7-1371700	PF3D7-1371800	PF3D7-1371800	PF3D7-1371800	PF3D7-1371800	PF3D7-1371800	PF3D7-1371800	PF3D7-1371800	PF3D7-1371800	PF3D7-1371900	PF3D7-1371900	PF3D7-1371900	PF3D7-1371900	PF3D7-1371900	PF3D7-1371900	PF3D7-1372000	PF3D7-1372000	PF3D7-1372000	PF3D7-1372000	PF3D7-1372000	PF3D7-1372000	PF3D7-1372000	PF3D7-1372000	PF3D7-1372100
0	0	0	0	0	0	0	0	0	0	0	0	0	0	0	0	0	0	0	0	0	0	0	0	0	0	0	0	0	0	0	0	0	0	0	0	0	0	0	0	0
0	0	0	0	0	0	0	0	0	0	0	0	0	0	0	0	0	0	0	0	0	0	0	0	0	0	0	0	0	0	0	0	0	0	0	0	0	0	0	0	0
19	23	17	19	28	35	29	4	6	5	6	5	4	12	27	10	14	6	14	12	11	6	12	11	10	10	5	1	17	31	26	1	12	30	59	44	15	22	20	5	16
0	0	0	0	0	0	0	0	0	0	0	0	0	0	0	0	0	0	0	0	0	0	0	0	0	0	0	0	0	0	0	0	0	0	0	0	0	0	0	0	0
0	0	0	0	0	0	0	0	0	0	0	0	0	0	0	0	0	0	0	0	0	0	0	0	0	0	0	0	0	0	0	0	0	0	0	0	0	0	0	0	0
0	0	0	0	0	0	0	0	0	0	0	0	0	0	0	0	0	0	0	0	0	0	0	0	0	0	0	0	0	0	0	0	0	0	0	0	0	0	0	0	0
0	0	0	0	0	0	0	0	0	0	0	0	0	0	0	0	0	0	0	0	0	0	0	0	0	0	0	0	0	0	0	0	0	0	0	0	0	0	0	0	0
9	2	4	7	14	5	16	0	0	0	0	0	0	1	2	1	2	0	2	0	1	0	0	0	0	0	0	0	1	18	12	0	0	10	44	17	1	6	5	0	4
0	0	0	0	0	0	0	0	0	0	0	0	0	0	0	0	0	0	0	0	0	0	0	0	0	0	0	0	0	0	0	0	0	0	0	0	0	0	0	0	0
0	0	0	0	0	0	0	0	0	0	0	0	0	0	0	0	0	0	0	0	0	0	0	0	0	0	0	0	0	0	0	0	0	0	0	0	0	0	0	0	0
0	0	0	0	0	0	0	0	0	0	0	0	0	0	0	0	0	0	0	0	0	0	0	0	0	0	0	0	0	0	0	0	0	0	0	0	0	0	0	0	0
0	0	0	0	0	0	0	0	0	0	0	0	0	0	0	0	0	0	0	0	0	0	0	0	0	0	0	0	0	0	0	0	0	0	0	0	0	0	0	0	0
0	0	0	0	0	0	1	0	0	0	0	0	0	0	0	0	0	0	0	0	0	0	0	0	0	0	0	0	0	0	0	0	0	0	0	0	0	0	0	0	0
0	0	0	0	0	0	0	0	0	0	0	0	0	0	0	0	0	0	0	0	0	0	0	0	0	0	0	0	0	0	0	0	0	0	0	0	0	0	0	0	0
84	87	83	79	80	76	64	7	25	17	27	14	47	46	80	62	57	28	52	61	38	43	52	30	31	53	8	8	66	60	55	2	36	105	137	119	75	43	53	11	50
0	0	0	0	0	0	0	0	0	0	0	0	0	0	0	0	0	0	0	0	0	0	0	0	0	0	0	0	0	0	0	0	0	0	0	0	0	0	0	0	0
887	634	781	640	807	1123	666	157	267	127	231	98	231	470	773	555	577	278	473	441	343	245	331	295	255	352	38	30	555	747	545	75	491	1124	1480	978	622	471	598	85	491
0	0	0	0	0	0	0	0	0	0	0	0	0	0	0	0	0	0	0	0	0	0	0	0	0	0	0	0	0	0	0	0	0	0	0	0	0	0	0	0	0
0	0	0	0	0	0	0	0	0	0	0	0	0	0	0	0	0	0	0	0	0	0	0	0	0	0	0	0	0	0	0	0	0	0	0	0	0	0	0	0	0
0	0	0	0	0	0	0	0	0	0	0	0	0	0	0	0	0	0	0	0	0	0	0	0	0	0	0	0	0	0	0	0	0	0	0	0	0	0	0	0	0
0	0	0	0	0	0	0	0	0	0	0	0	0	0	0	0	0	0	0	0	0	0	0	0	0	0	0	0	0	0	0	0	0	0	0	0	0	0	0	0	0
[illegible]	[illegible]	[illegible]	[illegible]	[illegible]	[illegible]	[illegible]	[illegible]	[illegible]	[illegible]	[illegible]	[illegible]	[illegible]	[illegible]	[illegible]	[illegible]	[illegible]	[illegible]	[illegible]	[illegible]	[illegible]	[illegible]	[illegible]	[illegible]	[illegible]	[illegible]	[illegible]	[illegible]	[illegible]	[illegible]	[illegible]	[illegible]	[illegible]	[illegible]	[illegible]	[illegible]	[illegible]	[illegible]	[illegible]	[illegible]	[illegible]

PF3D7-1372100_S0_Sub0_mip1	PF3D7-1372100_S0_Sub0_mip2	PF3D7-1372100_S0_Sub0_mip3	PF3D7-1372100_S0_Sub0_mip4	PF3D7-1372100_S0_Sub0_mip5	PF3D7-1372100_S0_Sub0_mip6	PF3D7-1372100_S0_Sub0_mip7	PF3D7-1372100_S0_Sub0_mip8	PF3D7-1372100_S0_Sub0_mip9	PF3D7-1372100_S0_Sub0_mip10	PF3D7-1372100_S0_Sub0_mip11	PF3D7-1372100_S0_Sub0_mip12	PF3D7-1372100_S0_Sub0_mip13	PF3D7-1372100_S0_Sub0_mip14	PF3D7-1372100_S0_Sub0_mip15	hrp3_S0_Sub0_mip0	hrp3_S0_Sub0_mip2	hrp3_S0_Sub0_mip3	hrp3_S0_Sub0_mip4	hrp3_S0_Sub0_mip5	hrp3_S0_Sub0_mip6	PF3D7-1372400_S0_Sub0_mip0	PF3D7-1372400_S0_Sub0_mip1	PF3D7-1372400_S0_Sub0_mip2	PF3D7-1372400_S0_Sub0_mip3	PF3D7-1372400_S0_Sub0_mip4	PF3D7-1372400_S0_Sub0_mip5	PF3D7-0831400_S0_Sub0_mip0	PF3D7-0831400_S0_Sub0_mip1	PF3D7-0831400_S0_Sub0_mip2	PF3D7-0831400_S0_Sub0_mip3	PF3D7-0831400_S0_Sub0_mip4	PF3D7-0831400_S0_Sub0_mip5	PF3D7-0831400_S0_Sub0_mip6	PF3D7-0831400_S0_Sub0_mip7	PF3D7-0831500_S0_Sub0_mip1	PF3D7-0831500_S0_Sub0_mip2	PF3D7-0831500_S0_Sub0_mip3	PF3D7-0831500_S0_Sub0_mip4	PF3D7-0831500_S0_Sub0_mip5	PF3D7-0831500_S0_Sub0_mip6	PF3D7-0831500_S0_Sub0_mip7	PF3D7-0831500_S0_Sub0_mip8	PF3D7-0831500_S0_Sub0_mip9
chr13	chr13	chr13	chr13	chr13	chr13	chr13	chr13	chr13	chr13	chr13	chr13	chr13	chr13	chr13	chr13	chr13	chr13	chr13	chr13	chr13	chr13	chr13	chr13	chr13	chr13	chr13	chr8	chr8	chr8	chr8	chr8	chr8	chr8	chr8	chr8	chr8	chr8	chr8	chr8	chr8	chr8	chr8	chr8
2837011	2837149	2837337	2837460	2837604	2837734	2837874	2838030	2838146	2838293	2838437	2838545	2838704	2838819	2838998	2840643	2840751	2840794	2841009	2841140	2841246	2852540	2852715	2852916	2853114	2853213	2853355	1344451	1344498	1344701	1344779	1344968	1345114	1345238	1345510	1353428	1353580	1353726	1353887	1354006	1354153	1354246	1354391	1354535
2837176	2837347	2837545	2837631	2837816	2837933	2838075	2838197	2838330	2838492	2838650	2838757	2838871	2839006	2839199	2840859	2840954	2841015	2841205	2841341	2841436	2852728	2852915	2853119	2853312	2853379	2853533	1344645	1344701	1344906	1344968	1345115	1345327	1345442	1345689	1353627	1353743	1353924	1354058	1354194	1354316	1354421	1354562	1354733
PF3D7-1372100	PF3D7-1372100	PF3D7-1372100	PF3D7-1372100	PF3D7-1372100	PF3D7-1372100	PF3D7-1372100	PF3D7-1372100	PF3D7-1372100	PF3D7-1372100	PF3D7-1372100	PF3D7-1372100	PF3D7-1372100	PF3D7-1372100	PF3D7-1372100	hrp3	hrp3	hrp3	hrp3	hrp3	hrp3	PF3D7-1372400	PF3D7-1372400	PF3D7-1372400	PF3D7-1372400	PF3D7-1372400	PF3D7-1372400	PF3D7-0831400	PF3D7-0831400	PF3D7-0831400	PF3D7-0831400	PF3D7-0831400	PF3D7-0831400	PF3D7-0831400	PF3D7-0831400	PF3D7-0831500	PF3D7-0831500	PF3D7-0831500	PF3D7-0831500	PF3D7-0831500	PF3D7-0831500	PF3D7-0831500	PF3D7-0831500	PF3D7-0831500
0	0	0	0	0	0	0	0	0	0	0	0	0	0	0	0	0	0	0	0	0	0	0	0	0	0	0	2	9	4	1	5	9	10	0	1	1	1	3	5	0	0	3	1
0	0	0	0	0	0	0	0	0	0	0	0	0	0	0	0	0	0	0	0	0	0	0	0	0	0	0	14	8	10	7	6	17	5	1	14	2	2	6	6	2	9	6	4
15	4	24	44	19	22	18	14	12	2	19	6	18	15	2	11	3	13	2	1	18	37	35	51	51	44	10	20	17	15	26	4	9	4	0	3	8	1	18	16	14	12	20	5
0	0	0	0	0	0	0	0	0	0	0	0	0	0	0	0	0	0	0	0	0	10	10	4	6	5	4	16	6	7	4	7	1	3	0	6	7	2	3	5	7	9	3	5
0	0	0	0	0	0	0	0	0	0	0	0	0	0	0	0	0	0	0	0	0	52	20	47	43	60	2	45	22	33	38	157	8	1	0	3	6	0	30	25	13	15	22	11
0	0	0	0	0	0	0	0	0	0	0	0	0	0	0	0	0	0	0	0	0	0	0	0	0	0	0	15	9	2	43	251	0	2	0	0	0	0	22	8	7	11	8	0
0	0	0	0	0	0	0	0	0	0	0	0	0	0	0	0	0	0	0	0	0	0	0	0	0	0	0	20	15	12	49	116	0	0	0	1	0	1	25	22	22	7	23	4
2	0	29	19	4	1	3	7	0	0	0	2	0	0	0	0	18	17	5	2	28	9	0	3	11	23	0	6	1	1	15	67	0	0	0	1	0	0	4	3	4	3	1	1
0	0	0	0	0	0	0	0	0	0	0	0	0	0	0	0	0	0	0	0	0	0	0	0	0	0	0	101	86	120	106	164	70	50	0	57	34	7	74	59	54	51	97	67
0	0	0	0	0	0	0	0	0	0	0	0	0	0	0	0	0	0	0	0	0	845	673	824	694	652	123	1580	1227	1526	1273	2173	756	294	2	643	452	40	1080	932	647	683	1039	840
0	0	0	0	0	0	0	0	0	0	0	0	0	0	0	0	0	0	0	0	0	0	0	0	0	0	0	327	180	248	240	184	235	275	141	154	139	209	195	183	99	132	192	141
0	0	0	0	0	0	0	0	0	0	0	0	0	0	0	0	0	0	0	0	0	0	0	0	0	0	0	11	10	17	17	8	11	5	1	2	11	2	8	8	2	7	9	5
0	0	0	0	0	0	0	0	0	0	0	0	0	0	0	0	0	0	0	0	0	51	53	44	20	34	43	40	29	51	37	57	39	37	23	15	26	12	29	17	16	19	23	18
0	0	0	0	0	0	0	0	0	0	0	0	0	0	0	0	0	0	0	0	0	9	2	6	5	12	8	9	1	7	8	1	6	6	1	2	7	1	2	2	2	1	3	5
29	15	62	101	65	46	68	53	48	15	61	43	81	60	13	12	36	38	3	6	39	89	96	88	79	70	6	81	71	68	69	3	37	14	0	44	24	2	68	60	32	32	63	52
0	0	0	0	0	0	0	0	0	0	0	0	0	0	0	0	0	0	0	0	0	0	0	0	0	0	0	218	177	211	204	331	116	31	1	64	67	0	134	158	85	79	152	121
391	10	677	838	649	554	664	458	408	116	484	350	608	527	5	196	70	1831	255	153	923	982	680	854	876	848	27	832	583	768	673	1227	291	134	1	247	167	13	543	467	333	314	558	354
0	0	0	0	0	0	0	0	0	0	0	0	0	0	0	0	0	0	0	0	0	0	0	0	0	0	0	92	59	75	68	83	56	44	3	48	40	16	38	50	31	33	49	39
0	0	0	0	0	0	0	0	0	0	0	0	0	0	0	0	0	0	0	0	0	0	0	0	0	0	0	8	5	9	7	19	4	0	0	3	2	0	4	3	8	2	9	1
0	0	0	0	0	0	0	0	0	0	0	0	0	0	0	0	0	0	0	0	0	50	42	32	44	50	1	31	39	21	32	1	8	6	0	12	11	0	16	22	14	18	15	9
0	0	0	0	0	0	0	0	0	0	0	0	0	0	0	0	0	0	0	0	0	168	173	122	128	147	12	153	106	134	97	221	64	32	0	66	49	0	110	70	61	66	78	61

PF3D7-0831600_S0_Sub0_mip0	PF3D7-0831600_S0_Sub0_mip1	PF3D7-0831600_S0_Sub0_mip2	PF3D7-0831600_S0_Sub0_mip3	PF3D7-0831600_S0_Sub0_mip4	PF3D7-0831600_S0_Sub0_mip7	PF3D7-0831600_S0_Sub0_mip8	PF3D7-0831600_S0_Sub0_mip10	PF3D7-0831600_S0_Sub0_mip12	PF3D7-0831600_S0_Sub0_mip14	PF3D7-0831600_S0_Sub0_mip15
chr8	chr8	chr8	chr8	chr8	chr8	chr8	chr8	chr8	chr8	chr8
1358227	1358417	1358512	1358685	1358866	1359316	1359452	1359601	1360022	1360210	1360426
1358416	1358620	1358684	1358896	1359070	1359495	1359625	1359789	1360201	1360415	1360635
PF3D7-0831600	PF3D7-0831600	PF3D7-0831600	PF3D7-0831600	PF3D7-0831600	PF3D7-0831600	PF3D7-0831600	PF3D7-0831600	PF3D7-0831600	PF3D7-0831600	PF3D7-0831600
3	3	5	1	5	3	5	3	6	7	3
9	1	3	6	6	12	1	12	11	4	4
19	16	11	2	8	14	10	52	20	5	3
3	4	9	1	2	4	2	15	8	3	1
23	16	23	3	8	12	6	65	48	2	0
10	0	8	0	1	2	5	82	30	0	0
17	2	10	0	9	9	7	67	27	0	0
0	0	1	0	0	0	1	28	5	0	0
72	48	63	28	31	72	46	112	102	30	33
964	624	889	264	615	948	567	1396	1396	222	85
171	108	131	88	126	167	83	269	242	142	273
5	3	10	2	5	4	4	11	20	6	3
40	37	20	13	12	27	19	38	42	25	49
5	5	7	2	6	4	3	7	4	0	6
48	49	51	15	25	53	26	75	80	12	14
132	44	136	28	73	110	77	206	215	16	31
546	306	477	110	282	508	281	819	649	95	21
49	45	44	28	54	71	49	67	74	32	24
5	7	3	3	3	7	5	19	13	3	0
14	5	20	5	9	20	11	45	33	6	3
83	44	76	21	46	61	54	152	118	24	12

PF3D7-0831600_S0_Sub0_mip17	PF3D7-0831600_S0_Sub0_mip21	PF3D7-0831600_S0_Sub0_mip22	PF3D7-0831600_S0_Sub0_mip23	PF3D7-0831600_S0_Sub0_mip25	PF3D7-0831600_S0_Sub0_mip26	PF3D7-0831600_S0_Sub0_mip27	PF3D7-0831600_S0_Sub0_mip29	PF3D7-0831600_S0_Sub0_mip32	PF3D7-0831600_S0_Sub0_mip33
chr8	chr8	chr8	chr8	chr8	chr8	chr8	chr8	chr8	chr8
1360699	1360950	1361101	1361271	1361516	1361679	1361770	1362282	1362452	1362686
1360912	1361117	1361299	1361441	1361685	1361888	1361964	1362464	1362651	1362852
PF3D7-0831600	PF3D7-0831600	PF3D7-0831600	PF3D7-0831600	PF3D7-0831600	PF3D7-0831600	PF3D7-0831600	PF3D7-0831600	PF3D7-0831600	PF3D7-0831600
0	4	6	5	5	2	5	2	5	0
4	13	5	17	12	11	7	8	13	7
5	27	4	39	17	8	19	23	33	7
2	4	2	5	10	3	4	7	9	1
2	53	0	59	27	14	19	41	33	20
0	33	0	28	9	2	1	64	23	3
0	44	0	33	8	5	5	37	42	13
0	9	0	10	1	0	0	14	5	0
36	159	0	153	95	80	53	100	147	82
386	1621	227	1560	1078	923	829	1229	1449	872
116	328	294	261	189	210	193	172	251	140
5	9	7	12	5	4	5	8	16	6
33	41	19	41	38	34	20	27	49	28
2	11	3	10	9	0	3	8	7	1
38	77	8	108	60	51	47	62	90	50
49	278	2	222	158	135	136	174	200	107
163	821	0	834	496	465	377	633	850	431
34	68	14	87	67	64	53	59	77	49
0	16	0	17	10	8	4	11	8	11
4	46	4	32	32	19	14	39	39	24
40	157	0	141	104	98	69	109	113	55

PF3D7-0831600_S0_Sub0_mip34	PF3D7-0831600_S0_Sub0_mip35	PF3D7-0831600_S0_Sub0_mip36	PF3D7-0831600_S0_Sub0_mip37	PF3D7-0831700_S0_Sub0_mip1	PF3D7-0831700_S0_Sub0_mip2	PF3D7-0831700_S0_Sub0_mip3	PF3D7-0831700_S0_Sub0_mip4	PF3D7-0831700_S0_Sub0_mip5	PF3D7-0831700_S0_Sub0_mip6
chr8	chr8	chr8	chr8	chr8	chr8	chr8	chr8	chr8	chr8
1362870	1362995	1363201	1363389	1365415	1365517	1365668	1365845	1365922	1366111
1363048	1363185	1363415	1363584	1365577	1365690	1365855	1366018	1366112	1366306
PF3D7-0831600	PF3D7-0831600	PF3D7-0831600	PF3D7-0831600	PF3D7-0831700	PF3D7-0831700	PF3D7-0831700	PF3D7-0831700	PF3D7-0831700	PF3D7-0831700
0	5	2	2	4	0	1	5	1	3
6	10	4	7	4	3	9	6	7	4
30	41	16	40	18	37	31	40	23	16
1	10	6	2	3	4	9	11	7	3
24	55	19	38	28	72	49	63	32	42
11	22	7	51	16	54	24	114	53	26
28	33	18	43	23	68	45	106	48	27
12	5	3	8	3	26	7	38	11	12
82	143	59	116	56	155	116	140	86	53
1042	1703	764	1498	838	1740	1356	1781	1072	750
155	302	170	227	132	187	197	179	185	112
9	16	9	2	9	17	10	3	4	5
29	44	7	30	17	46	23	46	25	17
3	6	5	7	3	7	7	3	5	2
61	89	46	84	49	100	64	99	66	26
125	252	100	229	139	193	198	283	141	89
563	887	415	754	456	927	624	999	575	368
56	126	29	78	29	91	68	95	55	25
9	14	2	16	3	9	13	14	9	14
25	38	15	30	27	29	28	40	33	17
61	130	60	114	72	112	123	192	123	67

PF3D7-0831700_S0_Sub0_mip7	PF3D7-0831700_S0_Sub0_mip8	PF3D7-0831700_S0_Sub0_mip9	PF3D7-0831700_S0_Sub0_mip10	PF3D7-0831700_S0_Sub0_mip11	PF3D7-0831700_S0_Sub0_mip12	PF3D7-0831700_S0_Sub0_mip13	PF3D7-0831700_S0_Sub0_mip14	PF3D7-0831700_S0_Sub0_mip15	PF3D7-0831700_S0_Sub0_mip16
chr8	chr8	chr8	chr8	chr8	chr8	chr8	chr8	chr8	chr8
1366184	1366347	1366443	1366597	1366771	1366886	1367010	1367148	1367243	1367406
1366356	1366532	1366638	1366801	1366959	1367107	1367187	1367355	1367422	1367616
PF3D7-0831700	PF3D7-0831700	PF3D7-0831700	PF3D7-0831700	PF3D7-0831700	PF3D7-0831700	PF3D7-0831700	PF3D7-0831700	PF3D7-0831700	PF3D7-0831700
2	3	3	2	7	3	4	1	5	5
0	12	12	5	11	5	4	2	18	10
18	17	38	41	37	25	25	14	66	2
1	3	8	1	3	5	7	0	7	4
23	33	56	8	58	44	57	26	110	5
39	17	32	10	83	20	29	25	169	0
27	27	43	5	52	31	45	17	106	0
18	3	12	1	12	6	9	3	47	0
46	110	117	42	152	98	136	56	222	38
512	1161	1568	500	1518	1033	1486	767	2154	424
56	156	226	221	320	199	262	169	364	216
3	12	11	6	14	7	15	10	18	5
8	32	39	8	30	30	29	18	65	44
3	7	12	2	11	1	8	2	12	4
28	79	92	18	75	53	94	43	119	11
91	176	247	128	254	168	247	126	360	40
263	616	974	308	821	599	845	421	1255	164
22	74	81	35	67	46	90	37	126	51
5	6	16	3	12	7	22	2	24	6
12	38	46	27	41	23	47	26	61	6
58	64	186	83	119	78	162	66	228	36

PF3D7-0831750_S0_Sub0_mip0	PF3D7-0831750_S0_Sub0_mip1	PF3D7-0831750_S0_Sub0_mip2	PF3D7-0831750_S0_Sub0_mip3	PF3D7-0831750_S0_Sub0_mip4	PF3D7-0831750_S0_Sub0_mip5	PF3D7-0831750_S0_Sub0_mip6	hrp2_S0_Sub0_mip0	hrp2_S0_Sub0_mip1	hrp2_S0_Sub0_mip3	hrp2_S0_Sub0_mip4	hrp2_S0_Sub0_mip5	hrp2_S0_Sub0_mip6	hrp2_S0_Sub0_mip7	hrp2_S0_Sub0_mip8	PF3D7-0831900_S0_Sub0_mip0	PF3D7-0832000_S0_Sub0_mip0	PF3D7-0832000_S0_Sub0_mip1	PF3D7-0832000_S0_Sub0_mip2	PF3D7-0832000_S0_Sub0_mip3	PF3D7-0832000_S0_Sub0_mip4	PF3D7-0832000_S0_Sub0_mip5	PF3D7-0832100_S0_Sub0_mip0	PF3D7-0832100_S0_Sub0_mip1	PF3D7-0832100_S0_Sub0_mip2	PF3D7-0832100_S0_Sub0_mip3	PF3D7-0832100_S0_Sub0_mip4	PF3D7-0832100_S0_Sub0_mip5	PF3D7-0832100_S0_Sub0_mip6	PF3D7-0832100_S0_Sub0_mip7	PF3D7-0832200-2_S0_Sub0_mip0	PF3D7-0832200-2_S0_Sub0_mip1	PF3D7-0832200-2_S0_Sub0_mip2	PF3D7-0832200-2_S0_Sub0_mip3	PF3D7-0832200-2_S0_Sub0_mip4	PF3D7-0832300_S0_Sub0_mip0
chr8	chr8	chr8	chr8	chr8	chr8	chr8	chr8	chr8	chr8	chr8	chr8	chr8	chr8	chr8	chr8	chr8	chr8	chr8	chr8	chr8	chr8	chr8	chr8	chr8	chr8	chr8	chr8	chr8	chr8	chr8	chr8	chr8	chr8	chr8	chr8
1371664	1371800	1371969	1372168	1372345	1372430	1372614	1374269	1374297	1374430	1374468	1374619	1374630	1374763	1374871	1380756	1383785	1383870	1384048	1384194	1384364	1384390	1386570	1386610	1386788	1386959	1387025	1387189	1387281	1387448	1392258	1392429	1392665	1392878	1392909	1397685
1371848	1371998	1372175	1372367	1372509	1372615	1372807	1374450	1374521	1374625	1374661	1374805	1374822	1374985	1375047	1380927	1383949	1384053	1384225	1384363	1384560	1384585	1386754	1386808	1386973	1387131	1387224	1387362	1387480	1387625	1392431	1392612	1392854	1393071	1393108	1397861
PF3D7-0831750	PF3D7-0831750	PF3D7-0831750	PF3D7-0831750	PF3D7-0831750	PF3D7-0831750	PF3D7-0831750	hrp2	hrp2	hrp2	hrp2	hrp2	hrp2	hrp2	hrp2	PF3D7-0831900	PF3D7-0832000	PF3D7-0832000	PF3D7-0832000	PF3D7-0832000	PF3D7-0832000	PF3D7-0832000	PF3D7-0832100	PF3D7-0832100	PF3D7-0832100	PF3D7-0832100	PF3D7-0832100	PF3D7-0832100	PF3D7-0832100	PF3D7-0832100	PF3D7-0832200-2	PF3D7-0832200-2	PF3D7-0832200-2	PF3D7-0832200-2	PF3D7-0832200-2	PF3D7-0832300
0	1	2	6	1	2	1	0	0	0	0	0	0	0	0	0	0	0	0	0	0	0	0	0	0	0	0	0	0	0	0	0	0	0	0	0
3	9	3	9	4	10	0	0	0	0	0	0	0	0	0	0	0	0	0	0	0	0	0	0	0	0	0	0	0	0	0	0	0	0	0	0
1	6	3	6	19	14	0	2	0	0	0	0	0	1	0	2	0	0	0	0	0	0	2	0	1	2	0	0	0	1	0	1	0	0	1	12
1	5	3	5	5	4	0	0	0	0	0	0	0	0	0	0	0	0	0	0	0	0	0	0	0	0	0	0	0	0	0	0	0	0	0	0
0	13	9	15	22	14	0	5	0	0	8	2	3	0	54	0	13	24	3	0	1	0	19	26	56	36	58	25	17	11	0	11	46	9	35	20
0	0	1	3	6	2	0	0	0	0	0	0	0	0	0	0	0	0	0	0	0	0	0	0	0	0	0	0	0	0	0	0	0	0	0	0
0	4	3	5	11	8	0	0	0	0	0	0	0	0	0	0	0	0	0	0	0	0	0	0	0	0	0	0	0	0	0	0	0	0	0	0
0	0	1	0	0	0	0	0	0	0	0	0	0	0	0	0	0	0	0	0	0	0	0	0	0	0	0	0	0	0	0	0	0	0	0	0
4	68	36	67	64	62	4	15	1	1	8	3	5	1	57	25	0	0	0	0	0	0	0	0	0	0	0	0	0	0	0	0	0	0	0	0
34	761	472	945	893	708	0	0	0	0	0	0	0	0	1	0	0	0	0	0	0	0	0	0	0	0	0	0	2	0	0	0	0	0	1	0
97	113	123	177	152	164	167	0	0	0	0	0	0	0	0	0	0	0	0	0	0	0	0	0	0	0	0	0	0	0	0	0	0	0	0	0
1	11	3	8	7	7	0	0	0	0	0	0	0	0	0	0	0	0	0	0	0	0	0	0	0	0	0	0	0	0	0	0	0	0	0	0
15	16	19	42	29	23	15	3	0	0	2	1	0	0	10	19	0	0	0	0	0	0	0	0	0	0	0	0	0	0	0	0	0	0	0	0
3	4	2	5	3	1	1	2	0	0	0	0	1	0	1	1	0	0	0	0	0	0	0	0	0	0	0	0	0	0	0	0	0	0	0	6
2	37	20	63	36	33	0	6	1	2	5	2	1	0	38	11	0	0	0	0	0	0	0	0	0	0	0	0	0	0	0	0	0	0	0	32
0	75	46	109	119	120	2	64	12	1	34	12	28	13	247	17	0	0	0	0	0	0	0	0	0	0	0	0	0	0	0	0	0	0	0	0
13	308	161	378	481	317	0	63	22	40	52	25	39	6	633	72	292	563	154	34	87	151	430	451	641	580	1053	377	486	362	15	350	669	305	670	334
4	35	32	47	57	42	0	2	0	1	2	0	6	0	31	0	69	66	28	25	27	37	49	81	63	63	93	41	52	43	6	38	58	26	91	35
0	3	1	6	8	3	0	4	1	0	2	0	0	1	9	2	0	0	0	0	0	0	0	0	0	0	0	0	0	0	0	0	0	0	0	0
0	10	9	23	34	18	0	5	0	1	6	1	9	1	82	6	15	24	7	11	1	9	12	21	38	25	51	19	19	26	0	0	0	0	0	0
0	53	45	77	89	74	0	16	2	1	6	2	5	4	90	15	70	109	24	28	10	38	106	106	100	92	180	63	81	74	2	83	99	81	117	61
0	9	10	9	12	3	0	2	0	0	0	0	0	0	9	1	8	12	1	3	2	9	7	17	13	6	4	8	8	9	0	8	14	6	12	4
2	19	12	26	31	26	13	2	0	1	1	1	2	0	16	10	27	36	5	12	3	12	22	18	22	29	39	33	26	14	0	0	0	0	0	0
0	3	2	3	1	3	0	0	0	0	0	0	0	0	0	0	0	0	0	0	0	0	1	0	0	2	0	1	0	1	0	0	1	1	0	0
1	6	4	13	8	11	11	0	0	0	3	0	0	0	10	5	10	9	3	6	3	5	5	8	12	11	13	8	11	12	0	0	0	0	0	0
2	14	5	9	15	9	0	0	0	0	0	0	0	0	0	0	0	0	0	0	0	0	0	0	0	0	0	0	0	0	0	0	0	0	0	0
52	113	85	170	118	128	19	0	0	0	0	0	0	0	0	0	0	0	0	0	0	0	0	0	0	0	0	0	0	0	0	0	0	0	0	0
14	28	21	25	19	37	9	0	0	0	0	0	0	0	0	0	0	0	0	0	0	0	0	0	0	0	0	0	0	0	0	0	0	0	0	0
0	11	4	14	15	12	0	0	0	0	0	0	0	0	0	0	0	0	0	0	0	0	0	0	0	0	0	0	0	0	0	0	0	0	0	0
1	3	0	4	6	2	1	1	0	0	0	0	0	0	1	0	7	9	1	0	0	1	4	3	5	2	6	4	4	3	0	1	9	2	10	3
1	0	9	4	7	1	0	0	0	0	0	0	0	0	0	0	0	0	0	0	0	0	0	0	0	0	0	0	0	0	0	0	0	0	0	0
2	3	14	6	6	11	8	0	0	0	0	0	0	0	0	0	0	0	0	0	0	0	0	0	0	0	0	0	0	0	0	0	0	0	0	0
12	11	9	13	21	15	6	0	0	0	0	0	0	0	0	0	0	0	0	0	0	0	0	0	0	0	0	0	0	0	0	0	0	0	0	0
21	55	40	35	59	43	1	0	0	0	0	0	0	0	0	0	0	0	0	0	0	0	0	0	0	0	0	0	0	0	0	0	0	0	0	0
0	6	6	13	17	16	0	0	0	0	0	0	0	0	0	0	0	0	0	0	0	0	0	0	0	0	0	0	0	0	0	0	0	0	0	0
0	3	3	4	4	2	1	0	0	0	0	0	0	0	0	0	0	0	0	0	0	0	0	0	0	0	0	0	0	0	0	0	0	0	0	0
1	10	10	12	7	6	0	0	0	0	0	0	0	0	0	0	0	0	0	0	0	0	0	0	0	0	0	0	0	0	0	0	0	0	0	0
1	2	3	4	8	5	4	0	0	0	0	0	0	0	0	0	0	0	0	0	0	0	0	0	0	0	0	0	0	0	0	0	0	0	0	0
34	45	38	60	60	37	61	27	7	1	29	7	11	5	98	0	0	0	0	0	0	0	0	0	0	0	0	0	0	0	0	0	0	0	0	0
9	7	6	13	13	13	13	0	0	0	0	0	0	0	0	0	0	0	0	0	0	0	0	0	0	0	0	0	0	0	0	0	0	0	0	0
13	10	20	26	10	19	0	0	0	0	0	0	0	0	0	0	0	0	0	0	0	0	0	0	1	0	0	0	0	0	0	0	0	1	0	0
11	35	19	29	18	24	0	0	0	0	0	0	0	0	0	0	0	0	0	0	0	0	0	0	0	0	0	0	0	0	0	0	0	0	0	0

Supplementary Table 7B. Unique molecular identifier (UMI) counts by sample and locus. **Normalized coverage.**

MIP	PF3D7-1370500_S0_Sub0_mip2	PF3D7-1370500_S0_Sub0_mip3	PF3D7-1370500_S0_Sub0_mip4	PF3D7-1370500_S0_Sub0_mip5	PF3D7-1370500_S0_Sub0_mip6	PF3D7-1370900_S0_Sub0_mip0	PF3D7-1370900_S0_Sub0_mip1	PF3D7-1371000_S0_Sub0_mip0	PF3D7-1371000_S0_Sub0_mip1	PF3D7-1371000_S0_Sub0_mip3	PF3D7-1371000_S0_Sub0_mip2	PF3D7-1371000_S0_Sub0_mip4	PF3D7-1371000_S0_Sub0_mip5
**Chrom**	chr11	chr11	chr11	chr11	chr11	chr11	chr11	chr11	chr11	chr11	chr11	chr11	chr11
**begin**	1920280	1920386	1920588	1920716	1920809	1923308	1923404	1928534	1928670	1928875	1928878	1929064	1929217
**end**	1920462	1920590	1920760	1920884	1921024	1923492	1923648	1928747	1928856	1929058	1929088	1929266	1929412
**Gene**	PF3D7-1370500	PF3D7-1370500	PF3D7-1370500	PF3D7-1370500	PF3D7-1370500	PF3D7-1370900	PF3D7-1370900	PF3D7-1371000	PF3D7-1371000	PF3D7-1371000	PF3D7-1371000	PF3D7-1371000	PF3D7-1371000
**Sample ID**													
**6**	0	1.354799391	0	0	0	1.333333333	0	0.356877323	1.224742268	0	0	0.231909055	1.229071332
**52**	0	1.642181079	0	0	0	1.287878788	1.102771851	1.216627239	1.484536082	0	0.318181818	1.686611311	0.558668788
**141**	0	0.233586102	0.965517241	1.310344828	0.684368308	0.804597701	0.418292771	0.799897449	0.809456097	0	0	0.759702078	0.776999118
**185**	0.714285714	0	0	0	0	1.785714286	0.866463597	1.784386617	1.312223859	0	0	0.828246626	0.877908095
**228**	0.306122449	0	0	0	0	0.74829932	0.495122055	0.619072908	0.249947402	0	0	0.68626149	0.731590079
**311**	0	0.07526663	0	0	0	0.111111111	0.606524518	0.624535316	0.034020619	0	0	0.077303018	0.170704352
**342**	0	0.107523761	0	0	0	0.198412698	0.722052998	0.531067446	0.170103093	0	0	0.165649325	0.487726719
**386**	0	0	0	0	0	0	0	0.09913259	0	0	0	0.128838364	0.455211605
**419**	1.102941176	0	0	0	0	1.170343137	0.847350429	1.193964575	1.073150394	0	0	1.125441004	0.896197847
**424**	0.006329114	0.988946391	0	0	0	1.226265823	1.09404739	1.168321491	0.922432468	0	0.00443038	1.090559671	1.047562908
**447**	0	2.878517789	0	0	0	1.876590331	1.388987446	1.402991004	1.897103958	0	0	0.964812482	1.10241131
**451**	0.333333333	1.053732859	0	0	0	1.388888889	1.213049036	0.594795539	0.884536083	0	0	0.927636221	0.409690444
**476**	0	1.247841544	0	0	0	1.425438596	0.478835146	0.023478771	1.181768855	0	0	1.708803565	1.401572572
**485**	0.625	0	1.5	1	1.313383298	0.9375	0	1.338289963	0.765463918	0	2.625	0.869658957	1.02422611
**488**	0.730337079	1.014831004	0	0	0	1.43258427	0.6133394	1.182907982	0.89447469	0.015474926	0	0.820801713	1.10478322
**500**	1.133603239	0.996444896	0	0	0	1.224696356	1.006781588	1.13059314	0.847072082	0	0.028340081	0.81684566	1.028372775
**505**	0.99408284	0	0	0	0	1.149901381	0.969003668	0.657794593	0.736777893	0.92578514	1.217751479	1.02094874	1.059377066
**517**	2.098765432	0	1.234567901	1.259259259	0	1.450617284	0.973434411	1.409885722	1.39862543	0	0	1.646267985	1.049515644
**522**	0.769230769	0.17369223	0	0	0	0.833333333	0	0.823563054	0.863600317	0	0	0.445978952	1.496945854
**530**	0	0.534789233	1.210526316	0.842105263	0	1.184210526	0.638446861	1.29133242	1.208627238	0	0	0.610286988	0.889459517
**535**	0.032894737	0	1.065789474	0.921052632	1.1290488	1.354166667	1.236990793	0.880453923	1.013903961	0	0	0.74760156	1.03770277

PF3D7-1370500_S0_Sub0_mip4	PF3D7-1370500_S0_Sub0_mip5	PF3D7-1370500_S0_Sub0_mip6	PF3D7-1370900_S0_Sub0_mip0	PF3D7-1370900_S0_Sub0_mip1	PF3D7-1370900_S0_Sub0_mip3	PF3D7-1370900_S0_Sub0_mip5	PF3D7-1371000_S0_Sub0_mip0	PF3D7-1371000_S0_Sub0_mip1	PF3D7-1371000_S0_Sub0_mip3	PF3D7-1371000_S0_Sub0_mip2	PF3D7-1371000_S0_Sub0_mip4	PF3D7-1371000_S0_Sub0_mip7	PF3D7-1371000_S0_Sub0_mip9
chr13	chr13	chr13	chr13	chr13	chr13	chr13	chr13	chr13	chr13	chr13	chr13	chr13	chr13
2794590	2794718	2794812	2797288	2797384	2797866	2798027	2802545	2802681	2802887	2802890	2803077	2803604	2803985
2794763	2794887	2795027	2797472	2797578	2798036	2798194	2802759	2802868	2803071	2803101	2803279	2803824	2804201
PF3D7-1370500	PF3D7-1370500	PF3D7-1370500	PF3D7-1370900	PF3D7-1370900	PF3D7-1370900	PF3D7-1370900	PF3D7-1371000	PF3D7-1371000	PF3D7-1371000	PF3D7-1371000	PF3D7-1371000	PF3D7-1371000	PF3D7-1371000
0.73	0.933333333	0.914285714	0	2.378440816	2	3.05	0	0	1.163814181	1.466666667	0	0.613474076	1.374562798
2.101515152	2.015151515	1.402597403	0	1.351386827	1.727272727	2.772727273	0	0	1.738164036	0.933333333	0	1.673111117	1.374562798
0.964942529	0.965517241	0.84729064	0	0.717633005	1	0.262931035	0.040706013	0	0.544642104	0.556321839	0	3.226027471	0.687281399
1.216666667	2	1.959183673	0	1.698886297	0.714285714	0	0	0	0.712539294	1.466666667	0	1.752783075	0.785464456
1.067687075	1.166666667	0.827988338	0	0.364047064	0.87755102	0	0.457734968	0	0.475026196	0.478911565	0	1.752783075	1.065987476
0.73	1.361111111	0.095238095	0.083333333	0.099101701	1.5	0	0	0	0.055419723	0	2.582365591	0.204491359	1.695294118
0.782142857	0.972222222	0.571428571	0	0.141573858	1.095238095	0	0	0	0.178134824	0.034920635	0	0.255614199	0.883647513
0.675925926	0.777777778	0.571428571	0	0	1.666666667	0	0	0	0	0	0	0	1.450927398
1.127205882	1.303921569	1.147058824	0.018382353	0.612098739	0.779411765	0.784926471	0.008679959	0	1.118581907	0.959803922	0.569639469	0.067662582	1.041029178
1.374525316	1.363818565	1.436889693	0.014240506	0.999170944	0.935443038	0.289556962	0	0	1.083841416	0.972827004	0	1.465737113	0.987423276
1.481361323	1.166666667	1.570338059	0	1.724823492	1.13740458	3.201335878	0	0	1.24377088	1.399491094	0	0	1.004689984
0.811111111	1.322222222	1.485714286	0	1.387423809	1.2	0	0	0	1.274653627	1.368888889	0	1.840422229	0.733100159
1.312719298	1.043859649	1.77443609	0	2.581860096	1.052631579	7.223684211	1.677516238	0.713688984	1.531334449	1.58245614	0	5.166097485	0.813885867
0.152083333	0.145833333	0.571428571	2.1875	1.48652551	1.125	0.953125	0	0	0.935207824	0	0	3.834212977	0.429550874
1.025280899	1.232209738	1.290529695	0	0.768316556	1.02247191	0.171348315	0.013263757	0	0.99008269	1.071161049	0.870460312	1.275198923	0.888060235
1.423549258	1.133603239	1.411220359	0	0.890711642	0.793522267	0.092611336	0	0	0.9288738	0.884750337	0	1.32257063	1.160309083
1.271380671	1.26469428	1.345054945	0	0.735346347	0.90887574	0.018047337	0.610493856	0.00279086	0.328583209	0.35408284	0	1.25054331	1.110223798
0.736008231	0.446502058	1.467372134	0.030864198	1.578286344	1	0.564814815	0	0	1.364967249	1.23127572	0.9564317	1.741963427	0.941830065
0.935897436	1.166666667	1.318681319	0	0.457392465	1.538461538	0	0	0	0.575512507	0.564102564	0	0.943806271	0.845884799
0.544298246	0.49122807	1.007518797	0	0.860620032	1.078947368	0.200657895	0	0	0.525028954	0.61754386	0	0.766842596	1.211785625
0.488267544	0.690789474	0.646616541	0.016447368	1.310489594	1.019736842	0.300986842	0.007766279	0	0.902393514	0.781578947	0	1.049363552	0.908839218
[illegible]	[illegible]	[illegible]	[illegible]	[illegible]	[illegible]	[illegible]	[illegible]	[illegible]	[illegible]	[illegible]	[illegible]	[illegible]	[illegible]

PF3D7-1371000_S0_Sub0_mip10	PF3D7-1371000_S0_Sub0_mip13	PF3D7-1371000_S0_Sub0_mip16	PF3D7-1371000_S0_Sub0_mip17	PF3D7-1371000_S0_Sub0_mip18	PF3D7-1371000_S0_Sub0_mip19	PF3D7-1371000_S0_Sub0_mip20	PF3D7-1371000_S0_Sub0_mip22	PF3D7-1371500_S0_Sub0_mip0	PF3D7-1371500_S0_Sub0_mip1	PF3D7-1371500_S0_Sub0_mip2	PF3D7-1371500_S0_Sub0_mip3	PF3D7-1371500_S0_Sub0_mip4	PF3D7-1371600_S0_Sub0_mip0
chr13	chr13	chr13	chr13	chr13	chr13	chr13	chr13	chr13	chr13	chr13	chr13	chr13	chr13
2804128	2804733	2805291	2805378	2805781	2806111	2806117	2806917	2808585	2808671	2808809	2808964	2809086	2811767
2804329	2804952	2805504	2805597	2805997	2806300	2806319	2807127	2808762	2808855	2808983	2809134	2809265	2811979
PF3D7-1371000	PF3D7-1371000	PF3D7-1371000	PF3D7-1371000	PF3D7-1371000	PF3D7-1371000	PF3D7-1371000	PF3D7-1371000	PF3D7-1371500	PF3D7-1371500	PF3D7-1371500	PF3D7-1371500	PF3D7-1371500	PF3D7-1371600
0.798876742	1.775248933	0	0	0.33	0.7	0.731881188	0	0	0	0	0	0	0
1.089377375	0.645545067	0	5.135961384	0.55	0.636363636	0.998019802	0	0	0	0	0	0	0
1.377373692	0.703978025	0	0	1.403448276	1.066091954	1.093615568	0	0.159050556	0.571856929	0.630326504	0.358974359	0.689655172	0.997840173
1.296877827	0.760820971	0	0	1.1	1.25	0.871287129	0	0	0	0	0	0	0
1.474735358	0.905739252	2.388199764	0	1.997959184	0.833333333	0.821499293	0	0	0	0	0	0	0
3.062360843	0.887624467	0.103559105	0	3.061666667	1.069444444	0.528580858	0	0	0	0	0	0	0
1.781045549	0.71855314	0	0	1.492857143	1.027777778	1.01650165	0	0	0	0	0	0	0
2.985700953	1.479374111	0	0	3.911111111	0.583333333	0.677667767	0	0	0	0.225672452	0	0	0.153107751
0.995925884	0.93331102	2.962094983	0.415408641	1.023161765	1.025122549	0.986604543	0.402272727	0	0	0	0	0	0
0.951481505	1.27188721	0	0	0.93778481	0.895305907	0.972753478	0	0	0	0	0	0	0
0.982657199	1.829455007	0	0	0.858587786	1.001908397	1.080129998	0	0	0	0	0	0	0
0.847293514	0.946799431	0.069039403	0	0.586666667	0.777777778	0.650561056	0	0	0	0	0	0	0
1.051153607	1.3314367	3.515559084	0	0.998684211	0.905701754	0.898801459	1.439712919	0	0	0	0	0	0
0.635470135	1.3314367	0	0	0.9625	0.802083333	0.914851485	0	0	0	0	0	0	0
0.946575097	0.857704541	0	0	1.161797753	1.055243446	0.931983535	0.614708887	1.347462014	0.962732919	0.753086722	1.473811582	1.516853933	0.959933992
0.965885203	1.613535974	1.484207413	0.686181076	0.932995951	0.980094467	1.10621718	0	0	0	0	0	0	0
0.922640325	1.253178685	0	0	0.986094675	0.93816568	1.004712637	0	0.736902872	0.644382374	1.011920616	1.09400091	0.603550296	1.081176275
0.999716686	1.030082714	3.388044787	0	1.066049383	1.12345679	0.918616306	0	0	0	0	0	0	0
0.670386077	0.751066856	2.549147195	0	1.057692308	1.256410256	1.595125666	0	0	0	0	0	0	0
1.184936794	0.934341544	3.052268352	0	0.955263158	1.135964912	1.027201668	0	0	0	0	0	0	0
0.879146653	0.875945197	0.027252396	0	0.933552632	0.878837719	0.818551329	0.719856459	0	0	0	0	0	0
[illegible]	[illegible]	[illegible]	[illegible]	[illegible]	[illegible]	[illegible]	[illegible]	[illegible]	[illegible]	[illegible]	[illegible]	[illegible]	[illegible]

PF3D7-1371600_S0_Sub0_mip2	PF3D7-1371600_S0_Sub0_mip3	PF3D7-1371600_S0_Sub0_mip4	PF3D7-1371600_S0_Sub0_mip5	PF3D7-1371600_S0_Sub0_mip6	PF3D7-1371600_S0_Sub0_mip7	PF3D7-1371600_S0_Sub0_mip9	PF3D7-1371600_S0_Sub0_mip10	PF3D7-1371600_S0_Sub0_mip20	PF3D7-1371600_S0_Sub0_mip22	PF3D7-1371600_S0_Sub0_mip24	PF3D7-1371600_S0_Sub0_mip28	PF3D7-1371600_S0_Sub0_mip30
chr13	chr13	chr13	chr13	chr13	chr13	chr13	chr13	chr13	chr13	chr13	chr13	chr13
2811977	2812266	2812420	2812590	2812837	2813009	2813244	2813421	2814557	2814849	2815114	2815719	2816090
2812173	2812441	2812618	2812799	2813043	2813224	2813443	2813614	2814756	2815025	2815330	2815912	2816295
PF3D7-1371600	PF3D7-1371600	PF3D7-1371600	PF3D7-1371600	PF3D7-1371600	PF3D7-1371600	PF3D7-1371600	PF3D7-1371600	PF3D7-1371600	PF3D7-1371600	PF3D7-1371600	PF3D7-1371600	PF3D7-1371600
0	0	0	0	0	0	0	0	0	0	0	0	0
0	0	0	0	0	0	0	0	0	0	0	0	0
0.954445578	0.75862069	1.120689655	0.84538376	0.748691802	0.742819965	1.053379418	1.219682999	0.973831829	1.126436782	0.91954023	1.384627794	1.064039409
0	0	0	0	0	0	0	0	0	0	0	0	0
0	0	0	0	0	0	0	0	0	0	0	0	0
0	0	0	0	0	0	0	0	0	0	0	0	0
0	0	0	0	0	0	0	0	0	0	0	0	0
0	0.916666667	0.866666667	0	0.574393181	0	1.174923197	1.725405218	1.255161024	0.518518519	1.777777778	1.338473534	1.777777778
0	0	0	0	0	0	0	0	0	0	0	0	0
0	0	0	0	0	0	0	0	0	0	0	0	0
0	0	0	0	0	0	0	0	0	0	0	0	0
0	0	0	0	0	0	0	0	0	0	0	0	0
0	0	0	0	0	0	0	0	0	0	0	0	0
0	0	0	0	0	0	0	0	0	0	0	0	0
1.166246704	1.462546816	1.080898876	0.991663646	1.138460193	1.492595172	1.069312123	0.823929458	0.88848477	0.996254682	0.988764045	0.706834338	0.731942215
0	0	0	0	0	0	0	0	0	0	0	0	0
0.990872643	1.09566075	1.067692308	0.896506967	1.112215531	1.159941945	0.999727549	0.996447037	1.036064869	1.052071006	0.915187377	0.974155294	1.010312764
0	0	0	0	0	0	0	0	0	0	0	0	0
0	0	0	0	0	0	0	0	0	0	0	0	0
0	0	0	0	0	0	0	0	0	0	0	0	0
0	0	0	0	0	0	0	0	0	0	0	0	0
[illegible]	[illegible]	[illegible]	[illegible]	[illegible]	[illegible]	[illegible]	[illegible]	[illegible]	[illegible]	[illegible]	[illegible]	[illegible]

PF3D7-1371600_S0_Sub0_mip33	PF3D7-1371600_S0_Sub0_mip42	PF3D7-1371600_S0_Sub0_mip43	PF3D7-1371600_S0_Sub0_mip44	PF3D7-1371600_S0_Sub0_mip45	PF3D7-1371600_S0_Sub0_mip46	PF3D7-1371600_S0_Sub0_mip50	PF3D7-1371700_S0_Sub0_mip0	PF3D7-1371700_S0_Sub0_mip2	PF3D7-1371700_S0_Sub0_mip3	PF3D7-1371700_S0_Sub0_mip4	PF3D7-1371700_S0_Sub0_mip5	PF3D7-1371700_S0_Sub0_mip6	PF3D7-1371700_S0_Sub0_mip7	PF3D7-1371700_S0_Sub0_mip8	PF3D7-1371700_S0_Sub0_mip9	PF3D7-1371700_S0_Sub0_mip10	PF3D7-1371700_S0_Sub0_mip11	PF3D7-1371800_S0_Sub0_mip1	PF3D7-1371800_S0_Sub0_mip2	PF3D7-1371800_S0_Sub0_mip3	PF3D7-1371800_S0_Sub0_mip4	PF3D7-1371800_S0_Sub0_mip5	PF3D7-1371800_S0_Sub0_mip6	PF3D7-1371800_S0_Sub0_mip7	PF3D7-1371800_S0_Sub0_mip8	PF3D7-1371900_S0_Sub0_mip0	PF3D7-1371900_S0_Sub0_mip1	PF3D7-1371900_S0_Sub0_mip2	PF3D7-1371900_S0_Sub0_mip3	PF3D7-1371900_S0_Sub0_mip4	PF3D7-1371900_S0_Sub0_mip5	PF3D7-1372000_S0_Sub0_mip1	PF3D7-1372000_S0_Sub0_mip3	PF3D7-1372000_S0_Sub0_mip4	PF3D7-1372000_S0_Sub0_mip5	PF3D7-1372000_S0_Sub0_mip6	PF3D7-1372000_S0_Sub0_mip7	PF3D7-1372000_S0_Sub0_mip8	PF3D7-1372000_S0_Sub0_mip10	PF3D7-1372100_S0_Sub0_mip0
chr13	chr13	chr13	chr13	chr13	chr13	chr13	chr13	chr13	chr13	chr13	chr13	chr13	chr13	chr13	chr13	chr13	chr13	chr13	chr13	chr13	chr13	chr13	chr13	chr13	chr13	chr13	chr13	chr13	chr13	chr13	chr13	chr13	chr13	chr13	chr13	chr13	chr13	chr13	chr13	chr13
2816383	2817675	2817881	2818001	2818179	2818278	2818861	2820938	2821300	2821460	2821662	2821820	2822011	2822205	2822308	2822469	2822584	2823252	2824238	2824387	2824557	2824720	2824862	2825044	2825212	2825286	2829900	2829964	2830165	2830343	2830434	2830641	2832906	2833241	2833330	2833491	2833575	2833764	2833883	2834183	2836945
2816585	2817894	2818091	2818208	2818356	2818498	2819039	2821144	2821493	2821668	2821870	2822011	2822216	2822373	2822491	2822659	2822761	2823451	2824413	2824571	2824756	2824924	2825065	2825213	2825411	2825480	2830069	2830171	2830368	2830520	2830643	2830843	2833066	2833442	2833507	2833682	2833782	2833939	2834055	2834346	2837114
PF3D7-1371600	PF3D7-1371600	PF3D7-1371600	PF3D7-1371600	PF3D7-1371600	PF3D7-1371600	PF3D7-1371600	PF3D7-1371700	PF3D7-1371700	PF3D7-1371700	PF3D7-1371700	PF3D7-1371700	PF3D7-1371700	PF3D7-1371700	PF3D7-1371700	PF3D7-1371700	PF3D7-1371700	PF3D7-1371700	PF3D7-1371800	PF3D7-1371800	PF3D7-1371800	PF3D7-1371800	PF3D7-1371800	PF3D7-1371800	PF3D7-1371800	PF3D7-1371800	PF3D7-1371900	PF3D7-1371900	PF3D7-1371900	PF3D7-1371900	PF3D7-1371900	PF3D7-1371900	PF3D7-1372000	PF3D7-1372000	PF3D7-1372000	PF3D7-1372000	PF3D7-1372000	PF3D7-1372000	PF3D7-1372000	PF3D7-1372000	PF3D7-1372100
0	0	0	0	0	0	0	0	0	0	0	0	0	0	0	0	0	0	0	0	0	0	0	0	0	0	0	0	0	0	0	0	0	0	0	0	0	0	0	0	0
0	0	0	0	0	0	0	0	0	0	0	0	0	0	0	0	0	0	0	0	0	0	0	0	0	0	0	0	0	0	0	0	0	0	0	0	0	0	0	0	0
0.724137931	1.05705428	0.68	0.865397783	1.030878073	1.133679735	1.333333333	0.551724138	0.560212202	0.98245614	0.607794771	0.862068966	0.30839168	0.827586207	1.104838674	0.517241379	0.735314444	0.574091333	0.934768285	0.896551724	1.043103448	0.488098524	1.056360038	1.086499123	0.961376481	0.753498385	0.654053907	0.10031348	0.921182266	1.258409646	1.476673428	0.184337738	0.90171356	0.778325123	1.284936479	1.197822142	0.711442786	1.517241379	0.896576595	0.948275862	1.097432719
0	0	0	0	0	0	0	0	0	0	0	0	0	0	0	0	0	0	0	0	0	0	0	0	0	0	0	0	0	0	0	0	0	0	0	0	0	0	0	0	0
0	0	0	0	0	0	0	0	0	0	0	0	0	0	0	0	0	0	0	0	0	0	0	0	0	0	0	0	0	0	0	0	0	0	0	0	0	0	0	0	0
0	0	0	0	0	0	0	0	0	0	0	0	0	0	0	0	0	0	0	0	0	0	0	0	0	0	0	0	0	0	0	0	0	0	0	0	0	0	0	0	0
0	0	0	0	0	0	0	0	0	0	0	0	0	0	0	0	0	0	0	0	0	0	0	0	0	0	0	0	0	0	0	0	0	0	0	0	0	0	0	0	0
1.105263158	0.29617946	0.515555556	1.027343567	1.660859118	0.521852577	2.37037037	0	0	0	0	0	0	0.222222222	0.26370635	0.166666667	0.338478077	0	0.430290163	0	0.305555556	0	0	0	0	0	0	0	0.174603175	2.354443853	2.196078431	0	0	0.835978836	3.087719298	1.49122807	0.15282845	1.333333333	0.722242257	0	0.884043023
0	0	0	0	0	0	0	0	0	0	0	0	0	0	0	0	0	0	0	0	0	0	0	0	0	0	0	0	0	0	0	0	0	0	0	0	0	0	0	0	0
0	0	0	0	0	0	0	0	0	0	0	0	0	0	0	0	0	0	0	0	0	0	0	0	0	0	0	0	0	0	0	0	0	0	0	0	0	0	0	0	0
0	0	0	0	0	0	0	0	0	0	0	0	0	0	0	0	0	0	0	0	0	0	0	0	0	0	0	0	0	0	0	0	0	0	0	0	0	0	0	0	0
0	0	0	0	0	0	0	0	0	0	0	0	0	0	0	0	0	0	0	0	0	0	0	0	0	0	0	0	0	0	0	0	0	0	0	0	0	0	0	0	0
0	0	0	0	0	0	0.035087719	0	0	0	0	0	0	0	0	0	0	0	0	0	0	0	0	0	0	0	0	0	0	0	0	0	0	0	0	0	0	0	0	0	0
0	0	0	0	0	0	0	0	0	0	0	0	0	0	0	0	0	0	0	0	0	0	0	0	0	0	0	0	0	0	0	0	0	0	0	0	0	0	0	0	0
1.043169722	1.302856839	1.081797753	1.172457907	0.959725975	0.802128455	0.958801498	0.314606742	0.760587727	1.088428938	0.891204692	0.786516854	1.180724326	1.033707865	1.066677372	1.04494382	0.975501425	0.872962851	1.131324698	1.485018727	1.174157303	1.139810599	1.491564547	0.965530375	0.971098266	1.301266313	0.340989902	0.261491318	1.165329053	0.79363276	1.01784534	0.120130211	0.881450334	0.887640449	0.972205795	1.055588409	1.15909218	0.966292135	0.774178778	0.679775281	1.117470114
0	0	0	0	0	0	0	0	0	0	0	0	0	0	0	0	0	0	0	0	0	0	0	0	0	0	0	0	0	0	0	0	0	0	0	0	0	0	0	0	0
1.160199315	1	1.072142012	1.000422476	1.019680454	1.248370176	1.050887574	0.743195266	0.855566682	0.856422714	0.803080309	0.579881657	0.611217712	1.112426036	1.085565135	0.985207101	1.040069027	0.912884482	1.083872913	1.130769231	1.116272189	0.684011837	1	1	0.841346637	0.910261718	0.170595836	0.103281334	1.032121724	1.040692046	1.062304212	0.474478792	1.266222819	1.000800225	1.106197446	0.913734039	1.012465876	1.114792899	0.920025521	0.553254438	1.15579471
[illegible]	[illegible]	[illegible]	[illegible]	[illegible]	[illegible]	[illegible]	[illegible]	[illegible]	[illegible]	[illegible]	[illegible]	[illegible]	[illegible]	[illegible]	[illegible]	[illegible]	[illegible]	[illegible]	[illegible]	[illegible]	[illegible]	[illegible]	[illegible]	[illegible]	[illegible]	[illegible]	[illegible]	[illegible]	[illegible]	[illegible]	[illegible]	[illegible]	[illegible]	[illegible]	[illegible]	[illegible]	[illegible]	[illegible]	[illegible]	[illegible]

PF3D7-1372100_S0_Sub0_mip1	PF3D7-1372100_S0_Sub0_mip2	PF3D7-1372100_S0_Sub0_mip3	PF3D7-1372100_S0_Sub0_mip4	PF3D7-1372100_S0_Sub0_mip5	PF3D7-1372100_S0_Sub0_mip6	PF3D7-1372100_S0_Sub0_mip7	PF3D7-1372100_S0_Sub0_mip8	PF3D7-1372100_S0_Sub0_mip9	PF3D7-1372100_S0_Sub0_mip10	PF3D7-1372100_S0_Sub0_mip11	PF3D7-1372100_S0_Sub0_mip12	PF3D7-1372100_S0_Sub0_mip13	PF3D7-1372100_S0_Sub0_mip14	PF3D7-1372100_S0_Sub0_mip15	hrp3_S0_Sub0_mip0	hrp3_S0_Sub0_mip2	hrp3_S0_Sub0_mip3	hrp3_S0_Sub0_mip4	hrp3_S0_Sub0_mip5	hrp3_S0_Sub0_mip6	PF3D7-1372400_S0_Sub0_mip0	PF3D7-1372400_S0_Sub0_mip1	PF3D7-1372400_S0_Sub0_mip2	PF3D7-1372400_S0_Sub0_mip3	PF3D7-1372400_S0_Sub0_mip4	PF3D7-1372400_S0_Sub0_mip5	PF3D7-0831400_S0_Sub0_mip0	PF3D7-0831400_S0_Sub0_mip1	PF3D7-0831400_S0_Sub0_mip2	PF3D7-0831400_S0_Sub0_mip3	PF3D7-0831400_S0_Sub0_mip4	PF3D7-0831400_S0_Sub0_mip5	PF3D7-0831400_S0_Sub0_mip6	PF3D7-0831400_S0_Sub0_mip7	PF3D7-0831500_S0_Sub0_mip1	PF3D7-0831500_S0_Sub0_mip2	PF3D7-0831500_S0_Sub0_mip3	PF3D7-0831500_S0_Sub0_mip4	PF3D7-0831500_S0_Sub0_mip5	PF3D7-0831500_S0_Sub0_mip6	PF3D7-0831500_S0_Sub0_mip7	PF3D7-0831500_S0_Sub0_mip8	PF3D7-0831500_S0_Sub0_mip9
chr13	chr13	chr13	chr13	chr13	chr13	chr13	chr13	chr13	chr13	chr13	chr13	chr13	chr13	chr13	chr13	chr13	chr13	chr13	chr13	chr13	chr13	chr13	chr13	chr13	chr13	chr13	chr8	chr8	chr8	chr8	chr8	chr8	chr8	chr8	chr8	chr8	chr8	chr8	chr8	chr8	chr8	chr8	chr8
2837011	2837149	2837337	2837460	2837604	2837734	2837874	2838030	2838146	2838293	2838437	2838545	2838704	2838819	2838998	2840643	2840751	2840794	2841009	2841140	2841246	2852540	2852715	2852916	2853114	2853213	2853355	1344451	1344498	1344701	1344779	1344968	1345114	1345238	1345510	1353428	1353580	1353726	1353887	1354006	1354153	1354246	1354391	1354535
2837176	2837347	2837545	2837631	2837816	2837933	2838075	2838197	2838330	2838492	2838650	2838757	2838871	2839006	2839199	2840859	2840954	2841015	2841205	2841341	2841436	2852728	2852915	2853119	2853312	2853379	2853533	1344645	1344701	1344906	1344968	1345115	1345327	1345442	1345689	1353627	1353743	1353924	1354058	1354194	1354316	1354421	1354562	1354733
PF3D7-1372100	PF3D7-1372100	PF3D7-1372100	PF3D7-1372100	PF3D7-1372100	PF3D7-1372100	PF3D7-1372100	PF3D7-1372100	PF3D7-1372100	PF3D7-1372100	PF3D7-1372100	PF3D7-1372100	PF3D7-1372100	PF3D7-1372100	PF3D7-1372100	hrp3	hrp3	hrp3	hrp3	hrp3	hrp3	PF3D7-1372400	PF3D7-1372400	PF3D7-1372400	PF3D7-1372400	PF3D7-1372400	PF3D7-1372400	PF3D7-0831400	PF3D7-0831400	PF3D7-0831400	PF3D7-0831400	PF3D7-0831400	PF3D7-0831400	PF3D7-0831400	PF3D7-0831400	PF3D7-0831500	PF3D7-0831500	PF3D7-0831500	PF3D7-0831500	PF3D7-0831500	PF3D7-0831500	PF3D7-0831500	PF3D7-0831500	PF3D7-0831500
0	0	0	0	0	0	0	0	0	0	0	0	0	0	0	0	0	0	0	0	0	0	0	0	0	0	0	0.4	2.856948958	0.898210291	0.230769231	0.971428571	3.3	4.686192469	0	0.45	0.6	1.013308178	0.906122449	1.690916993	0	0	0.96	0.41686747
0	0	0	0	0	0	0	0	0	0	0	0	0	0	0	0	0	0	0	0	0	0	0	0	0	0	0	1.272727273	1.154322811	1.020693512	0.734265734	0.52987013	2.833333333	1.065043743	0.554545455	2.863636364	0.545454545	0.921189253	0.823747681	0.92231836	0.454545455	1.909090909	0.872727273	0.757940854
1.351045789	0.426229508	1.117552804	1.586206897	0.941810345	1.426901816	0.865359851	0.935682327	0.784500086	0.299560644	1.048275862	0.562909475	0.845908271	0.898434158	0.344827586	1.390804598	0.517241379	0.597701149	0.305488101	0.172413793	0.934190532	1.055690123	1.126453359	1.708374384	1.75862069	1.411563303	0.862068966	0.689655172	0.93042399	0.580739412	1.034482759	0.133990148	0.568965517	0.323185688	0	0.232758621	0.827586207	0.174708307	0.937368051	0.93291972	1.206896552	0.965517241	1.103448276	0.359368509
0	0	0	0	0	0	0	0	0	0	0	0	0	0	0	0	0	0	0	0	0	1.182046856	1.333352956	0.555102041	0.857142857	0.664534672	1.428571429	2.285714286	1.360451885	1.122762864	0.659340659	0.971428571	0.261904762	1.0041841	0	1.928571429	3	1.447583111	0.647230321	1.207797852	2.5	3	0.685714286	1.488812392
0	0	0	0	0	0	0	0	0	0	0	0	0	0	0	0	0	0	0	0	0	0.87809195	0.380957987	0.931778426	0.87755102	1.139202295	0.102040816	0.918367347	0.712617654	0.756146418	0.894819466	3.112536443	0.299319728	0.047818291	0	0.137755102	0.367346939	0	0.924614744	0.862712751	0.663265306	0.714285714	0.718367347	0.467912466
0	0	0	0	0	0	0	0	0	0	0	0	0	0	0	0	0	0	0	0	0	0	0	0	0	0	0	0.5	0.47615816	0.074850858	1.653846154	8.127619048	0	0	0	0	0	0	1.107482993	0.450911198	0.583333333	0.855555556	0.426666667	0
0	0	0	0	0	0	0	0	0	0	0	0	0	0	0	0	0	0	0	0	0	0	0	0	0	0	0	0.476190476	0.566854952	0.32078939	1.346153846	2.682993197	0	0	0	0.053571429	0	0.120631926	0.898931001	0.845458496	1.30952381	0.388888889	0.876190476	0.198508319
0.580449302	0	4.351212538	2.207070707	0.638888889	0.20899067	0.464730291	1.507488193	0	0	0	0.604606473	0	0	0	0	10	2.518518519	2.460876369	1.111111111	4.682485876	0.827432799	0	0.323809524	1.222222222	2.377557382	0	0.666666667	0.176354874	0.124751429	1.923076923	7.231746032	0	0	0	0.25	0	0	0.671201814	0.563638998	1.111111111	0.777777778	0.177777778	0.231593039
0	0	0	0	0	0	0	0	0	0	0	0	0	0	0	0	0	0	0	0	0	0	0	0	0	0	0	0.742647059	1.003666709	0.990673115	0.899321267	1.171428571	0.943627451	0.861432439	0	0.943014706	0.75	0.26077784	0.821728692	0.733559578	0.992647059	0.875	1.141176471	1.026842665
0	0	0	0	0	0	0	0	0	0	0	0	0	0	0	0	0	0	0	0	0	0.44251944	0.397558593	0.506618445	0.439240506	0.38391598	0.194620253	1	1.232586628	1.084389956	0.929649465	1.3360217	0.87721519	0.435993856	0.007721519	0.915664557	0.858227848	0.128266858	1.032291398	0.997426986	1.023734177	1.008649789	1.052151899	1.108128717
0	0	0	0	0	0	0	0	0	0	0	0	0	0	0	0	0	0	0	0	0	0	0	0	0	0	0	1.248091603	1.090438533	1.062767901	1.056958309	0.682224646	1.644402036	2.459356734	3.282824427	1.322519084	1.591603053	4.041629946	1.124006855	1.181060342	0.944656489	1.175572519	1.172519084	1.121723535
0	0	0	0	0	0	0	0	0	0	0	0	0	0	0	0	0	0	0	0	0	0	0	0	0	0	0	0.733333333	1.058129244	1.272464579	1.307692308	0.518095238	1.1	1.718270572	0.406666667	0.3	2.2	0.675538785	0.805442177	0.901822396	0.333333333	1.088888889	0.96	0.694779117
0	0	0	0	0	0	0	0	0	0	0	0	0	0	0	0	0	0	0	0	0	1.110501915	1.301773544	1.12481203	0.52631579	0.832417115	2.828947368	1.052631579	1.211279529	1.506865948	1.123481781	1.457142857	1.881578947	2.281435807	3.692105263	0.888157895	2.052631579	1.599960281	1.152524168	0.756462865	1.052631579	1.166666667	0.968421053	0.987317692
0	0	0	0	0	0	0	0	0	0	0	0	0	0	0	0	0	0	0	0	0	0.930861899	0.233336767	0.728571429	0.625	1.395522811	2.5	1.125	0.198399233	0.982417506	1.153846154	0.121428571	1.375	1.757322176	0.7625	0.5625	2.625	0.633317611	0.37755102	0.422729248	0.625	0.291666667	0.6	1.302710843
0.851108246	0.520814146	0.940711583	1.186414709	1.049859551	0.972158847	1.065224486	1.154208456	1.022494495	0.732072361	1.096629213	1.31450958	1.240348644	1.170992835	0.730337079	0.494382023	2.02247191	0.56928839	0.1493116	0.337078652	0.659531518	0.827432799	1.006756389	0.960513644	0.887640449	0.731734807	0.168539326	0.91011236	1.266188365	0.857841289	0.894554883	0.032744783	0.762172285	0.368576936	0	1.112359551	0.808988764	0.113854852	1.153863793	1.13994404	0.898876405	0.838951311	1.13258427	1.217815081
0	0	0	0	0	0	0	0	0	0	0	0	0	0	0	0	0	0	0	0	0	0	0	0	0	0	0	0.882591093	1.137381839	0.959121313	0.952974151	1.301792944	0.860998651	0.294072807	0.024696356	0.582995951	0.813765182	0	0.819301	1.08163921	0.860323887	0.746288799	0.984615385	1.021072143
1.2086397	0.036569987	1.081899122	1.036793975	1.104068047	1.233168616	1.095553537	1.050526852	0.915404835	0.596285212	0.916449704	1.126929225	0.980608457	1.083295403	0.029585799	0.850493097	0.414201183	2.889151874	1.336736395	0.905325444	1.644015645	0.961584626	0.751095866	0.981775148	1.036686391	0.933651553	0.079881657	0.984615385	1.09506985	1.020451928	0.918980428	1.410583263	0.631360947	0.371567924	0.007218935	0.657692308	0.592899408	0.077946783	0.970462505	0.934506788	0.985207101	0.867061144	1.056568047	0.873201683
[illegible]	[illegible]	[illegible]	[illegible]	[illegible]	[illegible]	[illegible]	[illegible]	[illegible]	[illegible]	[illegible]	[illegible]	[illegible]	[illegible]	[illegible]	[illegible]	[illegible]	[illegible]	[illegible]	[illegible]	[illegible]	[illegible]	[illegible]	[illegible]	[illegible]	[illegible]	[illegible]	[illegible]	[illegible]	[illegible]	[illegible]	[illegible]	[illegible]	[illegible]	[illegible]	[illegible]	[illegible]	[illegible]	[illegible]	[illegible]	[illegible]	[illegible]	[illegible]	[illegible]

PF3D7-0831600_S0_Sub0_mip0	PF3D7-0831600_S0_Sub0_mip1	PF3D7-0831600_S0_Sub0_mip2	PF3D7-0831600_S0_Sub0_mip3	PF3D7-0831600_S0_Sub0_mip4	PF3D7-0831600_S0_Sub0_mip7	[illegible]
chr8	chr8	chr8	chr8	chr8	chr8	[illegible]
1358227	1358417	1358512	1358685	1358866	1359316	[illegible]
1358416	1358620	1358684	1358896	1359070	1359495	[illegible]
PF3D7-0831600	PF3D7-0831600	PF3D7-0831600	PF3D7-0831600	PF3D7-0831600	PF3D7-0831600	[illegible]
1	1.7	1.814336373	0.802318841	2.571428571	0.975	[illegible]
1.363636364	0.257575758	0.494819011	2.188142292	1.402597403	1.772727273	[illegible]
1.091954023	1.563218391	0.688196555	0.276661669	0.709359606	0.784482759	[illegible]
0.714285714	1.619047619	2.332718194	0.573084886	0.734693878	0.928571429	[illegible]
0.782312925	0.925170068	0.851627277	0.245607808	0.419825073	0.397959184	[illegible]
0.555555556	0	0.483823033	0	0.085714286	0.108333333	[illegible]
0.674603175	0.134920635	0.431984851	0	0.551020408	0.348214286	[illegible]
0	0	0.20159293	0	0	0	[illegible]
0.882352941	1	0.840464643	0.825916454	0.586134454	0.860294118	[illegible]
1.016877637	1.118987342	1.020851288	0.67029169	1.000904159	0.975	[illegible]
1.08778626	1.167938931	0.907168186	1.347405686	1.236641221	1.035782443	[illegible]
0.555555556	0.566666667	1.209557582	0.534879227	0.857142857	0.433333333	[illegible]
1.754385965	2.75877193	0.95491388	1.37238749	0.812030075	1.154605263	[illegible]
1.041666667	1.770833333	1.587544326	1.002898551	1.928571429	0.8125	[illegible]
0.898876405	1.559925094	1.039675899	0.676111383	0.722311397	0.967696629	[illegible]
0.890688259	0.504723347	0.998986829	0.454755618	0.759976865	0.723684211	[illegible]
1.076923077	1.026035503	1.024187515	0.522219364	0.858157227	0.976923077	[illegible]
1.008230453	1.574074074	0.985565437	1.386723922	1.714285714	1.424382716	[illegible]
0.641025641	1.525641026	0.418693009	0.925752508	0.593406593	0.875	[illegible]
0.614035088	0.372807018	0.95491388	0.527841343	0.609022556	0.855263158	[illegible]
0.910087719	0.820175439	0.907168186	0.55423341	0.778195489	0.652138158	[illegible]
0.784313726	1	0.640354014	0.943904518	0.453781513	0.669117647	[illegible]
0.930232558	0.724806202	0.843877383	0.839635996	1.016611296	1.095930233	[illegible]
0.666666667	1.133333333	0.362867275	0.802318841	1.542857143	2.275	[illegible]
0.625	0.885416667	0.907168186	0.501449275	0.964285714	1.3203125	[illegible]
0.916666667	0.566666667	1.36075228	1.002898551	1.028571429	0.8125	[illegible]
1.185737977	1.353233831	1.173451385	1.117658086	1.343283582	1.26119403	[illegible]
1.145833333	1.121527778	0.755973489	1.922222222	1.285714286	0.947916667	[illegible]
1.047619048	0.80952381	1.088601824	0.343850932	0.514285714	0.371428571	[illegible]
0.666666667	1.133333333	0.725734549	0.802318841	0	1.7875	[illegible]
0.555555556	1.416666667	1.511946977	2.005797101	2.142857143	1.895833333	[illegible]
1.444444444	0.566666667	0.362867275	1.604637681	1.028571429	0.325	[illegible]
1.111111111	0.944444444	0.604778791	2.005797101	1.714285714	0.947916667	[illegible]
0.866666667	0.68	0.526157548	1.123246377	1.002857143	0.7475	[illegible]
1.987179487	1.416666667	1.116514691	0.771460424	1.384615385	1.25	[illegible]
1.666666667	0	1.088601824	0	0.514285714	0	[illegible]
1.060606061	1.287878788	0.742228516	2.552832675	1.051948052	0.443181818	[illegible]
1.666666667	2.428571429	0.518381821	1.719254658	1.102040816	1.625	[illegible]
1.044176707	1.16064257	0.808800552	1.063314126	1.115318417	1.233433735	[illegible]
0.980392157	1.5	1.387433697	1.651832907	2.268907563	2.007352941	[illegible]
1.344086022	1.005376344	0.760850737	1.68228144	2.073732719	0.891129032	[illegible]
1.388888889	1.227777778	1.390991219	2.273236715	2.657142857	2.058333333	[illegible]
[illegible]	[illegible]	[illegible]	[illegible]	[illegible]	[illegible]	[illegible]

PF3D7-0831750_S0_Sub0_mip0	PF3D7-0831750_S0_Sub0_mip1	PF3D7-0831750_S0_Sub0_mip2	PF3D7-0831750_S0_Sub0_mip3	PF3D7-0831750_S0_Sub0_mip4	PF3D7-0831750_S0_Sub0_mip5	PF3D7-0831750_S0_Sub0_mip6	hrp2_S0_Sub0_mip0	hrp2_S0_Sub0_mip1	hrp2_S0_Sub0_mip3	hrp2_S0_Sub0_mip4	hrp2_S0_Sub0_mip5	hrp2_S0_Sub0_mip6	hrp2_S0_Sub0_mip7	hrp2_S0_Sub0_mip8	PF3D7-0831900_S0_Sub0_mip0	PF3D7-0832000_S0_Sub0_mip0	PF3D7-0832000_S0_Sub0_mip1	PF3D7-0832000_S0_Sub0_mip2	PF3D7-0832000_S0_Sub0_mip3	PF3D7-0832000_S0_Sub0_mip4	PF3D7-0832000_S0_Sub0_mip5	PF3D7-0832100_S0_Sub0_mip0	PF3D7-0832100_S0_Sub0_mip1	PF3D7-0832100_S0_Sub0_mip2	PF3D7-0832100_S0_Sub0_mip3	PF3D7-0832100_S0_Sub0_mip4	PF3D7-0832100_S0_Sub0_mip5	PF3D7-0832100_S0_Sub0_mip6	PF3D7-0832100_S0_Sub0_mip7	PF3D7-0832200-2_S0_Sub0_mip0	PF3D7-0832200-2_S0_Sub0_mip1	PF3D7-0832200-2_S0_Sub0_mip2	PF3D7-0832200-2_S0_Sub0_mip3	PF3D7-0832200-2_S0_Sub0_mip4	PF3D7-0832300_S0_Sub0_mip0
chr8	chr8	chr8	chr8	chr8	chr8	chr8	chr8	chr8	chr8	chr8	chr8	chr8	chr8	chr8	chr8	chr8	chr8	chr8	chr8	chr8	chr8	chr8	chr8	chr8	chr8	chr8	chr8	chr8	chr8	chr8	chr8	chr8	chr8	chr8	chr8
1371664	1371800	1371969	1372168	1372345	1372430	1372614	1374269	1374297	1374430	1374468	1374619	1374630	1374763	1374871	1380756	1383785	1383870	1384048	1384194	1384364	1384390	1386570	1386610	1386788	1386959	1387025	1387189	1387281	1387448	1392258	1392429	1392665	1392878	1392909	1397685
1371848	1371998	1372175	1372367	1372509	1372615	1372807	1374450	1374521	1374625	1374661	1374805	1374822	1374985	1375047	1380927	1383949	1384053	1384225	1384363	1384560	1384585	1386754	1386808	1386973	1387131	1387224	1387362	1387480	1387625	1392431	1392612	1392854	1393071	1393108	1397861
PF3D7-0831750	PF3D7-0831750	PF3D7-0831750	PF3D7-0831750	PF3D7-0831750	PF3D7-0831750	PF3D7-0831750	hrp2	hrp2	hrp2	hrp2	hrp2	hrp2	hrp2	hrp2	PF3D7-0831900	PF3D7-0832000	PF3D7-0832000	PF3D7-0832000	PF3D7-0832000	PF3D7-0832000	PF3D7-0832000	PF3D7-0832100	PF3D7-0832100	PF3D7-0832100	PF3D7-0832100	PF3D7-0832100	PF3D7-0832100	PF3D7-0832100	PF3D7-0832100	PF3D7-0832200-2	PF3D7-0832200-2	PF3D7-0832200-2	PF3D7-0832200-2	PF3D7-0832200-2	PF3D7-0832300
0	0.45	1.496659243	2.18034398	0.36	0.848526863	1.2	0	0	0	0	0	0	0	0	0	0	0	0	0	0	0	0	0	0	0	0	0	0	0	0	0	0	0	0	0
1.96969697	1.840909091	1.020449484	1.486598168	0.654545455	1.928470143	0	0	0	0	0	0	0	0	0	0	0	0	0	0	0	0	0	0	0	0	0	0	0	0	0	0	0	0	0	0
[illegible]																																			

Supplementary Table 8. MIP variant calls, which were converted to .map and .hap files.

CHROM	chr13	chr13	chr13	chr13	chr13	chr13	chr13	chr13	chr13	chr13	chr13	chr13	chr13	chr13	chr13
POS	2781173	2781774	2783231	2783334	2783349	2783401	2783600	2783604	2783627	2783630	2783631	2783634	2788561	2788579	2788745
REF	T	T	T	T	T	G	A	A	G	A	G	G	G	A	T
ALT	A	C	C	A	C	A	T	T	T	T	T	T	A	C	C
lab_ID															
6		0	0	0	2	2							0	2	0
52													0	2	0
141	0	0											0	0	0
185													0	2	
228	0			0	0	2							0	0	0
311													0	2	0
342													0	2	0
386													0	0	
419	0	0	0	0	0	2	0	0	0	0	0	0	0	0	0
424	2	0	0	0	0	2	2	2	2	2	2	2	0	2	0
447	2	0	0			2	0	0	0	0	0	0	0	2	0
451		0													0
476	0	0	0	0	0	2	0	0	0	0	0	0	0	0	
485	0	0											0	0	0
488	0	0					0	0	0	0	0	0	0	0	
500	0	0	0	0		2	0	0	0	0	0	0	0	0	0
505	0	0	0	0	0	0	0	0	0	0	0	0	0	2	2
517	0	0	0			2	0	0	0	0	0	0	0	0	0
522															0
530	0	0											0	0	
535	0	2	0		0	2	0	0	0	0	0	0	0	2	0
558	0	0											0	0	
560	0	0											0	0	
584													0		0
598	0	0											0	0	
628		0					0	0	0	0	0	0	0	0	0
718	2	0	0		0	2	2	2	2	2	0	0	0	2	0
891	2	0	0	0	0	2	0	0	0	0	0	0	0	2	0
1029		0											0	2	0
1074													0		0
1142	0		0	0	0	2	2	2	2	2	0	0	0	0	
1156		0	0	2	0	2							0	2	0
1189		0	0		0	2	0	0	0	0	0	0	0	2	0
1197	2	0	0	0	0	2	0	0	0	0	0	0	0	2	0
1204		0											0	2	0
1211													0	2	0
1228		0	0	0	0	2	0	0	0	0	0	0	0	2	
1245			0		0	2	2	2	2	2	2	2	0		0
1259	2	0	0	0	0	2	0	0	0	0	0	0	0	2	0
1273	2	0	0	0		2	0	0	0	0	0	0	0	2	0
1278	0	0	0	0		2	0	0	0	0	0	0	0	0	0
1282		0	0	0	0	2	0	0	0	0	0	0	0	2	0
1284	0	0	0			2	0	0	0	0	0	0	0	2	0

[illegible]

chr8 1362996 G A	chr8 1363007 A T	chr8 1363027 C T	chr8 1363048 C T	chr8 1363110 G A	chr8 1363145 C T	chr8 1363159 T C	chr8 1363160 G C	chr8 1363211 G T	chr8 1363213 T C	chr8 1363220 A G	chr8 1363222 A T	chr8 1363235 G A	chr8 1363249 A T	chr8 1363303 T C	chr8 1363339 T C	chr8 1363341 A T	chr8 1363472 A G	chr8 1363485 C T	chr8 1363510 A G	chr8 1363532 A G	chr8 1363564 T C	chr8 1365677 A G	chr8 1366783 G T	chr8 1367104 C T	chr8 1367539 T A	chr8 1367546 G A	chr8 1367595 T A	chr8 1372081 G T	chr8 1372401 T G
0	0	0	0	0	2	2	2																0	0	2	0	0		
0	0	0	0	0	2	2	2	2	2	2	2	2	2	0	0	0	2	2	2	2	2	0	0	0	2	0	0	2	
2	0	0	0	0	2	2	2	0	0	0	0	0	0	2	2	2						0	0	0				2	2
0	0	0	0	0	2	2	2	2	2	2	2	2	2	0	0	0						0	0	0				2	
0	0	0	0	0	2	2	2	2	2	2	2	2	2	0	0	0	0	0	0	0	0	0	0	0	0	0	0	0	0
[illegible]	[illegible]	[illegible]	[illegible]	[illegible]	[illegible]	[illegible]	[illegible]	[illegible]	[illegible]	[illegible]	[illegible]	[illegible]	[illegible]	[illegible]	[illegible]	[illegible]	[illegible]	[illegible]	[illegible]	[illegible]	[illegible]	[illegible]	[illegible]	[illegible]	[illegible]	[illegible]	[illegible]	[illegible]	[illegible]

Supplementary Table 9. ENA accession numbers of previously published WGS data.

Sample	ENA
QS0056-C	ERR1035493
QS0087-C	ERR1035520
QS0104-C	ERR1035536
QS0109-C	ERR1045266
QS0110-C	ERR1045267
QS0116-C	ERR1045271
QS0126-C	ERR1045280
QS0127-C	ERR1045281
QS0128-C	ERR1045282
QS0129-C	ERR1045283
QS0132-C	ERR1045286
QS0133-C	ERR1045287
QS0135-C	ERR1045288
QS0144-C	ERR1045295
QS0154-C	ERR1106575
QS0155-C	ERR1106606
QS0156-C	ERR1106576
QS0157-C	ERR1106577
QS0159-C	ERR1106579
QS0162-C	ERR1106581
QS0163-C	ERR1106582
QS0166-C	ERR1106584
QS0168-C	ERR1106586
QS0169-C	ERR1106587
QS0170-C	ERR1106590
HB3	ERR012788
DD2	ERR012840
3D7	ERR019061

Supplementary Table 10. Summary statistics of WGS coverage for samples sequenced in this study. Abbreviations: % Coverage, percent of the *P. falciparum* 3D7 (v3) genome with at least 1, 5, or 10 aligned reads per locus.

lab_ID	% Coverage 1X	% Coverage 5X	% Coverage 10X	median coverage value
1156	77.0	46.9	32.5	4
1194	88.1	69.0	53.2	11
1197	91.2	79.3	66.8	20
1208	92.3	84.1	74.7	30
1220	89.4	73.0	58.5	14
1225	79.0	46.6	30.4	4
1268	92.7	87.0	79.3	33
1284	92.2	83.0	72.8	26
1314	90.5	76.8	63.5	17
1333	85.7	60.2	42.2	7
5338	92.4	83.9	73.4	24
9592	93.5	89.2	82.9	36
9821	88.8	70.5	55.4	12
10809	76.3	46.4	33.2	4
10839	46.1	7.9	2.4	0
10885	91.2	78.3	64.1	16
10901	93.3	88.1	81.4	38
10905	91.1	79.4	67.7	21
10924	93.2	88.6	82.0	36
10937	93.0	85.7	76.6	30
10941	87.4	68.0	54.2	12
10957	91.7	80.5	68.5	21
12129	87.5	68.9	55.3	12
99112	92.9	86.7	78.6	31

Supplementary Table 11. RDT results from 12,572 enrolled subjects.

	**SD Bioline**					
**CareStart**	HRP2 and Pf-LDH	HRP2 only	Pf-LDH only	Negative	Missing	Invalid
HRP2 and Pv-LDH	536	31	7	18	0	1
HRP2 only	1000	549	49	123	1	3
Pv-LDH only	5	1	39	540	5	0
Negative	6	22	322	9297	0	7
Missing	0	0	0	0	1	0
Invalid	0	1	1	1	0	6

	P. falciparum **Beta tubulin (*PfBTubulin*)**			***P. falciparum*- specific *pfldh***		
Adapted from:	Primers: Price RN et al. Lancet 2004. 364:438-447.			Pickard AL et al. Antimicrob Agents Chemo 2003. 47(8):2418-2423.		
	Flap: Afonina I et al. Biotechniques 2007. 770-774.					
	Combined assay: Parr JB et al. Malar J 2018. 17:137.					
Forward Primer (5'->3')	AATAAATCATAATGATGTGCGCAAGTGATCC			ACGATTTGGCTGGAGCAGAT		
Reverse Primer (5'->3')	AATAAATCATAATCCTTTGTGGACATTCTTCCTC			TCTCTATTCCATTCTTTGTCACTCTTTC		
Probe (5'->3')				FAM/ AGTAATAGTAACAGCTGGATTTACCAAGGCCCCA /TAMRA		
Cycling conditions:	Temp	Duration	No. Cycles	Temp	Duration	No. Cycles
	50C	2 min	x1	50C	2 min	x1
	95C	10 min	x1	95C	10 min	x1
	95C	15 sec	x45	95C	15 sec	x40
	60C	1 min		60C	1 min	
Reaction conditions:	SYBR Green MM			Roche FastStart Universal Probe Master (Rox)		
	Fwd primer	200 nM		Fwd primer	200 nM	
	Rev primer	200 nM		Rev primer	200 nM	
				Probe	200 nM	
	Template	2 µl		Template	2 µl	
	Total volume	12 µl		Total volume	12 µl	

	Pfhrp2 **- exon 2**			***Pfhrp3*** **- exon 2 (single-step)**		
Adapted from:	Baker J et al. J Infect Dis 2005. 192:870-877.			Baker J et al. J Infect Dis 2005. 192:870-877.		
Forward Primer (5'->3')	CAAAAGGACTTAATTTAAATAAGAG			AATGCAAAAGGACTTAATTC		
Reverse Primer (5'->3')	AATAAATTTAATGGCGTAGGCA			TGGTGTAAGTGATGCGTAGT		
Cycling conditions:	Temp	Duration	No. Cycles	Temp	Duration	No. Cycles
	94C	10 min	x1	94C	10 min	x1
	94C	50 sec		94C	50 sec	
	55C	50 sec	x45	55C	50 sec	x45
	70C	60 sec		70C	60 sec	
	72C	7 min	x1	72C	7 min	x1
Reaction conditions:	AmpliTaq Gold 360 Master Mix			AmpliTaq Gold 360 Master Mix		
	Fwd primer	200 nM		Fwd primer	200 nM	
	Rev primer	200 nM		Rev primer	200 nM	
	Template	10 µl		Template	10 µl	
	Total volume	25 µl		Total volume	25 µl	
