# Supplementary material for: Plasmodium falciparum is evolving to escape malaria rapid diagnostic tests in Ethiopia
Source: Nat Microbiol. 2021 Sep 27;6(10):1289–99. doi: 10.1038/s41564-021-00962-4 (PMC8478644; doi:10.1038/s41564-021-00962-4)
Supplement: Supplementary file 2 — Reporting Summary [file 41564_2021_962_MOESM2_ESM.pdf]

nature research

Corresponding author(s): Sindew M. Feleke, Jane Cunningham, and Jonathan B Parr

Last updated by author(s): Aug 4, 2021

Reporting Summary

Nature Research wishes to improve the reproducibility of the work that we publish. This form provides structure for consistency and transparency in reporting. For further information on Nature Research policies, see our Editorial Policies and the Editorial Policy Checklist.

Statistics

For all statistical analyses, confirm that the following items are present in the figure legend, table legend, main text, or Methods section.

n/a	Confirmed	
☐	☒	The exact sample size (*n*) for each experimental group/condition, given as a discrete number and unit of measurement
☐	☒	A statement on whether measurements were taken from distinct samples or whether the same sample was measured repeatedly
☐	☒	The statistical test(s) used AND whether they are one- or two-sided<br>*Only common tests should be described solely by name; describe more complex techniques in the Methods section.*
☐	☒	A description of all covariates tested
☐	☒	A description of any assumptions or corrections, such as tests of normality and adjustment for multiple comparisons
☐	☒	A full description of the statistical parameters including central tendency (e.g. means) or other basic estimates (e.g. regression coefficient) AND variation (e.g. standard deviation) or associated estimates of uncertainty (e.g. confidence intervals)
☐	☒	For null hypothesis testing, the test statistic (e.g. *F*, *t*, *r*) with confidence intervals, effect sizes, degrees of freedom and *P* value noted<br>*Give P values as exact values whenever suitable.*
☒	☐	For Bayesian analysis, information on the choice of priors and Markov chain Monte Carlo settings
☒	☐	For hierarchical and complex designs, identification of the appropriate level for tests and full reporting of outcomes
☒	☐	Estimates of effect sizes (e.g. Cohen's *d*, Pearson's *r*), indicating how they were calculated

Our web collection on statistics for biologists contains articles on many of the points above.

Software and code

Policy information about availability of computer code

Data collection	Data was collected in the field using paper forms and double entered into Epi Info (v3.2). Discrepancies were cross-checked against the hard-copy paper forms and resolved by consensus.
Data analysis	Custom code used during data analysis is available through GitHub at https://doi.org/10.5281/zenodo.5160363. Statistical analysis was performed using R (version 3.6.0, R Core Team, Vienna, Austria, 2019; www.R-project.org). Prevalence estimates were generated using the asbio package (v1.5-5). 95% confidence intervals were calculated using the binom.confint package (v1.1-1), and unweighted Cohen's kappa estimates calcualted using the psych (v1.8.1) and epiR (v1.0-15) packages. ArcGIS (Desktop Version 10.5, ESRI, Redlands, CA, 2016) was utilized for mapping, with additional annotation performed using PowerPoint (version 16.31, Microsoft, Redmond, WA, 2019).<br><br>MIP design and sequencing fastq file processing were performed using MIPTools (v0.19.12.13), which uses the MIPWrangler algorithm (v1.2.0), bwa (v0.7.17), and freebayes (v1.3.1). Structural profile groups were assigned using the hierarchical clustering algorithm AgglomerativeClustering of the Python module Scikit-learn (v0.20).<br><br>Genome sequencing fastq files were processed and analysed using Trimmomatic (v.0.39), bowtie2 (v2.3.0), sambamba (v0.7.1), samtools (v1.9), and freebayes (v1.3.1). Publicly available parasite genomes from Ethiopia were downloaded using fasterq-dump (v2.10.8).<br><br>Extended haplotype homozygosity (EHH) statistics were calculated using the rehh package (version 3.1.2); plots were annotated using Inkscape (version 0.92). Complexity of infection (COI) was calculated using McCOILR (v1.3.0, https://github.com/OJWatson/McCOILR), an Rcpp wrapper for THE REAL McCOIL.

For manuscripts utilizing custom algorithms or software that are central to the research but not yet described in published literature, software must be made available to editors and reviewers. We strongly encourage code deposition in a community repository (e.g. GitHub). See the Nature Research guidelines for submitting code & software for further information.

Data

Policy information about availability of data

All manuscripts must include a data availability statement. This statement should provide the following information, where applicable:

- Accession codes, unique identifiers, or web links for publicly available datasets
- A list of figures that have associated raw data
- A description of any restrictions on data availability

MIP and genomic sequencing data is available through the Sequence Read Archive (PRJNA742125). De-identified datasets generated during the current study and used to make all figures are available as supplementary files or tables.

Extended Data Figure 6-7 were derived from genomic sequencing data made publicly available by MalariaGEN (https://www.malariagen.net/data, downloaded Sep 19 2020). Extended Data Figure 8 was derived from genomic sequencing data generated during this study and publicly available through MalariaGEN.

The hg38 human genome used during whole-genome sequencing analysis was downloaded from the US National Institutes of Health National Center for Biotechnology and Information database on December 2, 2015 (available at https://www.ncbi.nlm.nih.gov/assembly/GCF_000001405.26/).

Field-specific reporting

Please select the one below that is the best fit for your research. If you are not sure, read the appropriate sections before making your selection.

☒ Life sciences ☐ Behavioural & social sciences ☐ Ecological, evolutionary & environmental sciences

For a reference copy of the document with all sections, see nature.com/documents/nr-reporting-summary-flat.pdf

Life sciences study design

All studies must disclose on these points even when the disclosure is negative.

Sample size	Sample sizes were derived from the WHO "Template protocols to support surveillance and research for pfhrp2/pfhrp3 gene deletions," available at https://www.who.int/malaria/publications/atoz/hrp2-deletion-protocol/en/.
Data exclusions	Sources of data and samples included in the study are outlined in Figure 2. Dried blood spot samples were only collected from a subset of subjects based on WHO protocols. Molecular, immunological, and sequencing assays were performed on random subsets selected by EPHI. As outlined in the Methods, most analyses were limited to samples that could be matched unambiguously across datasets. For example, any DBS samples found to have identical participant IDs were excluded from analysis. Similarly, DBS labeled with a participant and region ID that did not match clinical data were excluded from most analyses. These accounted for a minority of subjects. Discordances in participant IDs and DBS sample labels were resolved whenever possible.
Replication	All PCR assays were performed in duplicate. Deletion calls made by PCR were limited to samples with >100 parasites/µL, with negative pfhrp2 or pfhrp3 bands in both replicates, and positive by a final confirmatory real-time PCR assay as described in the Methods.<br><br>Comparison of whole-genome sequencing and MIP calls was undertaken for 14 samples as outlined in the Results. Sequencing (MIP and WGS) was performed across multiple flow cells.<br><br>To increase confidence in pfhrp2/3 deletion calls, multiple confirmatory methods were employed, including PCR, MIP sequencing, WGS, and an HRP2 immunoassay.<br><br>Results were compared across platforms, and concordance/discordance between methods included in the Results. While most calls were concordant, we did observe samples with discordance results across different assays. This was not unexpected because the assay targets are different in some cases (ex: HRP2 immunoassay detects HRP2 or HRP3 antigen that can linger after clearance of parasitemia, whereas the molecular methods detect parasite DNA that clears rapidly after resolution of infection). However, we cannot exclude the possibility that some discordance may have been introduced by ambiguous sample labeling and/or processing during the conduct of the field work. We overcame this by restricting analyses to samples with complete meta-data and no ambiguity when merged with molecular and/or antigen data (see Figure 2), and by employing a conservative approach to prevalence estimates as described in the Results, Methods, and Discussion.
Randomization	As outlined in the WHO protocol, any subject presenting to study health facilities with symptoms of malaria was eligible for enrollment. Randomization was not performed. We did not undertake detailed analyses of covariates, except as shown in Supplementary Table 1 in which we stratified by pfhrp2/3 status.
Blinding	Field staff were not blinded to malaria RDT results because they were used to inform clinical care according to national guidelines.<br><br>Pfhrp2/3 deletion calls using MIP sequencing were made by an investigator who was blinded to clinical data (including RDT results), HRP2 immunoassay results, and pfhrp2/3 deletion calls using PCR.

Reporting for specific materials, systems and methods

We require information from authors about some types of materials, experimental systems and methods used in many studies. Here, indicate whether each material, system or method listed is relevant to your study. If you are not sure if a list item applies to your research, read the appropriate section before selecting a response.

Materials & experimental systems

n/a	Involved in the study
☒	☐ Antibodies
☒	☐ Eukaryotic cell lines
☒	☐ Palaeontology and archaeology
☒	☐ Animals and other organisms
☐	☒ Human research participants
☒	☐ Clinical data
☒	☐ Dual use research of concern

Methods

n/a	Involved in the study
☒	☐ ChIP-seq
☒	☐ Flow cytometry
☒	☐ MRI-based neuroimaging

Human research participants

Policy information about studies involving human research participants

Population characteristics	Subjects of all ages and genders who presented to health facilities with symptoms of malaria were eligible for enrollment. All subjects provided informed consent. Participants were not compensated. See Methods for details.<br><br>Among 12,572 study subjects, the median age was 19 years (interquartile range 8-30). 5,555 (44.2%) were female. See Table 1 for details.
Recruitment	This was a cross-sectional survey conducted at 108 health facilities located in eleven districts within three regions of Ethiopia near its borders with Eritrea, Sudan, and South Sudan. Subjects were enrolled as noted above, but DBS samples for molecular and/or immunological analysis were only collected from a subset. The study was designed to collect DBS from any subject with a 'discordant RDT result' suggestive of infection by pfhrp2/3-deleted P. falciparum (HRP2 bands negative on two distinct RDTs, Pf-LDH band positive) and from 10-20% of subjects with positive HRP2 bands. The focus on discordant RDT results is an intentional component of the WHO protocol, included to allow real-time, efficient signaling to malaria control programs.<br><br>This was a pragmatic survey conducted as part of routine malaria care at government health facilities. As such, DBS were not collected/available from all subjects with discordant RDT results (see Results). In addition, not all samples underwent pfhrp2/3 deletion PCR genotyping (to avoid the risk of misclassification due to low P. falciparum DNA concentrations), HRP2 immunoassay, or sequencing. Among those sequenced, only a subset had sufficient UMI depth of coverage to be included in analysis.<br><br>These features and the study design could introduce selection bias when estimating prevalence. For example, a disproportionate number of subjects with a 'discordant RDT result' had DBS sent for molecular and antigen testing, which could lead to over-estimates of pfhrp2/3 deletion prevalence. We accounted for this by estimating the frequency of falciparum malaria cases with false-negative RDT results due to pfhrp2/3 deletions using RDT data from the highest-level dataset (12,572 participants - see Figure 2). This dataset was derived from all enrolled subjects and therefore felt to be most representative of people presenting for routine malaria care.<br><br>Figure 2 displays how subjects and samples were included in analyses, and denominators are included throughout the Results to avoid ambiguity.
Ethics oversight	Ethical approval was obtained from the Ethiopia Public Health Institute (EPHI) Institutional Review Board (IRB; protocol EPHI-IRB-033-2017) and WHO Research Ethics Review Committee (protocol: ERC.0003174 001).

Note that full information on the approval of the study protocol must also be provided in the manuscript.
